# Coordination–Entropy Regulation: Toward Unified Design of Hydrogel Electrolytes for Practical Wide-Temperature Zinc-Ion Batteries

**DOI:** 10.1007/s40820-026-02293-7

**Published:** 2026-07-02

**Authors:** Cong Wang, Hong Zhang, Peng Wang, Ke Lu, Chun Cheng Yang, Qing Jiang

**Affiliations:** 1 Key Laboratory of Automobile Materials (Jilin University), Ministry of Education and School of Material Science and Engineering, Jilin University, Changchun, 130022 People’s Republic of China; 2 Key Laboratory of Physics and Technology for Advanced Batteries (Ministry of Education), State Key Laboratory of Superhard Materials, College of Physics, Jilin University, Changchun, 130012 People’s Republic of China

**Keywords:** Aqueous zinc-ion batteries, Hydrogel electrolytes, Coordination–entropy strategy, Interfacial stabilization, Wide-temperature operation

## Abstract

Hydrogel electrolytes have emerged as promising alternatives to conventional aqueous electrolytes in zinc-ion batteries owing to their high ionic conductivity and mechanical robustness. However, hydrogel electrolytes still suffer from stability challenges under wide-temperature conditions. At low temperatures, the freezing of water and the ordering of hydrogen-bond networks restrict ion transport and strengthen the hydrated structure of Zn^2+^. At high temperatures, water evaporation and intensified parasitic reactions can destroy the electrode–electrolyte interface and induce structural degradation. These coupled thermodynamic and kinetic effects indicate that achieving stable wide-temperature operation requires coordinated regulation of ion transport, solvation thermodynamics and interfacial dynamics. To address this challenge, the coordination–entropy (C-E) regulation framework is proposed as a unified design concept. Within this framework, coordination regulation reconstructs Zn^2+^ solvation environments and lowers desolvation barriers, while entropy regulation increases the diversity of accessible ionic configurations and transport states across multiple structural scales. Their intrinsic coupling enables stable ion transport and interfacial chemistry under temperature variation. This review systematically summarizes recent advances in polymer, salt, cosolvent and filler regulation strategies directed by the C-E framework, aiming to establish a unified physicochemical framework for wide-temperature hydrogel electrolytes and guide the development of durable, high-energy–density zinc-ion batteries.

**Graphical abstract:**

## Highlights

- Critical limitations of hydrogel electrolytes operation under wide-temperature conditions are systematically summarized.
- A coordination–entropy regulation framework is proposed to unify Zn^2+^ solvation chemistry, electrolyte thermodynamics and interfacial kinetics.
- Future directions for practical wide-temperature zinc batteries are comprehensively discussed.

## Graphical abstract

**Figure:**
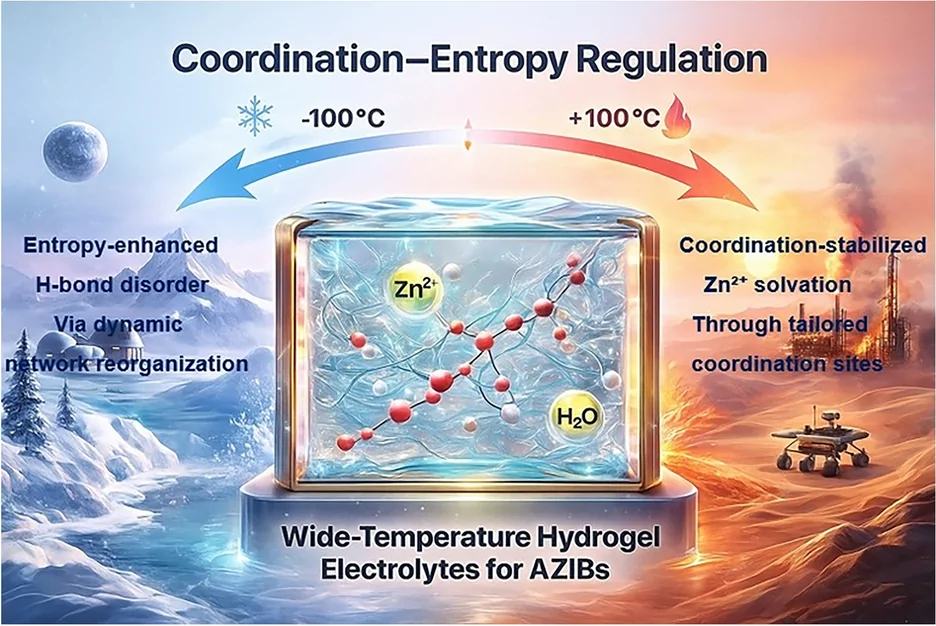

## Introduction

The global energy landscape is undergoing a transformative transition toward sustainable development, characterized by deep decarbonization and large-scale integration of renewable energy sources [1–3]. Safe, durable and environmentally benign energy storage technologies are indispensable for stabilizing intermittent power generation and improving overall energy utilization efficiency [4–7]. Although lithium-ion batteries (LIBs) currently dominate the electrochemical storage market, their large-scale deployment faces intrinsic constraints, including limited lithium resources, flammable organic electrolytes and environmental concerns arising from extraction and recycling processes [8–11]. These limitations have stimulated growing interest in sustainable and resource-abundant alternatives for next-generation energy storage systems [12–15].

Among the emerging candidates, aqueous zinc-ion batteries (AZIBs) have attracted particular attention owing to the abundance and low cost of zinc, along with the high theoretical capacity (820 mAh g^−1^), low redox potential (− 0.76 V vs. standard hydrogen electrode, SHE) and excellent reversibility of Zn anodes [16–19]. Inherently safe and nonflammable aqueous electrolytes make AZIBs highly attractive for grid-scale and wearable applications [20–22]. However, their practical deployment remains hindered by electrolyte instability and interfacial degradation, which are strongly amplified under extreme temperature conditions [23–25]. At low temperatures, electrolyte freezing suppresses ion mobility and dramatically increases charge-transfer resistance, while at elevated temperatures, water evaporation and corrosion accelerate hydrogen evolution and parasitic side reactions [26]. These effects lead to polarization, dendrite formation and rapid capacity decay, ultimately compromising safety and reversibility [27–29]. Consequently, designing electrolytes that maintain high ionic conductivity, interfacial compatibility and mechanical robustness across a broad-temperature range is essential for achieving all-climate operation [30–32].

To address these challenges, extensive strategies have been developed from both the aspects of electrolyte regulation and electrode design [33–38]. Similar interfacial and coordination–regulation principles have also been widely recognized in electrochemical systems, where tuning electronic structures, coordination environments and reaction intermediates play a key role in governing reaction kinetics and stability [39–41]. Increasing salt concentration, introducing deep-eutectic solvents (DESs) and adding functional additives can suppress water activity and reconstruct the Zn^2+^ solvation structure, yet often at the expense of enhancing viscosity, reducing ionic mobility or increasing cost [38, 42–44]. On the electrode side, surface coatings and three-dimensional current collectors have been explored to inhibit dendrite growth and surface corrosion, but these approaches typically involve additional interfacial resistance [45–47]. Therefore, it is a critical challenge to achieve high ionic conductivity and stable interfaces under wide-temperature variations [48].

In this context, hydrogel polymer electrolytes (HPEs) have emerged as a promising solution to bridge liquid and solid systems [49, 50]. Their unique three-dimensional “liquid-embedded-solid” architecture confines the aqueous phases within the polymeric networks, combining the fast ion transport of the liquids with the structural integrity of the solids [51–53]. Through hydrogen-bond regulation and molecular confinement, HPEs can effectively suppress water activity, inhibit side reactions and ensure the stable Zn^2+^ transport even under extreme thermal conditions [54–56]. In addition, the tunable chemical structures and side-chain functional polymer backbones enable precise regulation of the solvation structure, hydrogen-bond dynamics and mechanical flexibility [57–59]. These characteristics make HPEs a multifunctional platform for the development of wide-temperature, flexible and durable aqueous batteries.

However, the current research on HPEs still lacks a unified theoretical framework that links molecular solvation, polymer-network dynamics and interfacial processes [17, 50]. Moreover, the coupling among desolvation thermodynamics, charge-transfer kinetics and entropy-related state evolution remains insufficiently understood, which limits the rational design of multiscale HPE systems [60]. To bridge this gap, the coordination–entropy (C-E) regulation framework is proposed as a theory-informed design framework that distinguishes coordination regulation of local interaction energetics from entropy regulation of accessible-state diversification. Rather than serving as a predictive thermodynamic theory, it provides a cross-scale design guideline linking Zn^2+^ solvation chemistry, polymer-network adaptability and interfacial transport behavior across wide-temperature ranges. Within this framework, coordination regulation governs Zn^2+^ solvation and interfacial desolvation behavior, while entropy regulation increases the diversity of accessible ionic and transport states across multiple structural scales. Their intrinsic coupling promotes homogeneous ion flux and improved interfacial stability under wide-temperature conditions [61, 62].

**Fig. 1:**
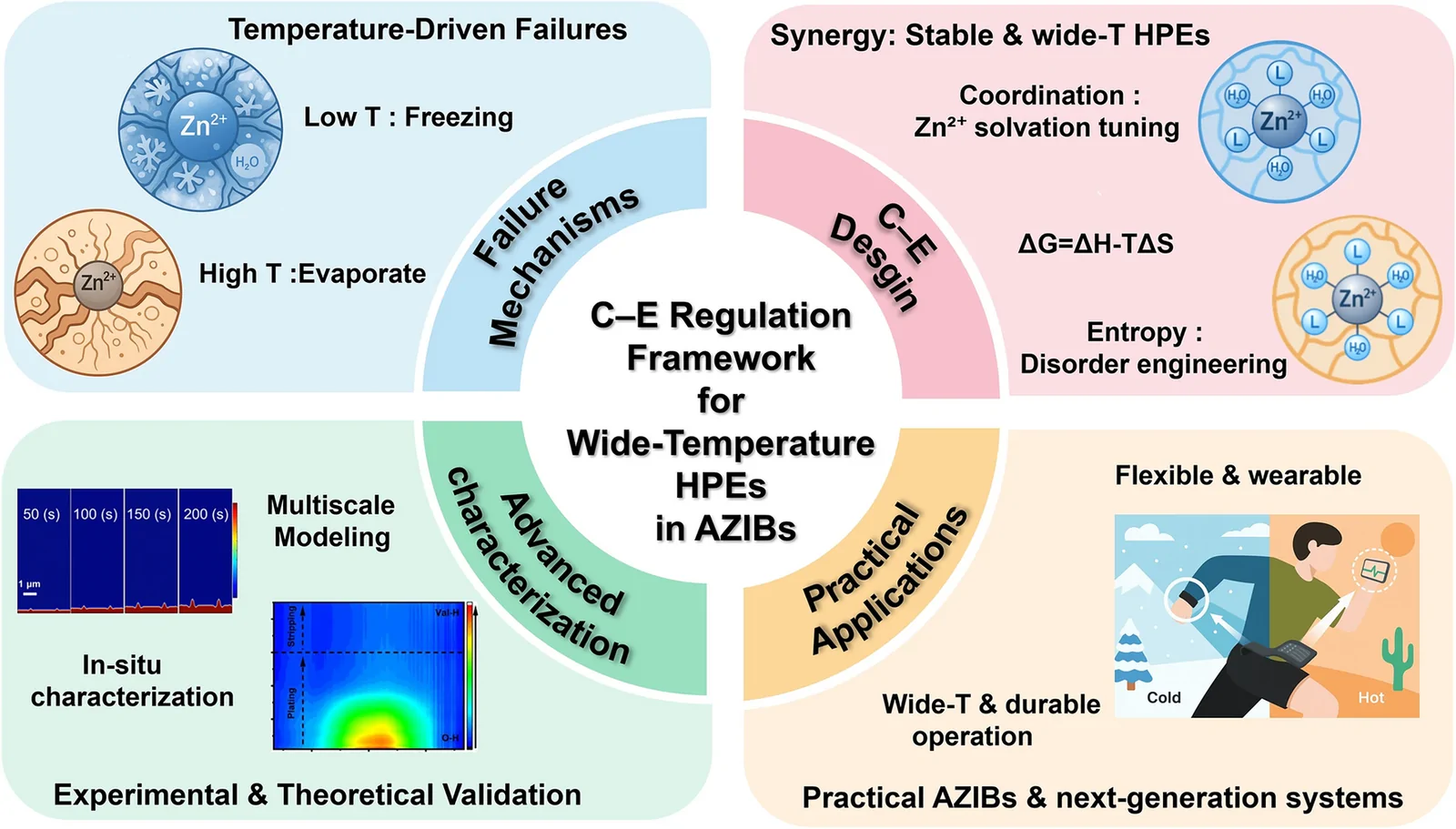
Schematic illustration of the regulation framework guiding the design and application of wide-temperature HPEs in AZIBs

Herein, this review systematically analyzes the failure mechanisms and interface evolution of aqueous electrolytes. The C-E regulation framework is further elaborated to reveal the synergistic coupling between molecular-level coordination regulation and entropy regulation, thereby establishing a theoretical foundation for rational electrolyte design over wide-temperature ranges. Guided by this framework, recent advances in polymer system regulation, salt system regulation, cosolvent regulation, filler regulation and integrated multiscale C-E regulation are systematically summarized, with particular emphasis on the intrinsic coupling between coordination regulation and entropy regulation across different structural scales. Additionally, in situ/operando characterization and multiscale simulations are emphasized as powerful tools to elucidate the dynamic coupling between coordination regulation and entropy regulation. Finally, the challenges and future directions for wide-temperature HPEs are discussed, providing design principles and theoretical guidance for the development of durable, flexible and high-energy–density AZIBs (Fig. 1).

## Failure Mechanisms and Interfacial Challenges of AZIBs

Electrolytes in AZIBs not only serve as ion-conducting media but also govern the thermodynamic and kinetic processes associated with Zn^2+^ solvation, ion transport and electrode–electrolyte interfacial reactions [63, 64]. The strong hydration of Zn^2+^ and the high activity of water create complex couplings among solvation chemistry, interfacial electrochemistry and mass transport, which collectively determine the stability of both the anode–electrolyte interface (AEI) and the cathode–electrolyte interface (CEI) [65]. Understanding these coupled processes is therefore essential for elucidating the intrinsic instability of aqueous systems and guiding rational electrolyte design. In this context, liquid electrolytes provide a useful baseline for identifying the fundamental failure mechanisms of AZIBs. By analyzing the degradation pathways of liquid electrolytes, the roles of Zn^2+^ solvation, water reactivity and interfacial side reactions in battery failure can be clarified [66, 67]. Building on this baseline, HPEs introduce polymer networks that confine water molecules, regulate ion coordination environments and modify interfacial transport behavior. To elucidate how these factors influence battery stability, this section first examines the multiscale degradation mechanisms in liquid electrolytes and then analyzes the evolution of the anode–electrolyte interface and cathode–electrolyte interface when the electrolyte medium transitions from liquid to hydrogel. Finally, the opportunities and challenges associated with HPEs are summarized.

### Multiscale Failure Mechanisms of Liquid Electrolytes

**Fig. 2:**
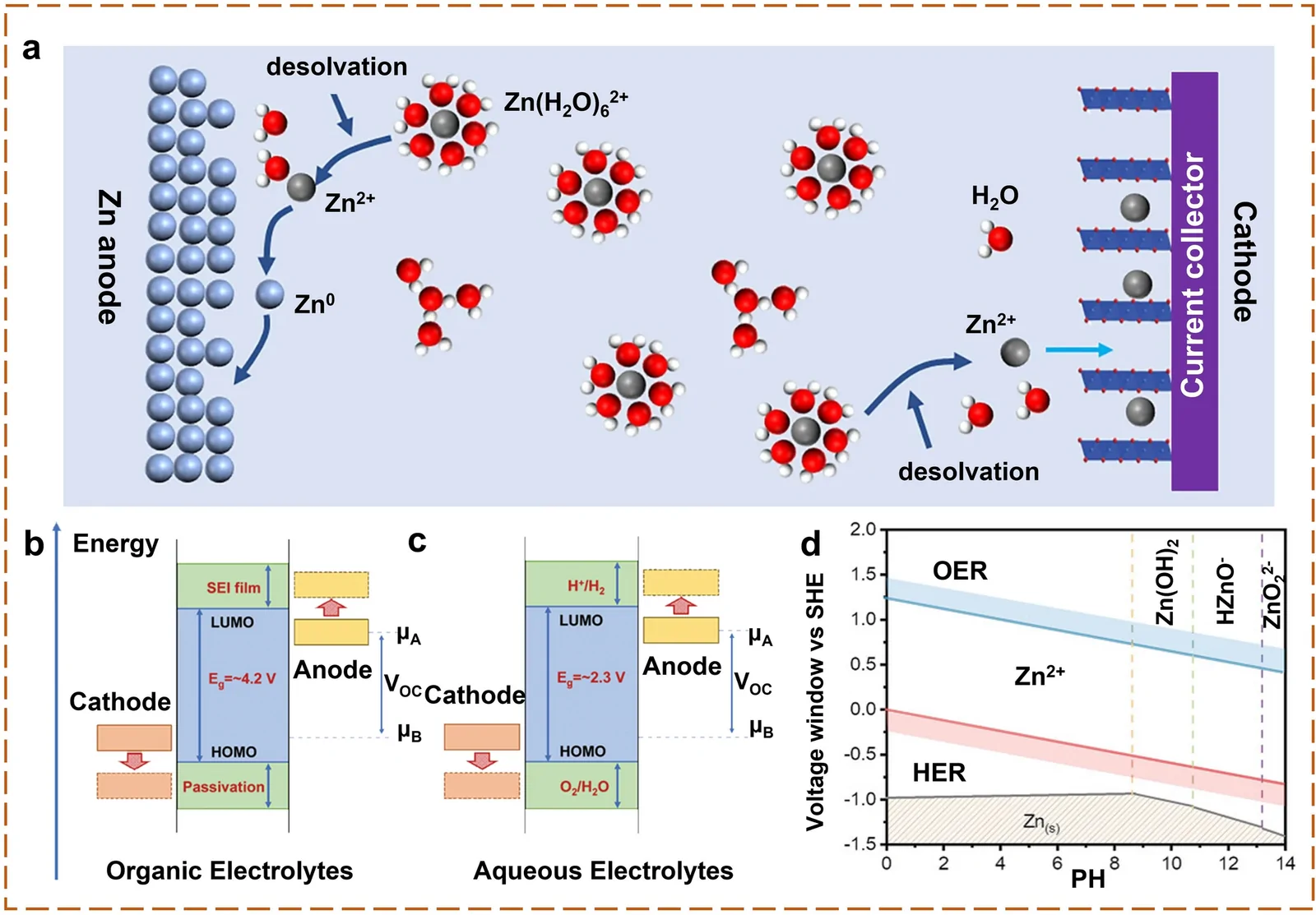
Schematic summary of Zn^2+^ solvation configuration and electrochemical instability in liquid electrolytes.** a** Configuration of hydrated Zn^2+^ in the electrolyte and its insertion/extraction at electrode interfaces [69]. Copyright 2023, Springer Nature. **b** Energy-level alignment of liquid electrolytes with solid electrodes. **c** Energy-level alignment of solid electrolytes with liquid or gaseous reactants [68]. Copyright 2013, American Chemical Society. **d** Pourbaix diagram of Zn in aqueous solutions, showing suppressed by-product formation and dendrite growth at pH < 8.5 [35]. Copyright 2020, American Chemical Society

In conventional aqueous electrolytes, the electrochemical behavior of AZIBs is strongly governed by the solvation chemistry of Zn^2+^ and the reactivity of water. Water molecules in the electrolyte exist in multiple states, including hydrogen-bonded clusters, Zn^2+^- or anion-coordinated species and a small fraction of free water [33, 68]. The dynamic equilibrium among these species determines the solvation configuration of Zn^2+^ and governs the continuity of ion transport. Typically, Zn^2+^ adopts an octahedral hydration structure, [Zn(H_2_O)_6_]^2+^, in aqueous environments (Fig. 2a) [69]. During battery operation, Zn^2+^ storage involves a sequence of coupled thermodynamic and kinetic processes, including Zn stripping/plating, bulk solvation-desolvation and interfacial charge transfer:

1 $${\text{Anode stripping}}:\;{\mathrm{Zn}} \to {\mathrm{Zn}}^{2 + } + 2{\mathrm{e}}^{ - }$$

2 $${\text{Bulk hydration equilibrium}}:\;{\mathrm{Zn}}^{2 + } + 6{\mathrm{H}}_{2} {\mathrm{O}} \leftrightarrow \left[ {{\mathrm{Zn}}({\mathrm{H}}_{2} {\mathrm{O}})_{6} } \right]^{2 + }$$

3 $${\text{Interfacial desolvation}}:\;\left[ {{\mathrm{Zn}}({\mathrm{H}}_{2} {\mathrm{O}})_{6} } \right]^{2 + } \to {\mathrm{Zn}}^{2 + } + 6{\mathrm{H}}_{2} {\mathrm{O}}$$

The free-energy penalty of desolvation (ΔG_desolv_):

4 $$\Delta {\mathrm{G}}_{{{\mathrm{desolv}}}} = \Delta {\mathrm{H}}_{{{\mathrm{desolv}}}} - {\mathrm{T}}\Delta {\mathrm{S}}_{{{\mathrm{desolv}}}}$$

where ΔH_desolv_ represents the energy required to disrupt Zn–O coordination and ΔS_desolv_ reflects the entropy gain upon water release. A lower ΔG_desolv_ facilitates interfacial charge transfer and accelerates reaction kinetics [70, 71]. Consequently, the solvation structure of Zn^2+^ directly influences the transport behavior of ions and the reaction kinetics at electrode interfaces.

However, this fundamental Zn^2+^ storage process is inevitably accompanied by side reactions. According to the electrochemical-potential framework (proposed by Goodenough and Kim), the actual electrochemical working window should be located between the lowest unoccupied molecular orbital (LUMO) and highest occupied molecular orbital (HOMO) of electrolyte (Fig. 2b, c) [68, 72]. When the anode potential approaches the LUMO of the electrolyte, electrons are captured by water molecules or protons, initiating the hydrogen evolution reaction (HER):

HER in acidic media:

5 $$2{\mathrm{H}}^{ + } + 2{\mathrm{e}}^{ - } \to {\mathrm{H}}_{2} \;\;\;\;\;\;\;\;\;\;\;\;\;\;\left( {{\mathrm{E}}_{0} = \, 0{\text{V vs}}.{\text{ SHE}}} \right)$$

HER in neutral/alkaline media:

6 $$2{\mathrm{H}}_{2} {\mathrm{O}} + 2{\mathrm{e}}^{ - } \to {\mathrm{H}}_{2} + 2{\mathrm{OH}}^{ - } \;\;\;\;\;\;\;\;\;\left( {{\mathrm{E}}_{0} = \, - 0.{\text{828V vs}}.{\text{ SHE}}} \right)$$

Conversely, when the cathode potential exceeds the HOMO of electrolyte, the oxygen evolution reaction (OER) occurs:

7 $$2{\mathrm{H}}_{2} {\mathrm{O}} \to {\mathrm{O}}_{2} + 4{\mathrm{H}}^{ + } + 4{\mathrm{e}}^{ - } \;\;\;\;\;\;\;\;\;\;\;\left( {{\mathrm{E}}_{0} = { 1}.{\text{23V vs}}.{\text{ SHE}}} \right)$$

Unlike organic battery systems, aqueous electrolytes generally fail to form dense and electronically insulating interphases that can effectively block electron transfer. As indicated by the Pourbaix diagram of Zn (Fig. 2d), metallic Zn is thermodynamically unstable in acidic media and does not readily form a protective passivation layer under near-neutral conditions [35]. Consequently, the reduction in water continuously generates OH^−^ and increases the local pH at the electrode surface:

8 $${\mathrm{Zn}}^{2 + } + {\mathrm{OH}}^{ - } \leftrightarrow {\mathrm{Zn}}({\mathrm{OH}})^{ - }$$

9 $${\mathrm{Zn}} + 2{\mathrm{H}}^{ + } \to {\mathrm{Zn}}^{2 + } + {\mathrm{H}}_{2}$$

The accumulation of OH^−^ and persistent water activity promotes the precipitation of insulating alkaline salts such as Zn_4_SO_4_(OH)_6_·xH_2_O. These deposits increase nucleation overpotential, distort ion flux and induce nonuniform Zn deposition [24]. In turn, electric-field amplification near surface protrusions accelerates dendritic growth and side reactions, eventually leading to short-circuit risks and the formation of electrochemically inactive “dead Zn” [24, 73]. Importantly, these degradation processes are strongly coupled rather than independent. Dendrite growth increases surface roughness and local electric-field heterogeneity, which further promotes uneven Zn deposition. Meanwhile, HER and corrosion modify the interfacial chemical environment by increasing local alkalinity and generating gas, thereby accelerating by-product formation and interfacial passivation. These insulating deposits hinder ion transport and aggravate current inhomogeneity, which in turn further intensifies dendrite growth. Therefore, the instability of liquid electrolytes arises from a self-reinforcing degradation loop involving Zn^2+^ solvation/desolvation chemistry, water-induced parasitic reactions and interfacial transport heterogeneity. These coupled processes are further amplified under temperature variations, making it difficult to simultaneously stabilize Zn deposition, maintain ion transport and suppress side reactions in purely aqueous environments [74]. To address these challenges, HPEs have been proposed as an alternative electrolyte medium. By introducing polymer networks into aqueous systems, HPEs can confine water molecules, regulate Zn^2+^ coordination environments and modulate ion transport near electrode surfaces. Such structural and chemical regulation fundamentally reshapes the physicochemical environment of the electrode–electrolyte interface.

### Anode–Electrolyte Interface in Hydrogel Media

**Fig. 3:**
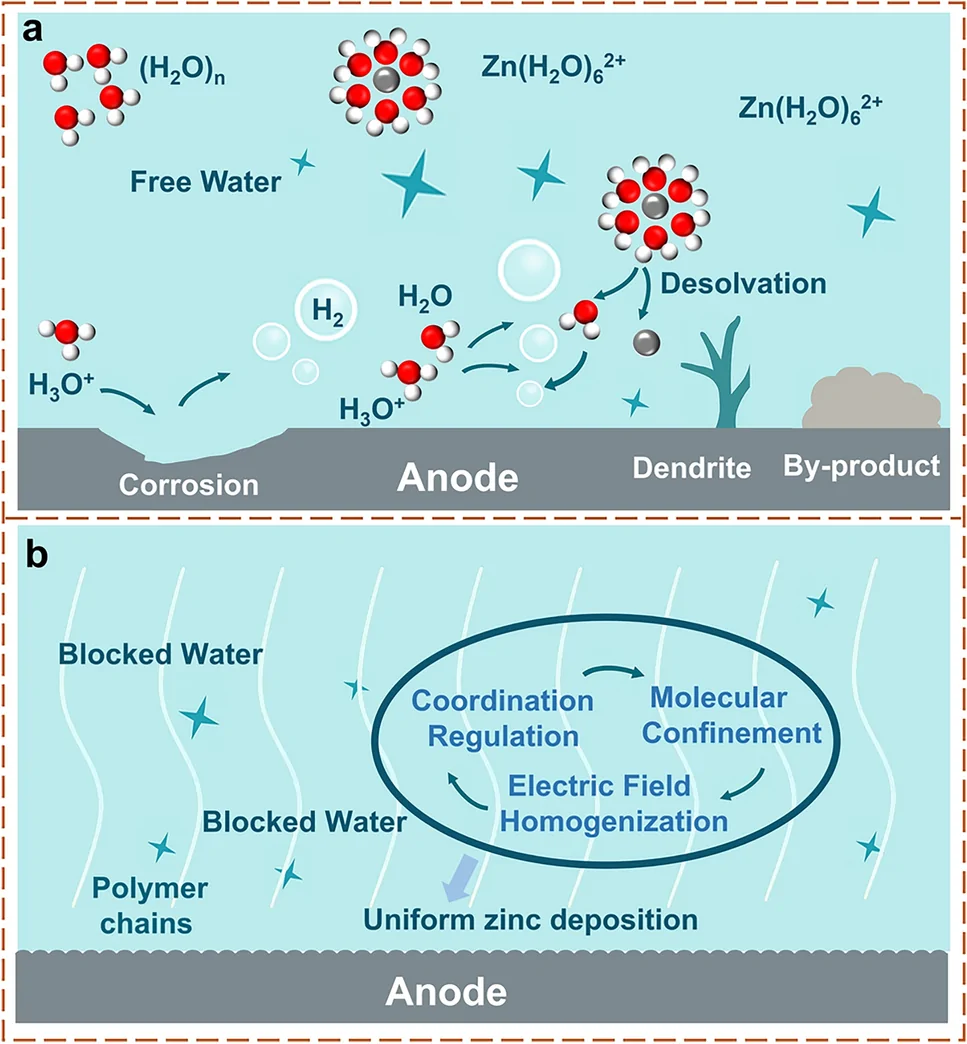
Schematic illustration of side reactions at electrolyte/anode interfaces in AZIBs. **a** Aqueous electrolytes. **b** HPEs

The transition from liquid electrolytes to hydrogel HPEs fundamentally alters the physicochemical environment of AEI [75]. In conventional aqueous electrolytes, the Zn surface is directly exposed to highly active water molecules and strongly hydrated Zn^2+^ species, which promote hydrogen evolution, basic-salt precipitation and dendritic growth, as illustrated in Fig. 3a [16].

By introducing a three-dimensional polymer network into the electrolyte, hydrogel electrolytes establish a confined and chemically regulated interfacial environment. As schematically illustrated in Fig. 3b, the polymer framework modifies the local solvation structure and consequently influences ion-transport behavior near the electrode surface. Through the synergistic effects of water confinement, coordination modulation and ion-flux homogenization, hydrogels alter the key processes governing Zn deposition and interfacial stability [33, 69]. A defining feature of hydrogel electrolytes is the molecular confinement of water. Within the polymer network, a substantial fraction of free water is transformed into bound water associated with polymer chains or hydrogen-bonded clusters. This spatial confinement disrupts the continuous hydrogen-bond network characteristic of bulk water and lowers the activity of reducible protons. Consequently, water participation in parasitic reactions is significantly suppressed, mitigating hydrogen evolution and the formation of alkaline by-products such as Zn_4_SO_4_(OH)_6_·xH_2_O. Meanwhile, restricted water mobility moderates local pH fluctuations near the electrode surface, contributing to a more stable chemical environment at the AEI [48]. Beyond regulating water structure, hydrogel matrices also influence the solvation configuration of Zn^2+^ through coordination interactions with functional groups in the polymer network. Oxygen- or nitrogen-containing groups such as –COOH, –CONH_2_, –OH and –SO_3_^−^ can partially participate in the coordination shell of Zn^2+^ together with water molecules [76–79]. This interaction produces a mixed solvation environment consisting of Zn–O (HPE) and Zn–O (H_2_O) coordination. Such coordination modulation weakens the hydration strength of Zn^2+^ and lowers the desolvation barrier at the electrode surface. As a result, Zn^2+^ transfer across the AEI becomes energetically more favorable, facilitating continuous ion migration and reducing polarization during Zn plating and stripping processes [62, 80]. In addition to molecular-scale regulation, the polymer framework also affects ion transport and electric-field distribution near the electrode surface [68]. The interconnected topology and polarity distribution of hydrogel networks guide ion migration and homogenize ionic flux, alleviating local electric-field amplification at surface protrusions [81]. When appropriate wettability and interfacial adhesion are achieved, the hydrogel forms a mechanically compliant and ionically continuous interface with the Zn metal. Such an interface promotes uniform Zn nucleation and deposition, which is typically reflected by reduced nucleation overpotential, lower interfacial resistance and improved cycling stability [82].

Nevertheless, the stability of AEI strongly depends on the degree of interfacial coupling between the hydrogel electrolyte and the Zn surface [50]. Excessive swelling, polarity mismatch, or insufficient adhesion may induce local current concentration and uneven Zn deposition [30]. Therefore, it is necessary to coordinate the regulation of water activity, Zn^2+^ solvation chemistry and ion-transport uniformity to achieve a robust AEI, rather than optimizing a single parameter. Such coordinated regulation ultimately governs the composition and structure of the AEI, enabling its evolution from loose, by-product-dominated interphases in conventional aqueous electrolytes to more compact and chemically stabilized interphases in hydrogel systems [83]. Extending this concept, interfacial regulation can further go beyond single-electrode optimization. For instance, a Janus biopolymer separator enables dual-interfacial regulation by simultaneously modulating Zn deposition at the anode and suppressing polyiodide shuttling at the cathode, illustrating the feasibility of coupled interfacial design in advanced zinc battery systems [84].

### Cathode–Electrolyte Interface in Hydrogel Media

**Fig. 4:**
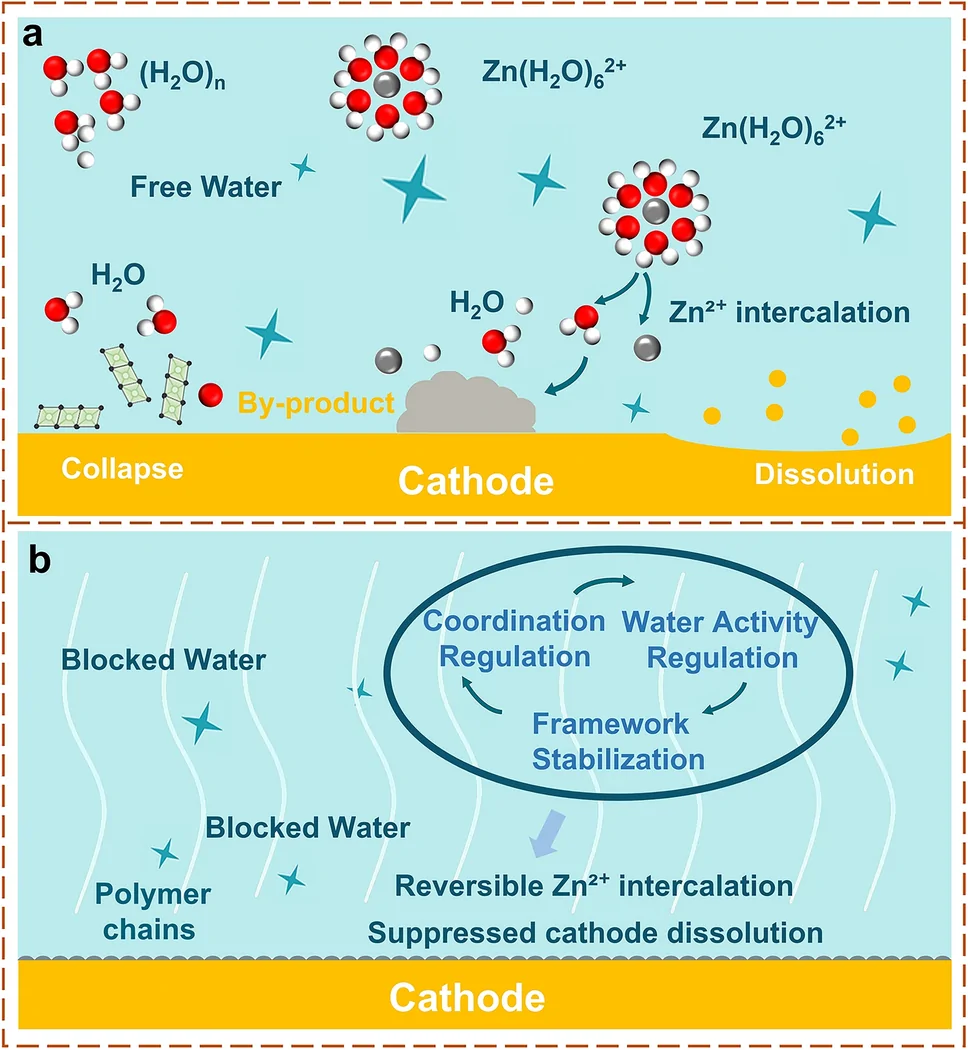
Schematic illustration of side reactions at electrolyte/cathode interfaces in AZIBs. **a** Aqueous electrolytes. **b** HPEs

While the AEI governs the reversibility of Zn plating and stripping, the CEI is equally critical in determining the structural stability, ion-storage reversibility and long-term cycling durability of AZIBs [85]. In aqueous electrolytes, cathode degradation is closely associated with instability of the local electrolyte environment. In particular, excess free water and the relatively narrow electrochemical stability window of aqueous systems can trigger dissolution, phase transformation and progressive structural degradation of active materials (Fig. 4a) [86]. Compared with the AEI, which mainly operates under reductive conditions, the CEI is exposed to oxidative potentials, transition-metal redox reactions and, in some systems, anion participation, resulting in more complicated degradation pathways [87]. In conventional liquid electrolytes, cathode degradation generally originates from the coupling of chemical reactions, structural evolution and interfacial side reactions. For transition-metal oxide cathodes such as manganese- and vanadium-based compounds, repeated Zn^2+^ and H^+^ insertion may induce lattice distortion, amorphization and dissolution of metal species [88]. In Mn-based cathodes, free-water-induced side reactions and unstable local environments accelerate Mn^3+^ disproportionation, Jahn–Teller distortion and structural collapse. In vanadium-based cathodes, hydrolysis and dissolution of vanadium species can further trigger hydrolysis-reprecipitation processes and the formation of electrochemically inactive surface products. Meanwhile, hydrolysis reactions and local pH fluctuations promote the formation and accumulation of basic zinc salts on the electrode surface, which block ion-diffusion pathways and increase polarization [36, 37, 89]. Prussian blue analogues (PBAs) may suffer from vacancy growth, lattice-water loss and framework fracture, whereas organic and conductive-polymer cathodes can undergo overoxidation, dopant loss and dissolution of redox-active species [90]. For small organic cathodes, electrolyte-induced dissolution may also aggravate self-discharge and poor cycling retention [91]. These degradation pathways collectively disrupt both electron and ion transport, leading to progressive structural deterioration and capacity fading [92, 93].

The introduction of HPEs alters the cathode interfacial environment primarily through regulation of water activity, ion transport and interfacial contact. Within the polymer network, the mobility and reactivity of water molecules are partially restricted, which reduces direct solvent attack on cathode materials and suppresses dissolution of transition-metal species. More importantly, the conversion of part of the free water into bound or confined water changes the local hydrogen-bonding environment at the cathode side, thereby mitigating water-induced structural erosion and parasitic interfacial reactions, as illustrated in Fig. 4b [94, 95]. In addition, the modified solvation environment of Zn^2+^ in hydrogels can lower the desolvation barrier at the cathode surface and improve the reversibility of Zn^2+^ insertion/extraction. For hydrolysis-sensitive vanadium cathodes, reduced water activity suppresses hydrolysis-reprecipitation reactions and helps maintain open diffusion channels for Zn^2+^ insertion [76, 94]. In PBAs, controlled hydration environments stabilize [Fe(CN)_6_] coordination frameworks, suppress vacancy formation and sustain fast ion transport [96]. For organic and layered hybrid cathodes, hydrogen-bonded hydrogel matrices inhibit molecular dissolution and enhance interfacial contact, improving both electronic and ionic pathways [97, 98].

Beyond molecular confinement, the unique Zn^2+^ solvation structure in hydrogels also modifies the thermodynamics of CEI reactions. Partial replacement of Zn^2+^–H_2_O coordination by oxygen- or nitrogen-containing donor groups from polymer chains weakens the solvation enthalpy and facilitates Zn^2+^ desolvation at the cathode interface [65]. This not only lowers the energy barrier for Zn^2+^ intercalation but also suppresses oxidative parasitic reactions, including OER, at elevated cathode potentials. Meanwhile, reduced water activity broadens the effective electrochemical stability window, enabling stable cathode operation at higher potentials without severe oxidative decomposition of the electrolyte [86, 99]. These effects collectively stabilize cathode redox reactions, reduce polarization and improve CEI reaction kinetics. Beyond these thermodynamic and kinetic effects, the mechanical compliance and polarity distribution of hydrogel networks further contribute to cathode interfacial stability. The elastic polymer framework can buffer lattice strain, homogenize local reaction fields and accommodate volume fluctuations as well as Jahn–Teller distortions during repeated Zn^2+^ insertion/extraction, thereby preventing crack propagation and interfacial delamination [82]. Meanwhile, the more uniform ionic flux promoted by the hydrogel matrix facilitates the formation of compact and stable cathode interphases, suppresses phase segregation and maintains high reversibility during prolonged cycling [30, 87].

Such interfacial stabilization mechanisms have been increasingly substantiated by recent experimental studies. For example, Zhao et al. constructed an electrolyte-triggered hydrogel interphase on MnO_2_ cathodes, in which in situ gelation during electrolyte infiltration and cycling forms a continuous interfacial layer [100]. This hydrogel interphase physically separates the cathode surface from highly reactive water and improves electrode–electrolyte contact, thereby suppressing Mn dissolution and sulfate-derived by-product accumulation. More direct evidence of chemically evolved CEI formation was provided by Gu et al. in a cetyltrimethylammonium bromide (CTAB)-based micellar electrolyte extended to a polyacrylamide (PAM) quasi-solid-state zinc-vanadium battery system [101]. Interfacial reactions involving CTA^+^ (the cationic headgroup derived from the surfactant cetyltrimethylammonium bromide) and trifluoromethanesulfonate (OTF)-derived species induce the formation of an organic–inorganic hybrid CEI. Depth-profiled X-ray photoelectron spectroscopy (XPS) and high-resolution transmission electron microscopy (HRTEM) analyses reveal that this interphase comprises organic components (e.g., C-N/R_4_N^+^ species) together with inorganic species such as ZnF_2_, ZnS, carbonates and Br-containing compounds, forming a uniform amorphous-nanocrystalline protective layer on the cathode surface. This chemically evolved CEI effectively suppresses vanadium dissolution, while stabilizing local pH fluctuations and mitigating direct water attack without compromising Zn^2+^ transport kinetics.

As a result, these studies indicate that cathode stabilization in hydrogel systems arises from both bulk electrolyte regulation and cathode-side interphase formation with interfacial chemical evolution. However, compared with the extensively studied anode interface, direct experimental evidence regarding CEI chemistry, interfacial structural evolution and operando cathode-side reaction pathways in hydrogel electrolytes remains limited. In particular, the dynamic evolution of CEI composition, local pH microenvironments and Zn^2+^ transport behavior under wide-temperature conditions remains poorly understood. Accordingly, cathode interfacial regulation in hydrogel electrolytes remains underexplored and warrants further investigation, requiring multiscale studies and advanced operando characterization.

### Opportunities and Challenges of HPEs

From the perspective of electrolyte-state regulation, HPEs occupy a unique yet highly tunable intermediate regime between conventional liquid electrolytes and solid-state electrolytes. Liquid-state electrolytes generally provide the highest ionic conductivity and low interfacial resistance, but they are prone to leakage, uncontrolled parasitic reactions and dendrite growth. By contrast, solid-state electrolytes offer superior mechanical strength and improved safety, yet often suffer from limited ionic conductivity, rigid interfacial contact and large charge-transfer resistance [102, 103]. Owing to their three-dimensional polymer networks, HPEs integrate liquid-like ionic conductivity with solid-like structural confinement, thereby enabling a more balanced yet inherently constrained regulation of water activity, Zn^2+^ solvation environments, interfacial compatibility and mechanical adaptability. This intermediate-state characteristic renders HPEs particularly attractive for wide-temperature AZIBs, while simultaneously introducing inherent trade-offs among ion transport, structural robustness and interfacial stability.

A wide range of polymer matrices have been developed for constructing hydrogel electrolytes, and representative systems together with their key physicochemical properties are summarized in Table 1. In general, hydrophilic functional groups within polymer networks, such as hydroxyl, amide and carboxyl groups, interact with Zn^2+^ ions and water molecules through coordination or hydrogen bonding [16, 29]. These interactions regulate the solvation and desolvation behavior of Zn^2+^, reduce excessive water activity and stabilize both the AEI and CEI. Meanwhile, the structural tunability of polymer matrices enables the integration of additional functionalities, including antifreezing capability, flame retardancy, self-healing behavior and ion-selective transport. Such multifunctionality expands the applicability of HPEs in flexible electronics and environmentally adaptive energy storage systems [73].

**Table 1:** Representative polymer matrices and key properties in HPEs for AZIBs

Polymer (Formula)	Ionic conductivity [S cm^−1^]	Water content [%]	Mechanical properties	Advantages	Limitations	Ref
PVA (C_2_H_4_O)_n_	~ 10^–3^–10^–2^	60–80	Moderate tensile strength, high elongation	Rich hydroxyl groups, easy gelation, self-healing	Limited salt tolerance	[51, 52, 104, 105]
PAM (C_3_H_5_NO)_n_	~ 10^–3^–10^–2^	50–90	High tensile strength, excellent flexibility	Abundant amide groups, tunable mechanics	Average conductivity	[53, 58, 106, 107]
PAA (C_3_H_4_O_2_)_n_	~ 10^–4^	80–95	Moderate strength and elongation	Strong Zn^2+^ coordination, dendrite suppression	Excessive swelling, limited durability	[108, 109]
PEG (C_2_H_4_O)_n_	~ 10^–5^–10^–3^	35–70	Good flexibility, moderate modulus	Excellent salt dissolving ability	High crystallinity at RT, poor adhesion	[67, 110, 111]
SA [(C_6_H_7_O_6_Na)_n_]	~ 10^–4^–10^–3^	80–95	Low strength, moderate flexibility	Natural, biodegradable, cross-links with Zn^2+^	Swelling, weak mechanical integrity	[112]
CS [(C_6_H_11_NO_4_)_n_]	~ 10^–4^	60–85	Moderate strength, poor flexibility	Antibacterial, biocompatible, metal-ion coordination	Poor solubility, pH-sensitive, low σ	[113–115]
Gelatin (polypeptide)	~ 10^–4^	70–90	Soft, elastic, low modulus	Biocompatible, tunable water uptake	Poor thermal stability	[116–118]
Cellulose [(C_6_H_10_O_5_)_n_]	~ 10^–4^-10^–3^	60—90	High modulus, flexible after hydration	Renewable, strong H-bonding, good mechanical stability	Poor solubility, processing difficulty	[57, 87, 102]

Actual performance varies with polymer cross-linking, Zn-salt concentration, water activity and measurement conditions

**Fig. 5:**
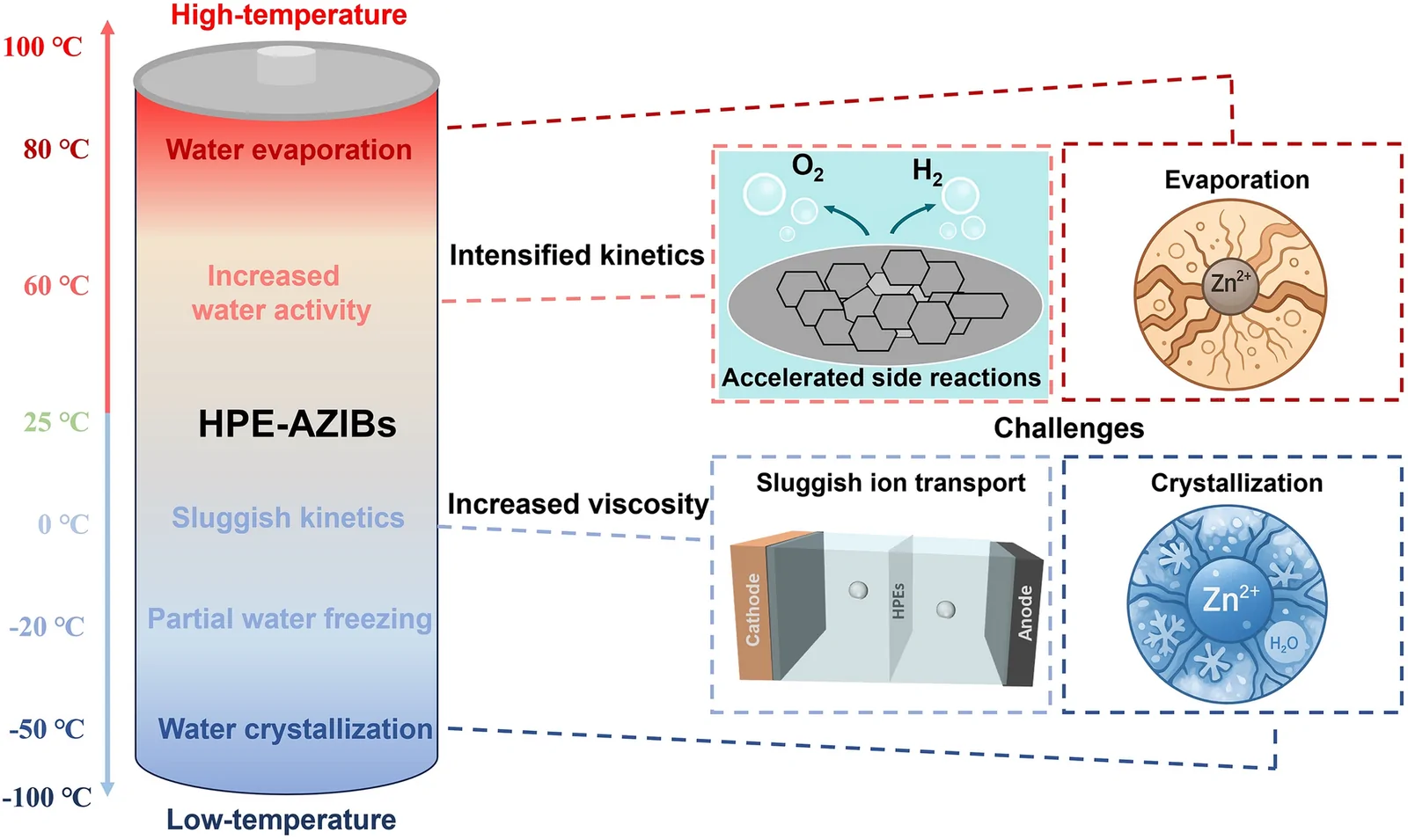
Schematic illustration of low- and high-temperature limitations of HPEs in AZIBs

Despite these advantages, the multifunctional nature of HPEs inevitably introduces inherent trade-offs among ionic transport, structural robustness and interfacial compatibility. Increasing the cross-linking density of polymer networks can enhance mechanical strength and dimensional stability, but it also restricts polymer-chain segmental motion and reduces ionic conductivity [119]. Conversely, increasing water content or reducing cross-link density can improve ion mobility, yet often compromises mechanical integrity and accelerates water loss or swelling. These competing effects indicate that structural optimization of polymer networks alone is insufficient to simultaneously achieve high ionic conductivity, robust mechanical properties and stable electrode/electrolyte interfaces. Under wide-temperature conditions, these limitations become substantially amplified because the physicochemical properties of HPEs evolve dynamically with temperature (Fig. 5). At low temperatures, the hydrogen-bond network of water becomes increasingly ordered and free water or weakly bound water may partially freeze. This process increases electrolyte viscosity, suppresses polymer-chain dynamics and strengthens Zn^2+^–H_2_O coordination, collectively raising the desolvation barrier and slowing ion transport. Simultaneously, freezing-induced contraction of the hydrogel network weakens interfacial contact and increases interfacial resistance. The ordered hydrogen-bond network, partial water freezing and weakened interfacial contact hinder Zn^2+^ transport and interfacial charge transfer, leading to increased charge-transfer resistance and voltage hysteresis during Zn plating/stripping. Consequently, the cells exhibit enhanced polarization, reduced reversible capacity, capacity retention decay and shortened cycle life under subzero conditions [52, 105]. At elevated temperatures, accelerated water evaporation disrupts the hydrogen-bond equilibrium within the polymer-water network. The resulting dehydration and structural shrinkage alter pore structures, introduce microstructural defects and increase local salt concentration, collectively hindering ion transport and destabilizing electrode interfaces. In addition, higher water activity promotes parasitic reactions such as hydrogen evolution and corrosion, which continuously consume active Zn and electrolyte components and further aggravate interfacial degradation. Consequently, the cells suffer from decreased Coulombic efficiency, unstable cycling behavior and degradation of energy and power output at elevated temperatures [100].

Therefore, the wide-temperature challenge of HPEs lies not merely in maintaining electrolyte-state stability, but more fundamentally in achieving the simultaneous optimization of ion transport, interfacial reversibility and structural integrity under dynamically evolving thermodynamic and kinetic conditions [78]. Although various regulation strategies for wide-temperature HPEs have been explored, including polymer system regulation, salt system regulation, cosolvent regulation and filler regulation, these approaches remain largely separated in terms of their underlying physicochemical mechanisms and lack a unified cross-scale design framework. As a result, Zn^2+^ solvation behavior, ion transport and interfacial processes are often regulated independently rather than synergistically integrated, thereby limiting stable electrochemical performance across wide-temperature ranges [120, 121].

This limitation is closely associated with the intrinsic interplay among multiscale processes within the system, including water-structure evolution, Zn^2+^ coordination reconstruction, polymer-network response and interfacial reactions. The coupling among these factors makes it difficult for individually optimized strategies to deliver consistent performance at the system level. Consequently, the key issue is not the absence of effective approaches, but the lack of a unified framework capable of linking local coordination chemistry with multiscale structural adaptability and interfacial processes in a consistent manner. Based on this understanding, the following section introduces the C-E regulation framework. This framework provides a unified perspective that links solvation chemistry, multiscale structural regulation and interfacial processes, enabling a coherent design approach for optimizing ion transport, interfacial stability and structural integrity across a wide-temperature range.

## Unified Regulation Principles of the C-E Framework

**Fig. 6:**
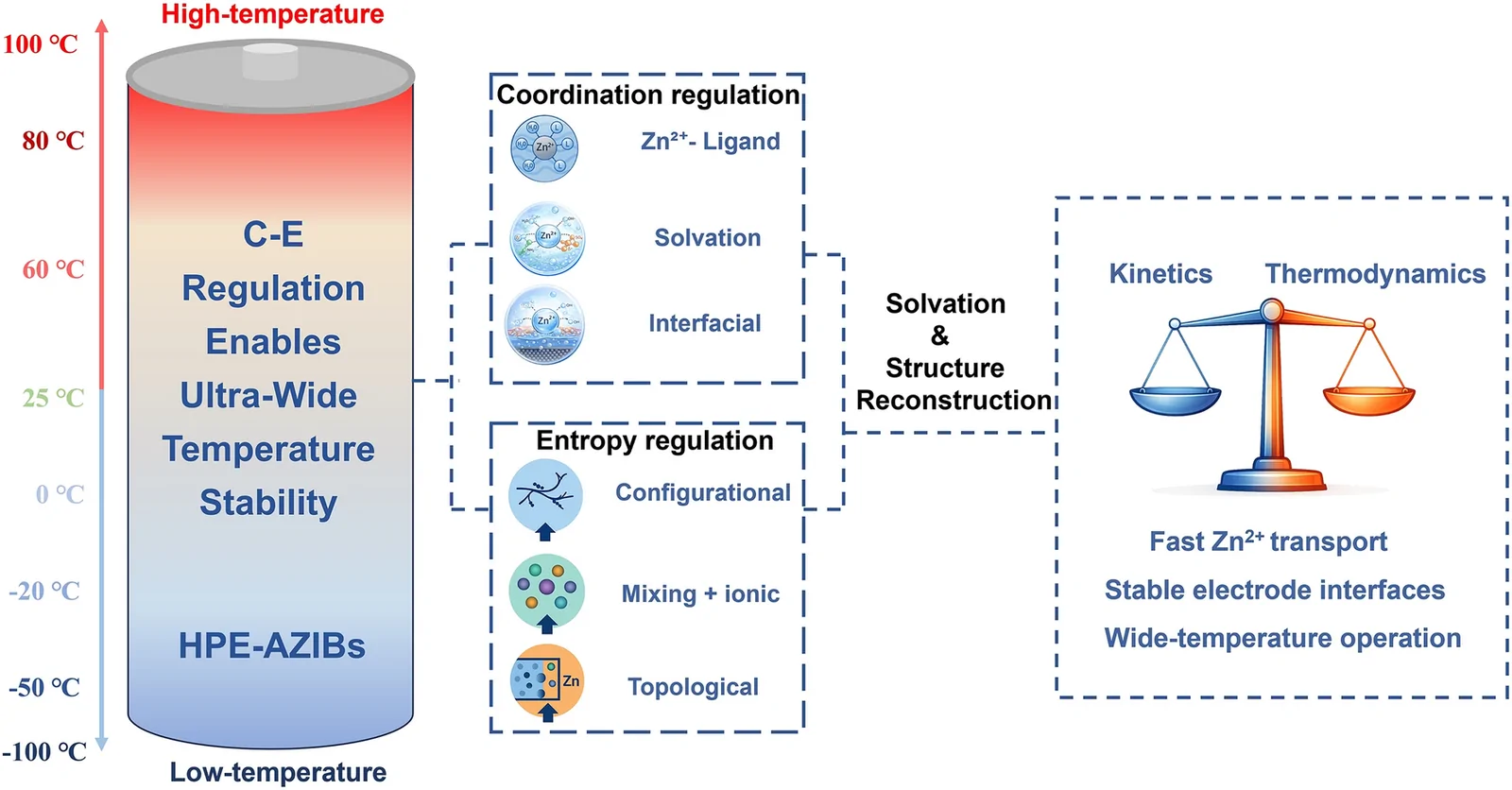
Schematic illustration of the C-E regulation framework as a unifying cross-scale design perspective for HPEs in wide-temperature AZIBs

The electrochemical behavior of HPEs under wide-temperature conditions is governed by coupled physicochemical processes rather than by a single limiting factor. Ion solvation, hydrogen-bond organization, ion transport and interfacial reactions dynamically interact across multiple structural scales, collectively determining the reversibility and stability of Zn electrochemistry under practical operating conditions [122]. Consequently, effective wide-temperature electrolyte design requires simultaneous regulation of local coordination environments, transport-accessible structural states and ion-transport adaptability. To address these coupled limitations, the C-E regulation framework is proposed as a unified cross-scale regulation principle for HPE systems, as illustrated in Fig. 6. Compared with conventional strategy-oriented approaches, such as solvation engineering or polymer-network optimization, the C-E framework provides a more general physicochemical perspective for understanding how coordination interactions and multiscale structural regulation collectively determine electrolyte behavior under broad operating temperatures.

Within the C-E regulation framework, coordination regulation primarily governs local interaction energetics through modulation of Zn^2+^ coordination environments, whereas entropy regulation governs the distribution and accessibility of structural states across multiple scales. Rather than operating independently, these two regulation mechanisms remain dynamically coupled throughout electrochemical operation. Coordination regulation stabilizes local solvation structures and interfacial reaction pathways, while entropy regulation maintains structural adaptability and transport accessibility under dynamically changing conditions [121]. Their synergistic coupling further links molecular-scale solvation chemistry with multiscale thermodynamic stability, ion-transport kinetics and interfacial electrochemical behavior. Based on these principles, the following sections further discuss the respective roles of coordination regulation and entropy regulation, as well as their synergistic effects on the thermodynamic behavior, kinetic processes, temperature-dependent regulation priorities and practical electrolyte design principles of HPE systems.

### Regulation of Molecular Coordination Environment

Since Werner first established coordination theory, donor–acceptor interactions between metal ions and ligands have served as a fundamental concept in modern chemistry [123, 124]. In AZIBs, coordination regulation provides an effective molecular-level strategy to regulate the Zn^2+^ solvation environment, thereby affecting the energetics of interfacial electrochemical reactions. Within the proposed C-E framework, coordination regulation represents the molecular-level design lever that governs the local interactions among Zn^2+^, water molecules, anions and polymer functional groups, thereby reconstructing the primary solvation structure of Zn^2+^ and modulating the thermodynamic and kinetic characteristics of interfacial processes [125]. In conventional aqueous electrolytes, Zn^2+^ predominantly exists as the octahedral complex [Zn(H_2_O)_6_]^2+^. This strongly hydrated structure is thermodynamically stable but kinetically unfavorable for electrochemical reactions because the strong Zn–O (H_2_O) interactions impose a large energetic penalty for desolvation (ΔH_desolv_). As a result, interfacial charge transfer is limited by the removal of coordinated water, which increases polarization and accelerates parasitic reactions such as hydrogen evolution and by-product formation [61]. Coordination regulation targets this fundamental limitation by modifying the local ligand environment of Zn^2+^ and weakening the dominance of water in the primary solvation shell [64].

**Fig. 7:**
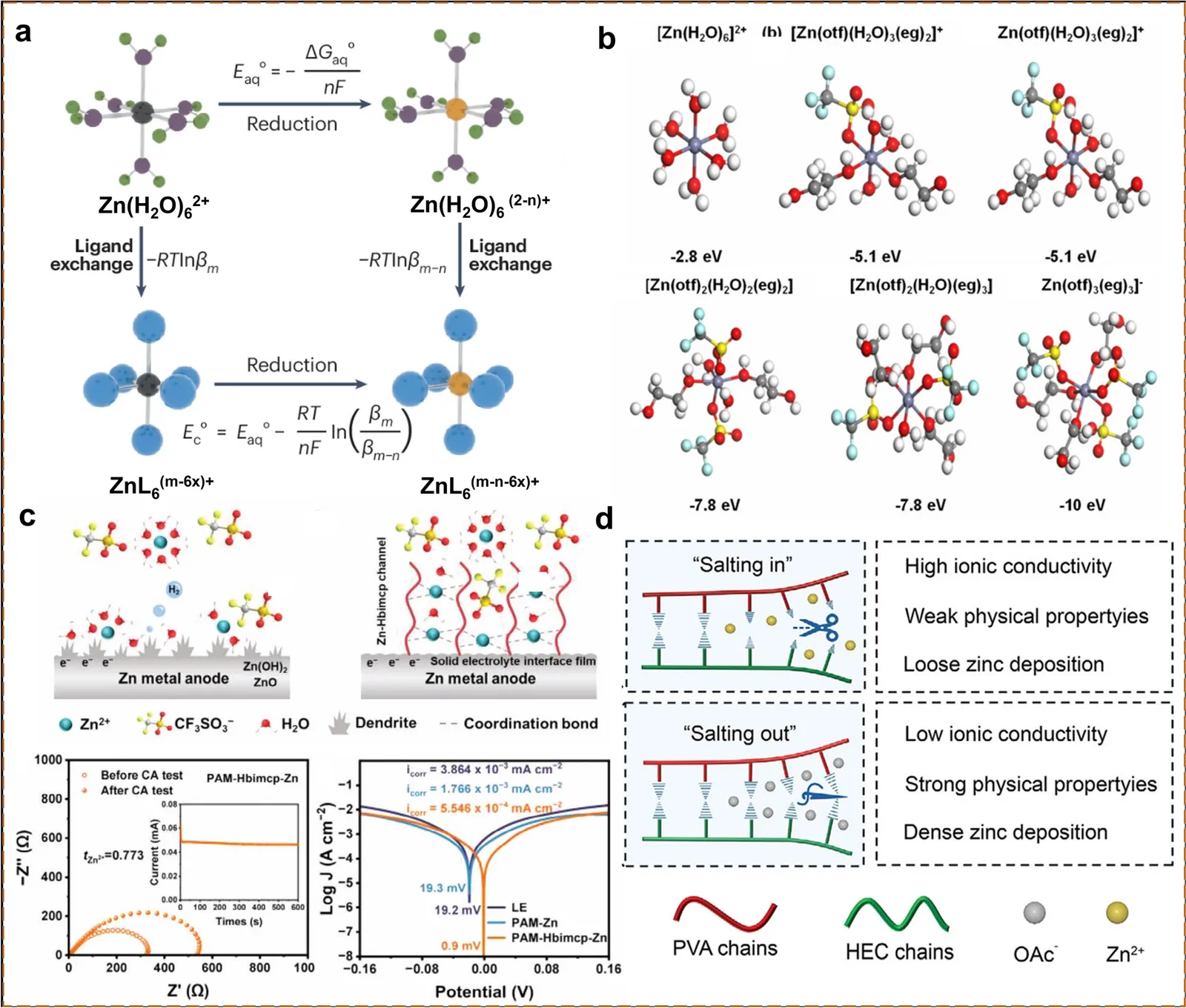
Coordination regulation of Zn^2+^ solvation and interfacial processes in HPEs. **a** Correlation between redox potential and stability constants of Zn–ligand complexes [78]. Copyright 2025, Springer Nature. **b** Zn^2+^ solvation/desolvation behavior and associated interfacial charge-transfer kinetics [130]. Copyright 2024, Royal Society of Chemistry. **c** Enhanced Zn^2+^ transport and suppressed dendrite growth in HPEs enabled by coordination regulation [134]. Copyright 2024, Wiley. **d** Concentration-gradient hydrogel electrolyte integrating salting-in and salting-out effects [135]. Copyright 2026, Wiley

HPEs introduce abundant coordination sites through functional groups on polymer chains, enabling deliberate regulation of Zn^2+^ solvation structures. Functional groups such as –COOH, –CONH_2_, –OH and –SO_3_H can partially replace water molecules in the primary solvation shell to form Zn-ligand inner-sphere complexes [126, 127]. This ligand substitution weakens strong Zn–O (H_2_O) interactions and lowers the energetic cost associated with desolvation. In addition, multidentate ligands can stabilize more compact coordination configurations and accelerate ligand-exchange dynamics within the solvation sheath, thereby facilitating Zn^2+^ migration between adjacent coordination environments [128, 129]. This coordination-induced reconstruction of the primary solvation structure is schematically illustrated in Fig. 7a, where the partial replacement of coordinated water by polymer ligands or anions provides a more energetically accessible pathway for interfacial reactions. Coordination regulation also influences the thermodynamic characteristics of Zn-based electrochemical reactions. The redox potential of a metal complex depends on the relative stability of its oxidized and reduced coordination states. When the oxidized complex is stabilized relative to the reduced species (β_m_/β_m-n_ > 1), the redox potential shifts negatively, whereas stabilization of the reduced state causes a positive shift. Therefore, tuning ligand identity and coordination strength can alter the interfacial potential landscape, reduce polarization and improve reaction reversibility [78]. For example, He et al. designed an ethylene glycol/zinc triflate deep-eutectic electrolyte and showed that the balance between water and coordinating species governs the evolution of Zn^2+^ solvation structures, as revealed by density functional theory calculations (Fig. 7b) [130]. By regulating this coordination environment, the electrolyte achieved improved reversibility of Zn plating/stripping together with stable cathodic reactions. From the perspective of electronic structure, crystal-field theory (CFT) and ligand-field theory (LFT) provide a conceptual basis for understanding how ligand environments influence the surface energetics of metal centers [78]. Although the crystal-field splitting of Zn^2+^ (3d^10^) is relatively small, variations in ligand-field strength can still influence interfacial polarization and reaction energetics, and analogous coordination principles are broadly relevant to transition-metal centers in battery cathodes [131–133].

Beyond static coordination environments, hydrogel systems also enable dynamic coordination motifs that further regulate Zn^2+^ transport and interfacial processes. In HPEs, coordination regulation can be further strengthened by dynamic multidentate ligands and polymer-mediated solvation reconstruction. As illustrated in Fig. 7c, supramolecular hydrogel electrolytes featuring reversible Zn-ligand coordination motifs can reconstruct the inner solvation environment of Zn^2+^, promote anion participation in the coordination sheath and generate dynamically exchangeable coordination states during Zn^2+^ migration. Such dynamic coordination extends beyond simple Zn^2+^ binding, providing transient capture-release sites that buffer local concentration fluctuations, homogenize Zn^2+^ flux and facilitate interfacial desolvation and charge transfer [134]. Consequently, the hydrogel electrolyte exhibits lower interfacial resistance and a higher Zn^2+^ transference number, while the more uniform interfacial ion distribution favors compact Zn nucleation and crystallographically regulated lateral growth. These coupled effects effectively suppress dendritic protrusion and improve Zn deposition uniformity and reversibility. Beyond ligand-based coordination motifs, coordination regulation in hydrogels can also emerge from ion-specific polymer–water interactions that reshape local hydration environments. As illustrated in Fig. 7d, salt concentration gradients reshape hydrogen-bond networks, redistribute free and bound water and alter local ion accessibility across the hydrogel matrix [135]. Rather than simply modifying an individual Zn^2+^ solvation shell, such Hofmeister-type effects create a spatially differentiated interfacial environment, in which the salt-rich, mechanically dense region near the Zn anode suppresses excessive water activity and stabilizes deposition, while the relatively hydrated region maintains continuous ionic conduction. Through this coupling of polymer–ion interactions, hydration redistribution and gradient-mediated transport regulation, the interfacial Zn^2+^ flux can be homogenized without sacrificing the structural robustness of the hydrogel network. This example therefore highlights that coordination regulation in HPEs may extend beyond discrete ligand substitution to the broader ion–polymer–water microenvironment that governs interfacial transport and deposition selectivity.

Overall, coordination regulation in HPEs enables molecular-level control over Zn^2+^ solvation environments, desolvation energetics and interfacial reaction pathways. By partially replacing coordinated water with polymer ligands, anions or dynamic coordinating species, the solvation shell of Zn^2+^ can be adjusted to provide a more energetically accessible pathway for interfacial reactions. At the same time, dynamic ligand exchange and ion-specific coordination environments can extend this molecular regulation to ion transport and deposition behavior at the interface. These coordination-induced modifications establish the molecular foundation for the multiscale thermodynamic and kinetic behaviors of Zn electrochemistry discussed in the following sections.

### Regulation of Entropy Across Multiple Scales

Although coordination regulation can effectively tune the local coordination structure of Zn^2+^ and the associated interfacial reaction behavior, it primarily operates at the level of energy-governed local interactions and is insufficient to fully describe the structural distribution characteristics and accessibility of the system under practical operating conditions [136]. In complex multiphase hydrogel systems, different solvation structures, ion-association states and transport pathways typically coexist with distinct populations and undergo redistribution in response to external conditions [121, 137]. On this basis, entropy regulation is introduced to characterize the number and distribution of structural states across multiple length scales. Rather than being simply interpreted as a measure of disorder, entropy regulation originates from representative entropy contributions associated with different structural degrees of freedom in the system, including mixing entropy arising from compositional diversity, ionic entropy associated with ion distribution and association behavior, configurational entropy related to polymer-chain conformations and local structural states, and topological entropy governed by network connectivity and transport-pathway distribution [61]. These entropy contributions collectively determine the multiscale distribution and accessibility of solvation structures, ion transport and interfacial processes. To further clarify the roles of different entropy contributions in structural regulation, the representative entropy types and their structure–property relationships are summarized in Table 2.

**Table 2:** Representative entropy contributions and their structure–property roles in HPEs

Entropy contribution	Main structural degree of freedom	Regulates	Results	Enhances	Ref
Mixing entropy (S_mix_)	Multicomponent composition, including salts, solvents, polymers and additives	Water-solute and polymer–water interactions	Disruption of ordered hydrogen-bond networks and suppressed ice nucleation	Antifreezing capability and low-temperature transport continuity	[137]
Ionic entropy (S_ion_)	Ion-pair distribution and ionic spatial distribution	Ion-association/dissociation behavior and ionic flux	Reduced ion clustering and concentration polarization	Ionic conductivity and homogeneous Zn^2+^ flux	[138]
Configurational entropy (S_conf_)	Polymer-chain conformations and local configurational states	Segmental dynamics and solvation-state diversity	Dynamic solvation environments and reduced desolvation barrier	Zn^2+^ diffusion and interfacial reaction kinetics	[121, 139]
Topological entropy (S_topo_)	Network connectivity and transport-pathway distribution	Ion-channel continuity and pathway multiplicity	Multichannel ion transport and dynamic flux redistribution	Flux uniformity and long-term cycling stability	[140]

On the basis of the representative entropy contributions described above, entropy regulation fundamentally originates from tuning the structural degrees of freedom and the distribution characteristics of accessible states within the system [141]. From the compositional perspective, increasing the diversity of salts, solvents and polymer components can enhance mixing entropy, thereby introducing a broader range of solvation and interaction states [138, 141]. From the perspective of ionic behavior, regulating ion distribution and association behavior can alter the relative populations of different ion-associated states, thereby increasing ionic entropy. In polymer systems, increased diversity in local solvation structures, polymer-chain conformations and local environmental heterogeneity can broaden the accessible configurational-state space and thus enhance configurational entropy. In addition, the construction of multiphase network structures, continuous ion-conduction channels and heterogeneous transport pathways can increase the diversity and connectivity of ion-migration pathways, thereby contributing to higher topological entropy, which can be expressed as Eq. (10):

10 $$S = - R\mathop \sum \limits_{i = 1}^{n} p_{i} {\mathrm{lnp}}_{i}$$

where R is the gas constant and ${p}_{i}$ represents the probability distribution of the i-th structural state. It should be noted that Eq. (10) is not intended to rigorously calculate the total thermodynamic entropy of complex HPE systems, but rather to serve as a statistical descriptor for characterizing the distribution features and relative populations of different structural states. When the number of accessible structural states increases, or when the distribution among different states becomes more uniform, the corresponding entropy contribution of the system increases accordingly. In practical HPE systems, different entropy contributions are generally difficult to quantify independently and are more commonly evaluated through correlated experimental and theoretical analyses associated with specific structural states [142]. For example, mixing entropy is often associated with compositional complexity and freezing behavior and can be indirectly evaluated through differential scanning calorimetry (DSC) measurements and freezing-point variations [137]. Ionic entropy is primarily related to the distribution of ion-associated states and can be analyzed through peak deconvolution of characteristic anion-coordination bands in Raman or Fourier transform infrared (FTIR) spectra [136]. Configurational entropy is mainly associated with local solvation structures and configurational-state distributions and can be analyzed through coordination-number statistics, radial distribution functions and solvation-structure analysis derived from molecular dynamics simulations [121, 139]. Topological entropy primarily reflects network connectivity and ion-transport pathway distributions and can be indirectly evaluated through the correlation between network structural uniformity, multichannel ion-transport behavior and ion-diffusion characteristics [140].

**Fig. 8:**
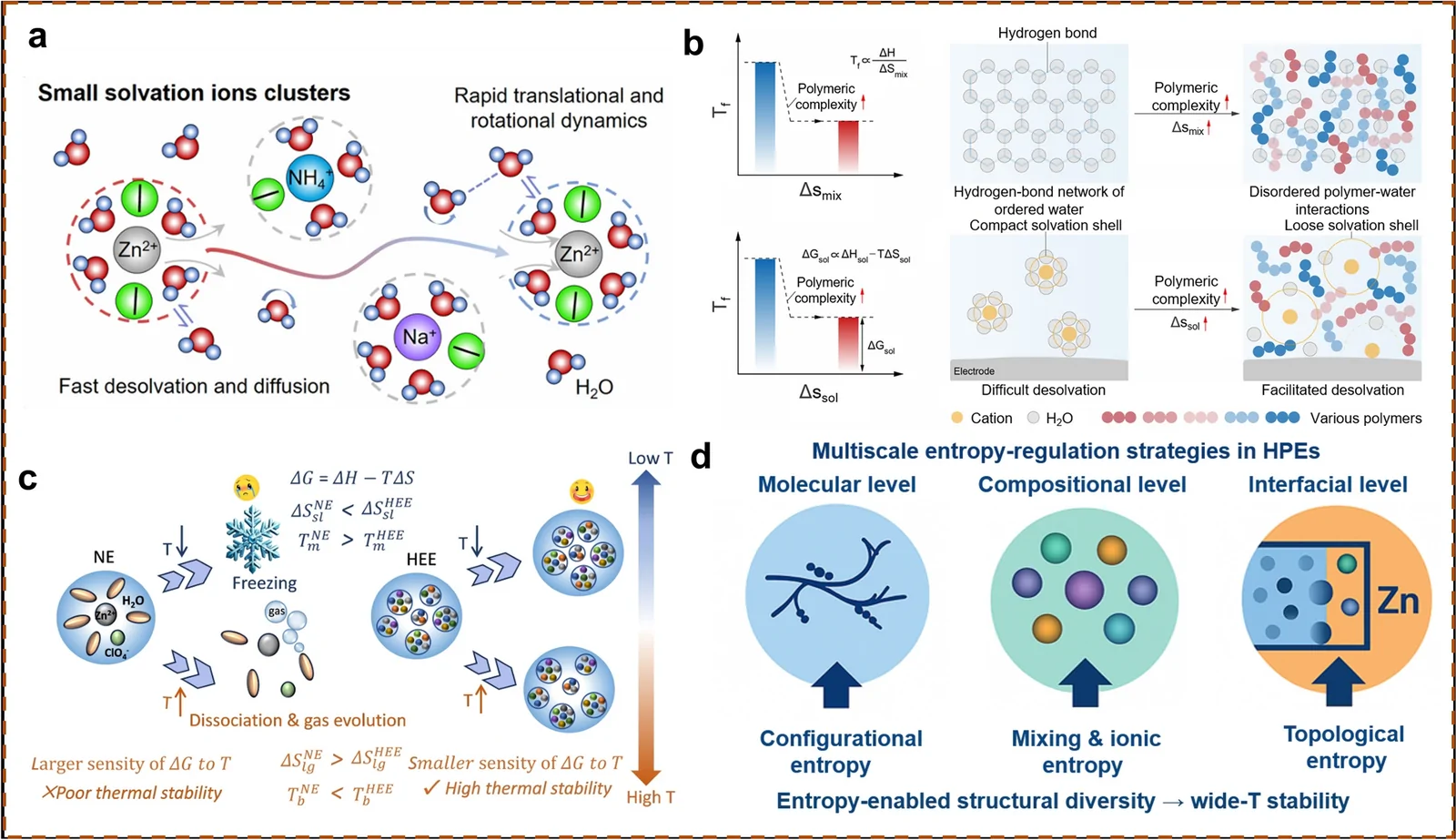
Entropy regulation mechanisms and design strategies for wide-temperature stability in aqueous and polymer electrolytes. **a** Zn^2+^ transport under entropy regulation in low-temperature electrolytes [121]. Copyright 2025, American Chemical Society. **b** High-entropy hydrogel design via polymeric complexity [137]. Copyright 2026, American Chemical Society. **c** Thermodynamic mechanism of entropy regulation for wide-temperature stability [138]. Copyright 2025, Wiley. **d** Multiscale entropy regulation strategies in HPEs

In terms of specific regulation mechanisms, entropy regulation first manifests at the molecular scale through the modulation of water structure and ion-association behavior. Mixing entropy and ionic entropy increase the compositional diversity and ionic distribution uniformity of the system, thereby disrupting ordered hydrogen-bond networks and suppressing ice nucleation. Meanwhile, these entropy contributions weaken ion pairing and the formation of large aggregated solvation clusters, promoting their transition into smaller and dynamically exchangeable structures. This process increases the accessibility of transport-related structural states and thus facilitates Zn^2+^ migration and desolvation under low-temperature conditions. The high-entropy chloride electrolyte reported by Chen et al. represents a typical example of this mechanism. As illustrated in Fig. 8a, increasing salt-component diversity enhances both mixing entropy and ionic entropy, thereby disrupting hydrogen-bond ordering, reducing the size of Zn-Cl-associated solvation clusters and enabling parallel Zn^2+^ transport and desolvation processes at low temperatures [121]. Different from the conventional sequential migration mechanism of large aggregated solvation clusters, this strategy effectively reduces polarization and promotes uniform Zn deposition.

In HPE systems, entropy regulation further extends to configurational entropy, which mainly originates from the diversity of polymer-chain conformations, local solvation structures and local environmental states. Flexible polymer backbones, heterogeneous functional-group distributions and dynamic interaction sites can broaden the accessible configurational-state space. As illustrated in Fig. 8b, increased polymeric complexity enhances the diversity of polymer–water and polymer–ion interaction states, thereby suppressing the formation of ordered water clusters and generating more dynamically reconfigurable solvation environments [137]. Such increased local structural adaptability arising from enhanced configurational entropy contributes to improved ion-transport behavior at low temperatures and facilitates more favorable desolvation processes at the electrode/electrolyte interface. Importantly, entropy regulation does not necessarily rely on substantial reconstruction of the primary coordination structure. For example, in the high-entropy second-solvation-shell strategy proposed by Cui et al., the primary Zn^2+^ coordination shell remains largely unchanged, whereas the increased diversity of outer-shell solvation states significantly enhances the configurational entropy of the system [139]. This second-solvation-shell entropy effect weakens the hydrogen-bond network, accelerates Zn^2+^ diffusion, increases ionic conductivity and suppresses hydrogen evolution, demonstrating that entropy regulation can independently influence solvation evolution and ion-transport behavior even when the primary coordination chemistry remains largely preserved. From a thermodynamic perspective, the fundamental role of entropy regulation can be further understood through the Gibbs free-energy relationship, where the entropy term becomes particularly important near phase-transition boundaries [140]. As illustrated in Fig. 8c, entropy regulation increases the number and distribution uniformity of accessible structural states, thereby suppressing low-temperature structural ordering and improving the structural adaptability of electrolyte systems over a wider temperature range [138].

At larger structural scales, entropy regulation is further reflected in topological entropy, which mainly describes the diversity of ion-transport pathways and network connectivity characteristics [141]. HPEs are not homogeneous ion-conduction media, but rather complex multiphase systems consisting of polymer-rich regions, water-rich channels, local coordination sites and dynamically evolving interfacial regions. When such structural heterogeneity is rationally regulated, it can generate multichannel parallel ion-migration pathways and dynamic relaxation modes rather than uncontrolled structural disorder [88, 143, 144]. As summarized in Fig. 8d, mixing entropy, ionic entropy, configurational entropy and topological entropy collectively constitute the multiscale foundation of entropy regulation in HPEs, thereby enabling more adaptive ion transport, homogeneous Zn^2+^ flux and stable interfacial behavior [52, 53].

Overall, entropy regulation in HPEs originates from representative entropy contributions associated with multilevel structural degrees of freedom, including composition, ionic distribution, local configurational states and network topology. These entropy contributions collectively regulate the multiscale distribution and accessibility of water structures, ion-association behavior, solvation configurations and transport pathways. Therefore, entropy regulation should fundamentally be understood as a thermodynamic expansion of accessible physicochemical state space, rather than a simple description of structural disorder.

### Synergistic Integration of C-E Regulation

Although coordination regulation and entropy regulation influence different aspects of electrolyte behavior, the electrochemical performance of HPEs under wide-temperature conditions is not governed by a single regulation mechanism. In practical systems, Zn^2+^ solvation, ion transport, structural relaxation and interfacial evolution are intrinsically interconnected across multiple structural scales [24]. Although these processes are mutually coupled during electrochemical operation, coordination regulation and entropy regulation exhibit different regulatory emphases across different structural scales.

On this basis, the C-E regulation framework can be further understood as a synergistic regulation principle that integrates coordination-governed local interaction energetics with entropy-governed structural-state accessibility. Through this synergistic integration, the framework enables the simultaneous regulation of solvation chemistry, ion-transport behavior and interfacial stability, thereby supporting stable electrochemical behavior of HPEs across wide-temperature ranges.

#### Integrated Roles of C-E Regulation

Within the C-E regulation framework, coordination regulation and entropy regulation govern different yet complementary aspects of electrolyte behavior in HPEs [145]. Coordination regulation primarily determines the local interaction energetics of Zn^2+^ through tuning ligand identity, coordination strength and solvation structures. By reconstructing the local coordination environment among Zn^2+^, water molecules, anions and polymer functional groups, coordination regulation directly influences Zn^2+^ solvation strength, desolvation barriers and interfacial reaction pathways, thereby affecting interfacial stability and charge-transfer behavior. In contrast, entropy regulation primarily governs the distribution characteristics and accessibility of structural states across multiple structural scales [146]. Rather than directly altering local coordination strength, entropy regulation regulates how different solvation structures, polymer conformations and ion-transport pathways are statistically populated during electrochemical operation [94, 121]. These two regulation mechanisms address different limitations in HPE systems and therefore exhibit intrinsic complementarity. Coordination regulation stabilizes energetically favorable local configurations and suppresses undesirable interfacial reactions, but it does not necessarily ensure sufficient structural adaptability and transport accessibility under dynamically changing operating conditions. In contrast, entropy regulation broadens the accessible structural-state space and enhances transport adaptability, but does not directly determine the energetic stability of specific coordination environments or interfacial reaction pathways [147]. Consequently, stable electrochemical behavior over wide-temperature ranges requires the synergistic coupling of both regulation mechanisms.

Through such synergistic integration, local solvation chemistry, structural-state distribution and ion-transport behavior can be cooperatively optimized across multiple structural scales. Coordination regulation primarily stabilizes local solvation environments and interfacial reaction processes, whereas entropy regulation further maintains structural-state accessibility and transport adaptability. The coupling of these two effects links molecular-scale solvation chemistry with macroscopic electrochemical behavior, thereby enabling the simultaneous regulation of Zn^2+^ solvation, ion transport, interfacial stability and deposition behavior across wide-temperature ranges.

Under different temperature conditions, the dominant limitation mechanisms of HPE systems differ significantly. Low-temperature electrochemical behavior is primarily associated with transport accessibility and structural adaptability, whereas high-temperature performance is more strongly governed by solvation stability and interfacial reaction energetics. Consequently, entropy regulation and coordination regulation exhibit different regulatory priorities under different operating conditions. Accordingly, the practical implementation of the C-E regulation framework fundamentally relies on balancing coordination-driven energetic stabilization with entropy-driven structural adaptability.

#### Thermodynamic Effects of C-E Regulation

The electrochemical stability of HPEs across wide-temperature ranges is fundamentally governed by the thermodynamic landscape associated with ion solvation, interfacial reactions and structural stability. Within the proposed C-E regulation framework, coordination regulation and entropy regulation influence different thermodynamic aspects of electrolyte systems and collectively reshape the free-energy landscape governing Zn electrochemistry. From the perspective of coordination regulation, the dominant thermodynamic contribution originates from modulation of the enthalpic interactions associated with Zn^2+^ solvation. In conventional aqueous electrolytes, Zn^2+^ predominantly exists as the strongly hydrated complex [Zn(H_2_O)_6_]^2+^ [145]. Although this coordination structure is thermodynamically stable, the strong Zn–O (H_2_O) interactions impose a substantial enthalpic penalty during interfacial desolvation. Consequently, the removal of coordinated water molecules becomes a key energetic barrier during Zn deposition and stripping [24]. Coordination regulation modifies this enthalpic landscape by introducing alternative coordinating species such as polymer functional groups, anions or solvent molecules into the Zn^2+^ coordination environment. Partial substitution of coordinated water molecules weakens the strong Zn–O (H_2_O) interactions and lowers the enthalpic cost associated with desolvation, thereby improving the thermodynamic feasibility of interfacial electrochemical reactions.

In contrast, entropy regulation primarily affects the configurational thermodynamics of electrolyte systems by expanding the multiplicity and distribution of accessible physicochemical states. This expansion originates from compositional diversity, heterogeneous coordination environments and adaptive polymer-network structures, which collectively increase the number and distribution of accessible states associated with ion solvation, hydrogen-bond rearrangement and structural relaxation [148]. From a statistical thermodynamic perspective, the presence of a larger number of accessible structural states broadens the configurational landscape of the electrolyte system, enabling it to accommodate structural fluctuations and temperature perturbations more effectively [149, 150]. Consequently, entropy regulation suppresses the evolution toward highly ordered structures at low temperatures and mitigates thermally accelerated dissociation or parasitic reactions at elevated temperatures.

The coupled thermodynamic effects of coordination regulation and entropy regulation can be further interpreted through the Gibbs free-energy relationship. Within this framework, coordination regulation primarily modulates ΔH through ion–ligand interactions and solvation energetics, whereas entropy regulation governs ΔS through expansion of the multiscale accessible-state space [133, 150]. Through the synergistic regulation of enthalpic interactions and structural-state multiplicity, the free-energy landscape governing ion solvation, structural stability and interfacial reactions becomes less sensitive to temperature variations, thereby enabling stable electrochemical behavior across a broad temperature range. Under solid–liquid equilibrium conditions (ΔG = 0), the phase-transition temperature can be further expressed as Eq. (11):

11 $$T_{m} = \frac{{{\Delta }H}}{{{\Delta S}}}$$

where both enthalpic and entropic contributions collectively determine phase stability [62]. When the enthalpic contributions of different systems are comparable, increasing the entropy contribution can lower the apparent phase-transition temperature and broaden the stable operating temperature window of the electrolyte [151].

Beyond bulk solvation thermodynamics, the thermodynamic stability of Zn deposition can also be interpreted from the perspective of nucleation thermodynamics. Within classical nucleation theory, the nucleation barrier (ΔG*) depends on the interfacial energy (*γ*) and the volumetric free-energy difference (ΔG_vol_) between the deposited Zn phase and the electrolyte, as shown in Eq. (12):

12 $${\Delta G}^{*} \propto \frac{{{\upgamma }^{3} }}{{({\Delta G}_{{{\mathrm{vol}}}} )^{2} }}$$

Reducing the effective interfacial energy can therefore lower the nucleation barrier and promote thermodynamically favorable uniform Zn nucleation [152]. In HPE systems, coordination regulation reshapes the interfacial energy landscape through coordination interactions between polymer functional groups and Zn species, thereby stabilizing early-stage Zn nuclei. Meanwhile, entropy regulation introduces structurally heterogeneous yet configurationally accessible nucleation environments that help distribute nucleation events more uniformly over the electrode surface. These coupled thermodynamic effects contribute to more homogeneous Zn deposition and enhanced interfacial stability. In addition, coordination regulation governs the thermodynamic evolution of AEI/CEI by reshaping local coordination environments and interfacial reaction pathways. Through modulation of the local coordination environment of Zn^2+^ and surrounding electrolyte species, coordination regulation can thermodynamically favor specific decomposition pathways of water molecules, anions and additives at electrode interfaces. As a result, the interphases formed in HPE systems are not merely kinetically constrained, but are thermodynamically driven toward more compact and chemically stable structures with distinct inorganic/organic components depending on the identities of polymer functional groups, salt anions and additive species.

As a result, the C-E regulation framework establishes a thermodynamic foundation for understanding how coordination regulation and entropy regulation collectively stabilize Zn electrochemistry across wide-temperature ranges. Through synergistic coupling of coordination-governed interaction energetics and entropy-governed structural-state distribution, HPEs can effectively reshape the free-energy landscape governing Zn^2+^ solvation, nucleation and interfacial electrochemical reactions.

#### Kinetic Effects of C-E Regulation

While thermodynamic regulation defines the free-energy landscape governing Zn electrochemistry, the practical reversibility, rate capability and long-term cycling stability of AZIBs are ultimately determined by kinetic processes associated with bulk ion transport and electrode/electrolyte interfacial reactions [153]. Within the proposed C-E regulation framework, coordination regulation and entropy regulation collectively govern the kinetics of Zn^2+^ migration, desolvation, charge transfer and interfacial deposition across multiple structural scales [120].

At the molecular scale, the kinetic barrier associated with Zn deposition is closely related to the desolvation and charge-transfer processes of Zn^2+^ at the electrode surface [150]. In conventional aqueous electrolytes, Zn^2+^ typically exists in the form of the strongly hydrated complex [Zn(H_2_O)_6_]^2+^, and substantial solvent reorganization is required before interfacial electron transfer can occur. This process can be conceptually described using the Marcus–Hush framework, in which the reaction rate is governed by the reorganization energy (*λ*) and the reaction free-energy change (ΔG^0^), as shown in Eq. (13):

13 $${\mathrm{k}}_{{{\mathrm{et}}}} = {\mathrm{k}}_{{\mathrm{o}}} {\mathrm{exp}}\left[ { - \frac{{(\lambda + \Delta {\mathrm{G}}^{0} )^{2} }}{4\lambda RT}} \right]$$

Here, the Marcus–Hush framework is employed to describe solvent-reorganization effects rather than the full mechanistic complexity of Zn electrodeposition [154]. In HPE systems, coordination regulation partially replaces strongly coordinated water molecules in the Zn^2+^ solvation shell with polymer ligands, weakly coordinating anions or dynamically exchangeable species, thereby reducing solvent reorganization during interfacial reactions. This coordination-induced modification effectively lowers the reorganization energy (*λ*) and consequently accelerates interfacial charge-transfer kinetics.

At the mesoscale level, interfacial electrochemical kinetics are commonly described by the Butler–Volmer relationship, in which the exchange current density (i_0_) reflects the intrinsic reaction rate of Zn plating/stripping processes. By regulating Zn^2+^ solvation structures and reducing the desolvation barrier, coordination regulation can effectively increase i_0_, thereby lowering interfacial polarization and overpotential [155]. Beyond interfacial charge-transfer kinetics, the polymer network in HPEs further provides spatial confinement effects and continuous ion-conduction pathways that homogenize Zn^2+^ migration and suppress local electric-field amplification near surface protrusions. These effects collectively promote uniform ionic flux toward the Zn surface and suppress kinetically driven dendritic instability [156, 194].

Entropy regulation further enhances kinetic optimization by introducing multiscale transport-state multiplicity into the electrolyte matrix. Structural heterogeneity across multiple scales generates parallel Zn^2+^ migration pathways with different activation barriers arising from variations in local coordination environments, polymer dynamics and hydrogen-bond exchange processes. Rather than constraining ions to migrate through a single rigid pathway, such multichannel transport networks enable dynamic redistribution of ionic flux in response to local concentration gradients, temperature fluctuations and interfacial perturbations. Consequently, localized ion depletion and kinetic bottlenecks can be effectively alleviated, thereby suppressing concentration polarization and promoting dense lateral Zn growth instead of tip-amplified deposition [157]. From the perspective of ion-transport kinetics, the temperature dependence of ionic migration can be further interpreted using the Arrhenius relationship, as shown in Eq. (14):

14 $$\sigma T = A{\mathrm{exp}}\left( { - \frac{{E_{a} }}{{{\mathrm{RT}}}}} \right)$$

where E_a_ represents the apparent activation energy for ion transport. Within the C-E regulation framework, coordination regulation lowers the local desolvation-related activation barrier through modulation of Zn^2+^ coordination environments, whereas entropy regulation reduces the effective transport barrier by increasing the accessibility of parallel ion-transport pathways [152]. Through synergistic regulation of local desolvation kinetics and multichannel ion transport, the overall ion-transport process becomes kinetically more favorable across wide-temperature ranges. Beyond the Zn anode, similar kinetic principles also apply to cathodic reactions in AZIB systems. Coordination interactions between polymer functional groups and transition-metal active centers can facilitate ion diffusion and reduce redox kinetic barriers within cathode materials, while entropy regulation helps buffer local lattice strain and maintain rapid ion redistribution during dynamic cycling processes. These coupled kinetic effects are particularly important for preserving reversible cathodic reactions under high-rate and wide-temperature operating conditions.

Overall, the C-E regulation framework establishes a kinetic foundation for understanding how coordination regulation and entropy regulation collectively enable fast, uniform and reversible Zn electrochemistry. Through synergistic coupling of molecular-scale solvent-reorganization control and multichannel transport adaptability, HPEs can simultaneously accelerate Zn^2+^ transport, suppress kinetic heterogeneity and stabilize interfacial deposition across broad temperature ranges.

#### Temperature-Dependent Regulation Priorities

The practical implementation of the C-E regulation framework fundamentally relies on the temperature-dependent evolution of dominant physicochemical limitations in HPE systems. Although coordination regulation and entropy regulation remain dynamically coupled throughout electrochemical operation, their regulatory priorities differ under different temperature conditions because the dominant limiting processes vary across temperature regimes. Generally, low-temperature conditions in AZIB systems correspond to temperatures below − 20 °C, with ultralow-temperature operation extending below − 50 °C, whereas elevated-temperature conditions are typically associated with temperatures above 60 °C and may further extend to 80—100 °C in practical operating environments [158]. Under low-temperature conditions, the dominant limitation primarily originates from sluggish ion transport and reduced structural accessibility. In contrast, under elevated-temperature conditions, electrochemical instability is mainly associated with increased water activity and accelerated interfacial parasitic reactions. This difference determines the distinct regulatory priorities of entropy regulation and coordination regulation under different operating conditions.

At low temperatures, ordered hydrogen-bond networks and aggregated ion solvation structures significantly reduce the number and distribution of transport-accessible states, thereby increasing ion-transport resistance and desolvation difficulty [159, 160]. From the perspective of entropy regulation, this process corresponds to reductions in mixing entropy and ionic entropy. Decreased mixing entropy promotes long-range ordering of hydrogen-bond networks, whereas reduced ionic entropy facilitates ion clustering and formation of large aggregated solvation structures. Increasing compositional diversity through multicomponent salts, cosolvents or hybrid polymer systems can effectively disrupt hydrogen-bond ordering and suppress low-temperature structural freezing. Meanwhile, regulation of ion–water and ion–ion interactions can weaken ion association and promote formation of smaller and dynamically exchangeable solvation structures. These effects collectively improve transport accessibility and facilitate Zn^2+^ migration and interfacial desolvation under low-temperature conditions [161]. Therefore, under low-temperature conditions, entropy regulation becomes the dominant regulatory priority because the primary limitation originates from insufficient transport accessibility and structural adaptability.

At elevated temperatures, the dominant limitation shifts from ion-transport restriction to interfacial instability induced by increased water activity [126]. Enhanced water reactivity accelerates parasitic reactions such as hydrogen evolution, corrosion and interfacial side reactions, thereby destabilizing electrode/electrolyte interfaces [162]. This instability fundamentally originates from insufficient regulation of Zn^2+^ solvation structures and interfacial reaction energetics. From the perspective of coordination regulation, introducing coordinating species into the Zn^2+^ solvation environment can reconstruct local coordination structures through modulation of Zn^2+^–ligand interactions. This process weakens Zn^2+^–H_2_O interactions while strengthening Zn^2+^–ligand coordination, thereby reducing water participation within the primary solvation shell and lowering water reactivity [163, 164]. Consequently, more stable interfacial reaction pathways can be established, leading to improved interfacial stability and enhanced cycling durability at elevated temperatures. Therefore, under high-temperature conditions, coordination regulation becomes the dominant regulatory priority because the principal limitation arises from instability of local solvation structures and interfacial reaction energetics.

Importantly, the dominant roles of entropy regulation and coordination regulation under different temperature conditions do not imply independent operation of these two mechanisms. Instead, practical wide-temperature electrolyte design fundamentally relies on balancing coordination-governed energetic stabilization and entropy-governed structural adaptability. Effective HPE systems should therefore simultaneously stabilize Zn^2+^ coordination environments under elevated-temperature conditions while maintaining sufficient transport-accessible states under low-temperature conditions. Through such synergistic regulation, stable ion transport, interfacial compatibility and electrochemical reversibility can be maintained across broad temperature ranges.

#### Parameter-Oriented Design Principles for HPEs

Based on the thermodynamic, kinetic and temperature-dependent regulation mechanisms discussed above, the C-E framework further provides a parameter-oriented design principle for practical HPE development. Within this framework, electrolyte components should not be regarded as isolated material variables, but rather as cooperative structural elements that collectively regulate Zn^2+^ solvation behavior, ion-transport kinetics and interfacial stability [165]. Therefore, rational electrolyte design should be guided by controllable physicochemical parameters instead of empirical optimization of individual materials.

From the perspective of coordination regulation, the key design parameter is the strength and dynamics of Zn^2+^–ligand interactions. Strong Zn^2+^–H_2_O coordination generally leads to high desolvation barriers and severe interfacial parasitic reactions. Introducing functional groups or coordinating species capable of participating in Zn^2+^ coordination can partially replace coordinated water molecules within the primary solvation shell. Functional groups such as –COOH, –CONH_2_, –OH and –SO_3_^−^ in polymer networks, together with weakly or moderately coordinating anions, can provide suitable coordination environments for reconstructing Zn^2+^ solvation structures [132]. Through modulation of Zn^2+^–ligand interactions, coordination regulation weakens Zn^2+^–H_2_O coordination, lowers water activity and stabilizes interfacial reaction pathways, thereby improving interfacial stability under elevated-temperature conditions. Nevertheless, coordination strength must remain balanced because insufficient coordination cannot effectively suppress parasitic reactions, whereas excessively strong coordination may hinder Zn^2+^ mobility and interfacial charge-transfer kinetics.

From the perspective of entropy regulation, the key design principle lies in maintaining sufficient transport-accessible states across multiple structural scales. Increasing compositional diversity through multicomponent salts, cosolvents or hybrid polymer systems can disrupt long-range hydrogen-bond ordering and suppress low-temperature structural freezing [166]. Meanwhile, regulation of ion–water and ion–ion interactions can weaken ion clustering and promote formation of smaller and dynamically exchangeable solvation structures. These effects collectively improve transport accessibility and facilitate Zn^2+^ migration, particularly under low-temperature conditions. Beyond molecular-scale regulation, configurational entropy and topological entropy further provide structural support for maintaining transport adaptability across larger structural scales. Flexible polymer chains, heterogeneous functional-group distributions and dynamic interaction sites broaden the distribution of accessible local configurational states, thereby maintaining segmental dynamics and solvation adaptability [167]. Meanwhile, interconnected polymer networks and spatially distributed ion-conduction domains help preserve continuous ion-transport pathways under temperature fluctuations. Through synergistic regulation of configurational entropy and topological entropy, HPE systems can maintain both structural stability and transport-path continuity across broad operating temperatures.

On the basis of these considerations, electrolyte design under the C-E framework can be guided through several representative physicochemical parameters, including Zn^2+^ coordination strength, ligand identity, water activity, hydrogen-bond-network structure, ion-association behavior, polymer-network flexibility and compositional complexity. Under low-temperature conditions, material design should prioritize entropy regulation through increasing compositional complexity, weakening ion association and enhancing polymer flexibility, thereby maintaining transport accessibility and suppressing freezing-induced structural ordering. In contrast, under elevated-temperature conditions, coordination regulation should be prioritized through introduction of suitable coordinating species that reduce water participation within the primary solvation shell and stabilize interfacial reaction pathways [152]. For practical wide-temperature operation, however, coordination regulation and entropy regulation must be optimized synergistically rather than independently.

Importantly, effective electrolyte design within the C-E framework fundamentally relies on cooperative optimization of coordination-governed interaction energetics and entropy-governed structural-state accessibility. For example, reducing water content may suppress parasitic reactions but can simultaneously decrease ionic conductivity. Such trade-offs can be mitigated through increasing compositional complexity or introducing alternative ion-transport pathways that preserve transport accessibility. Therefore, the essential criterion for practical wide-temperature electrolyte design is whether the electrolyte system can simultaneously stabilize Zn^2+^ coordination environments under elevated-temperature conditions while maintaining sufficient transport-accessible states under low-temperature conditions. This criterion establishes a parameter-guided basis for rational design of wide-temperature hydrogel electrolytes under the C-E framework.

## C-E Regulation Strategies for Wide-Temperature HPEs

In practical hydrogel electrolyte systems, the regulation of Zn^2+^ solvation behavior, ion transport and interfacial stability is closely associated with material chemistry and structural organization across multiple scales [165]. Accordingly, wide-temperature electrolyte design requires cooperative optimization of polymer systems, salt systems, solvent environments, filler regulation and interfacial adaptability. From the perspective of material implementation, different design strategies contribute differently to C-E regulation. Polymer systems primarily regulate Zn^2+^ coordination environments, hydrogen-bond organization and structural adaptability through functional groups, chain dynamics and network structures. Salt systems and solvent environments mainly influence Zn^2+^ solvation behavior, ion association and water activity, thereby regulating ion transport and interfacial reaction pathways [31]. In addition, filler regulation can further modulate ion-transport pathways, mechanical stability and local interfacial environments. In practical systems, however, these physicochemical effects are intrinsically coupled, and stable wide-temperature operation generally relies on the synergistic integration of multiple regulation mechanisms rather than isolated optimization of a single component.

Based on these considerations, the following sections discuss several representative C-E design strategies for wide-temperature HPEs, including polymer system regulation, salt system regulation, cosolvent and eutectic regulation, filler regulation and integrated C-E regulation strategies. Emphasis is placed on how these material-design approaches collectively regulate Zn^2+^ solvation chemistry, transport behavior and interfacial electrochemical stability under wide-temperature conditions.

### Polymer System Regulation

Polymer systems play a central role in C-E regulation for wide-temperature HPEs. Through modulation of functional groups, chain structures and polymer-network compositions, polymer systems can simultaneously influence Zn^2+^ solvation behavior, hydrogen-bond organization, ion-transport pathways and interfacial stability [68]. Within the C-E framework, different polymer-design strategies usually exhibit different dominant regulatory characteristics. Functional-group engineering primarily realizes coordination regulation by modulating Zn^2+^-ligand interactions and local solvation structures, thereby weakening Zn^2+^–H_2_O interactions, lowering the desolvation barrier and stabilizing interfacial reaction processes [78]. In contrast, multicomponent and structurally complex polymer networks mainly realize entropy regulation through redistribution of ion configurations, hydrogen-bond structures and ion-transport pathways, thereby alleviating local ion aggregation, maintaining ion-transport continuity and improving structural adaptability under temperature variation. In practical polymer-based HPE systems, coordination regulation and entropy regulation are intrinsically coupled. Polymer functional groups and associated ionic interactions can reconstruct Zn^2+^ solvation structures and regulate interfacial reaction pathways, while diverse polymer networks and complex intermolecular interactions help homogenize Zn^2+^ transport and stabilize interfacial processes. As a result, polymer systems can maintain ion transport and stable interfacial contact under low-temperature conditions, while suppressing water-induced parasitic reactions and interfacial instability at elevated temperatures, thereby improving electrochemical reversibility and cycling stability across wide-temperature ranges.

Based on their dominant regulatory characteristics, polymer system regulation strategies can be further categorized into functional-group engineering and multicomponent polymer-network design. The following sections discuss how these strategies cooperatively regulate Zn^2+^ solvation chemistry, ion-transport behavior and interfacial electrochemical stability within the C-E framework.

#### Functional-Group Engineering

Functional-group engineering represents one of the most direct strategies for regulating Zn^2+^ solvation behavior and interfacial reactions in polymer-based HPE systems. By introducing coordination-active groups into polymer chains, the local chemical environment surrounding Zn^2+^ can be effectively reconstructed, thereby influencing Zn^2+^–ligand interactions, water activity and interfacial electrochemical processes. Representative functional groups, including carboxyl [118], sulfonate [168], phosphate, imidazole or trifluoroborate groups [162], can partially replace H_2_O molecules within the primary solvation shell of Zn^2+^, weaken Zn^2+^–H_2_O interactions and reduce the desolvation barrier associated with Zn^2+^ transport. Meanwhile, changes in local interaction environments and water organization can further influence ion distribution and transport behavior under temperature variation.

**Fig. 9:**
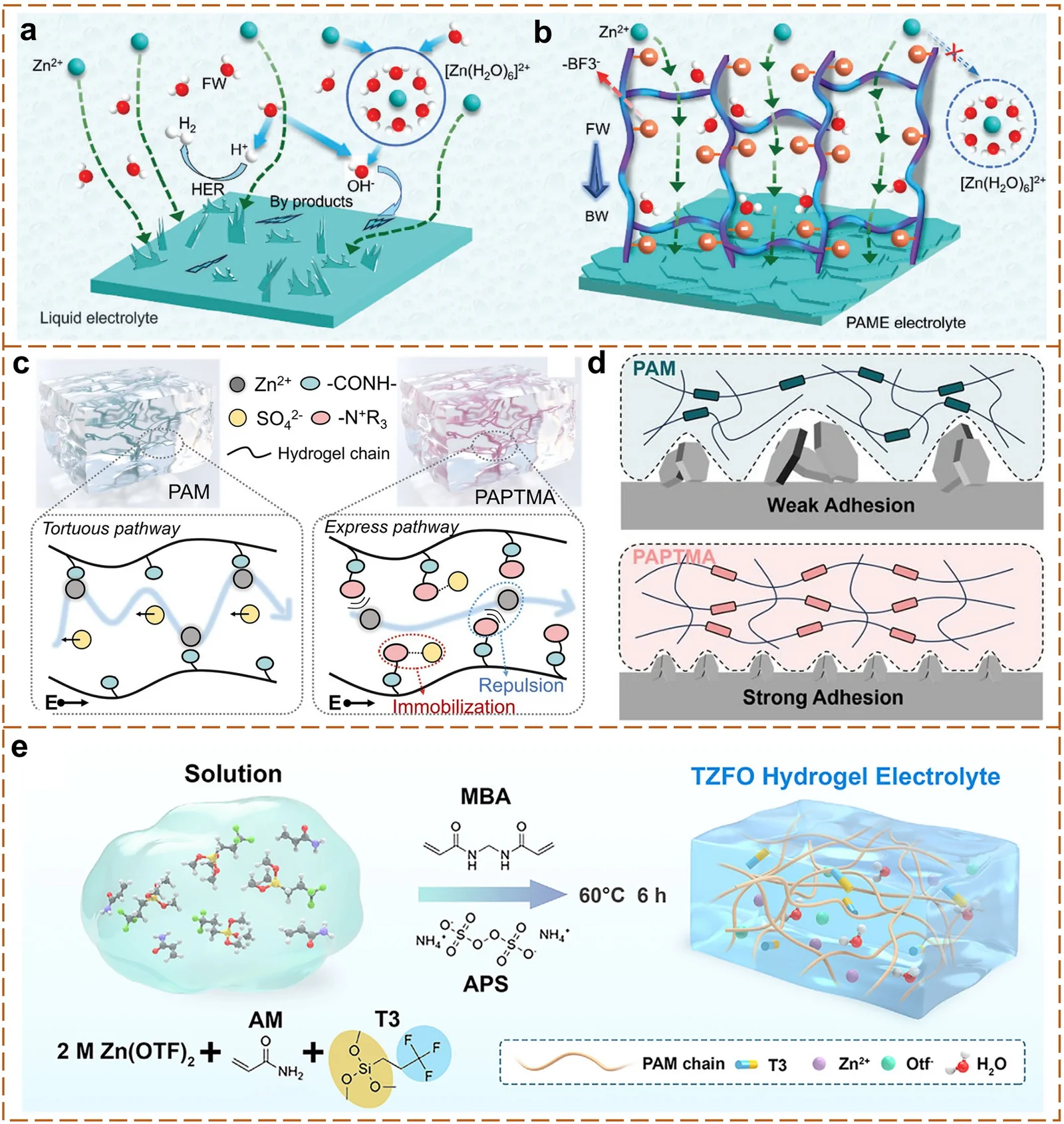
Molecular-level functional-group regulation strategies for wide-temperature HPEs.** a** Schematic illustration of nonuniform Zn deposition and interfacial instability in conventional liquid electrolytes. **b** Coordination-regulated Zn deposition and homogenized ion flux in the PAME hydrogel electrolyte [162]. Copyright 2024, Wiley. **c** Morphology and ion-transport pathways in PAM and PAPTMA hydrogels. **d** Schematic illustration of Zn plating behavior at the hydrogel/Zn interface in PAM and PAPTMA systems [79]. Copyright 2025, Springer Nature. **e** Structural design and wide-temperature mechanical adaptability of the TZFO hydrogel electrolyte [169]. Copyright 2026, Wiley

Representative systems clearly demonstrate these coupled effects. Yan et al. designed a trifluoroborate-terminated polyacrylamide hydrogel electrolyte, in which the -BF_3_^−^ groups along the polymer backbone strongly coordinate with Zn^2+^ [162]. In conventional aqueous electrolytes, continuous water dissociation near the Zn surface accelerates hydrogen evolution, corrosion and by-product formation (Fig. 9a). In contrast, strong polymer–water interactions in the polyacrylamide electrolyte (PAME) hydrogel convert free water into bound water and reduce water activity (Fig. 9b). At the same time, the –BF_3_^−^ groups partially replace coordinated water molecules in the Zn^2+^ solvation shell, thereby weakening Zn–O (H_2_O) interactions and facilitating interfacial desolvation and charge transfer. Molecular dynamics simulations further confirm the formation of stronger Zn–F interactions compared with Zn–O (H_2_O) interactions, indicating reconstruction of the local coordination environment. In addition, the coexistence of multiple coordination sites suppresses localized Zn^2+^ accumulation and homogenizes Zn^2+^ flux, thereby stabilizing Zn deposition behavior. These effects are particularly beneficial under elevated-temperature conditions, where increased water activity would otherwise intensify parasitic reactions and interfacial instability. Similarly, Zhang et al. developed a polyacrylamide derivative rich in quaternary-ammonium groups, in which ionic domains exhibit strong hydration capability (Fig. 9c, d) [79]. These cationic groups convert free water into interfacial bound water, thereby suppressing hydrogen evolution and corrosion reactions. The redistribution of water environments weakens Zn–O (H_2_O) interactions and indirectly lowers the desolvation barrier during Zn^2+^ transport. In addition, strong interfacial adhesion between the polymer and Zn surface further homogenizes Zn^2+^ flux and mechanically suppresses dendritic growth. As a result, the system exhibits improved cycling stability and enhanced reversibility of Zn plating/stripping processes.

Beyond regulation of local coordination environments, functional-group engineering can also simultaneously regulate water structure and temperature adaptability. Han et al. reported a fluorosilane-functionalized hydrogel electrolyte containing trimethoxy(3,3,3-trifluoropropyl)silane within a polyacrylamide network [169]. In this system, hydrophobic –CF_3_ groups suppress water activity at elevated temperatures, thereby reducing parasitic reactions and stabilizing interfacial processes. Meanwhile, siloxane-related groups provide coordination sites for Zn^2+^ and stabilize local solvation structures. At the same time, the coexistence of hydrophobic domains and coordination-active sites helps maintain continuous ion transport under low-temperature conditions. Consequently, the electrolyte enables stable electrochemical operation over a wide-temperature range from -20 to 80 °C. Zn||NVO full cells maintain stable cycling for over 5400 cycles at -20 °C and retain a capacity of 153.0 mAh g^−1^ after 300 cycles at 80 °C, demonstrating simultaneous stabilization of low-temperature ion transport and high-temperature interfacial processes.

Collectively, functional-group engineering mainly regulates Zn^2+^ solvation structures and interfacial reaction pathways through modulation of Zn^2+^–ligand interactions and local water environments, and therefore, its regulatory effect is primarily dominated by coordination regulation. These effects are particularly beneficial for reducing water activity, suppressing water-induced side reactions and stabilizing interfacial electrochemical processes under elevated-temperature conditions. In contrast, the influence of this strategy on ion-configuration redistribution and transport-pathway reconstruction is relatively limited, and thus, entropy regulation does not play a dominant role in such systems.

#### Multicomponent Polymer Networks Design

Multicomponent polymer networks provide an important design strategy for regulating Zn^2+^ solvation, ion transport and interfacial stability in wide-temperature HPE systems. Unlike functional-group engineering, which mainly relies on localized Zn^2+^–ligand interactions, multicomponent polymer networks more strongly depend on network complexity, compositional diversity and heterogeneous interaction environments to regulate ion-transport behavior [109, 170]. Hydrogen bonding, electrostatic interactions and polymer–ion interactions formed among different polymer components can generate spatially heterogeneous ion-transport domains and diversified local interaction environments, thereby increasing the distribution diversity of accessible Zn^2+^ transport pathways and local ionic configurations [99]. Such coexistence of multiple components and local states contributes primarily to mixing entropy, configurational entropy and topological entropy, which helps alleviate localized ion aggregation and maintain continuous ion transport under low-temperature conditions. Therefore, this type of strategy is more closely associated with entropy regulation for improving transport continuity and structural adaptability at subzero temperatures. Meanwhile, polymer functional groups and associated ionic interactions within multicomponent systems can still participate in regulating Zn^2+^ solvation structures, and thus, coordination-related effects are also involved.

**Fig. 10:**
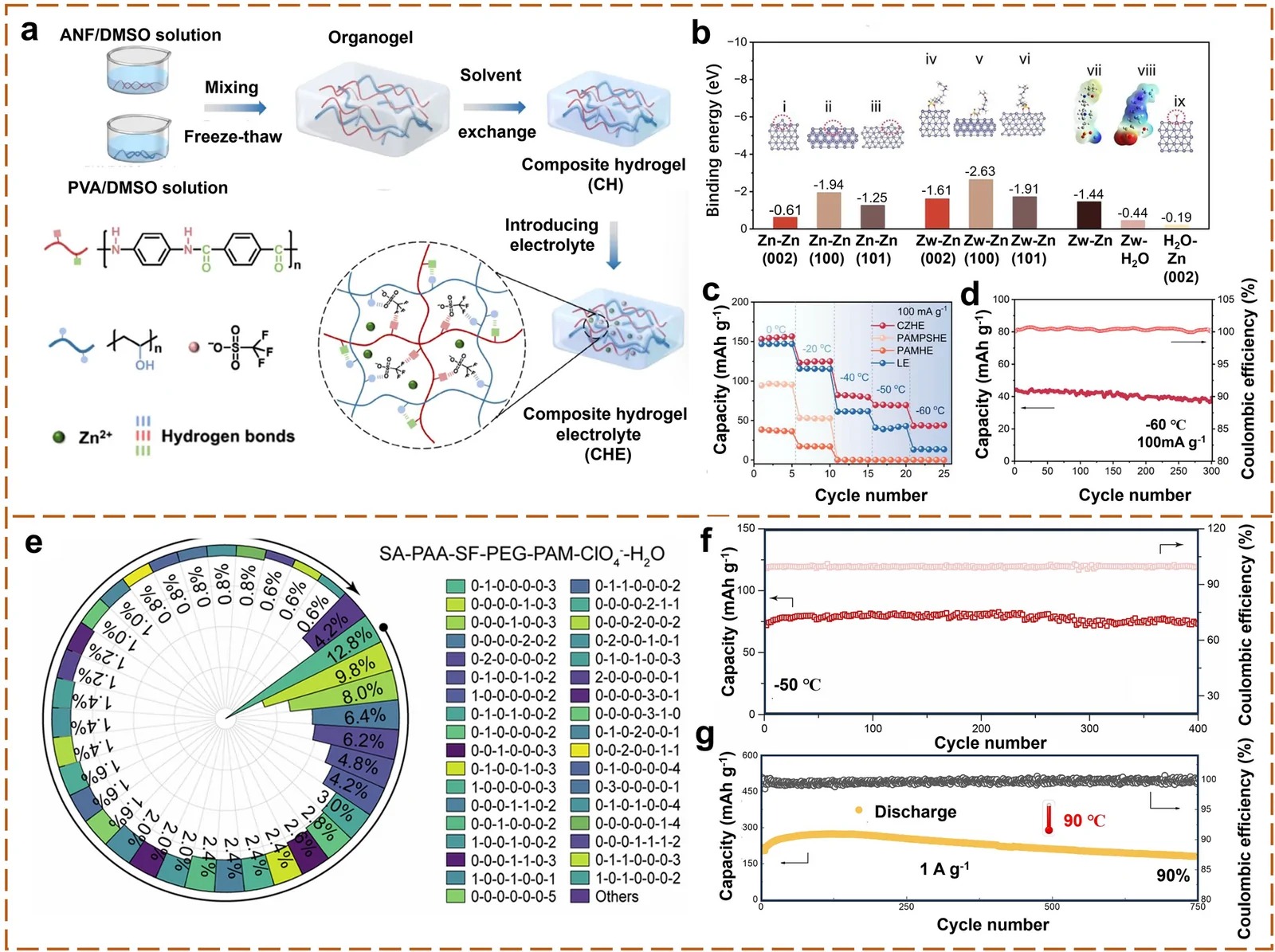
Multicomponent polymer-network engineering strategies for wide-temperature hydrogel electrolytes.** a** Schematic illustration of the preparation and multicomponent structure of the CHE hydrogel electrolyte [171]. Copyright 2025, Wiley. **b** DFT-calculated adsorption configurations of zwitterionic monomers on different Zn crystal planes. **c** Temperature-dependent electrochemical performance of Zn||PANI full cells with CZHE. **d** Stable low-temperature cycling performance of Zn||PANI full cells at -60 °C [172]. Copyright 2025, Springer Nature. **e** Zn^2+^ coordination configurations derived from MD simulations in multicomponent hydrogel electrolytes. **f** Stable low-temperature cycling performance of full cells with HEE at -50 °C [137]. Copyright 2026, American Chemical Society. **g** Long-term cycling performance of full cells using the HPG-35 wt% hydrogel electrolyte at 90 °C, demonstrating enhanced high-temperature stability [149]. Copyright 2025, Cell Press

Zou et al. developed a multicomponent hydrogel composed of aramid nanofibers (ANF), polyvinyl alcohol (PVA) and Zn(OTf)_2_ (Fig. 10a) [171]. Hydrogen-bonding and electrostatic interactions immobilize anions and construct continuous ion-transport channels. Polymer–anion interactions partially replace coordinated water molecules within the Zn^2+^ solvation shell, thereby reducing water activity and facilitating interfacial charge transfer. Meanwhile, the interpenetrating network introduces spatially heterogeneous ion-transport domains and diversified local interaction environments, enabling Zn^2+^ to dynamically migrate through multiple local transport pathways. The resulting increase in local-state distribution and pathway diversity reflects configurational entropy and topological entropy regulation, which helps suppress localized ion accumulation and homogenize Zn^2+^ flux. Consequently, the electrolyte exhibits high ionic conductivity (42.2 mS cm^−1^), Zn^2+^ transference number of 0.78 and excellent cycling stability with 78% capacity retention after 9100 cycles, demonstrating efficient ion transport and stable interfacial behavior. Beyond bulk ion transport, multicomponent polymer networks can also directly regulate interfacial Zn deposition behavior. Pei et al. developed a crowded zwitterionic hydrogel electrolyte in which polymer–ion interactions significantly modify Zn deposition processes [172]. Density functional theory (DFT) calculations reveal that Zn^2+^ preferentially deposits on high-energy (100) and (101) facets on bare Zn, leading to dendrite growth, whereas the zwitterionic polymer selectively adsorbs on these facets and suppresses their growth, thereby guiding Zn deposition toward the (002) orientation (Fig. 10b). Meanwhile, crowded ionic environments and diversified local interaction sites increase the distribution of accessible local states and transport pathways near the interface, helping homogenize interfacial Zn^2+^ flux and suppress localized Zn^2+^ accumulation. As a result, the crowded Zw hydrogel electrolytes (CZHE)-based Zn||PANI battery delivers 44 mAh g^−1^ at—60 °C and maintains stable cycling over 300 cycles with nearly 100% Coulombic efficiency (Fig. 10c, d), demonstrating excellent low-temperature interfacial stability and dendrite suppression capability.

As compositional complexity further increases, multicomponent polymer networks exhibit more pronounced regulation of Zn^2+^ solvation–desolvation kinetics and interfacial processes. Chen et al. constructed a five-component polymer-network hydrogel system in which multiple polymer–ion and polymer–water interactions cooperatively regulate Zn^2+^ solvation structures (Fig. 10e) [137]. Increased compositional complexity redistributes Zn^2+^ solvation configurations and local coordination states, thereby lowering desolvation-related free-energy barriers and facilitating Zn^2+^ transport and interfacial reactions. Such coexistence of multiple local states contributes to configurational entropy and mixing entropy regulation, helping establish a more dynamic Zn^2+^ transport environment. Molecular dynamics simulations show that multiple functional groups participate in constructing the Zn^2+^ solvation shell and partially replace coordinated water molecules, forming more weakly bound solvation structure. Spectroscopic analyses further confirm weakened Zn–O coordination and reduced ion pairing. Meanwhile, TEM observations reveal a thin and predominantly amorphous solid electrolyte interphase (SEI) layer, while XPS depth profiling demonstrates that the interphase is mainly composed of organic species derived from polymer functional groups. Compared with conventional inorganic-dominated SEI layers, this organic-rich interphase effectively lowers interfacial resistance and suppresses polarization. Consequently, the system retains over 40% of its room-temperature capacity at − 50 °C and maintains stable cycling over 400 cycles (Fig. 10f), demonstrating excellent low-temperature ion transport and interfacial stability.

In addition to low-temperature performance, multicomponent polymer networks also exhibit excellent stability under elevated-temperature conditions. Zhi et al. designed a lean-water hydrogel electrolyte based on PMEM and HEAA [149]. Flexible ether-oxygen groups facilitate Zn^2+^ transport, while hydrophilic groups reduce water activity and suppress parasitic reactions through strong polymer–water interactions. Meanwhile, the adaptive polymer network maintains continuous ion-transport pathways under low-water-content conditions. As a result, the electrolyte achieves nearly 99% Coulombic efficiency at 90 °C, while the corresponding full cells retain approximately 90% capacity after 750 cycles (Fig. 10g), demonstrating stable interfacial processes and ion-transport behavior at elevated temperatures.

Collectively, multicomponent polymer networks mainly regulate ion-transport behavior and interfacial stability through spatially heterogeneous transport domains and diversified local interaction environments. Their regulatory effects are therefore more strongly associated with entropy regulation, particularly through modulation of ionic-state distributions and transport accessibility involving configurational, mixing and topological entropy. Such strategies are especially beneficial for maintaining continuous ion transport, alleviating localized ion aggregation and improving structural adaptability under low-temperature conditions. Meanwhile, polymer–ion interactions within multicomponent networks can still participate in regulating Zn^2+^ solvation structures, thereby cooperatively stabilizing interfacial processes and Zn deposition behavior.

### Salt System Regulation

Salt system regulation represents an important strategy for regulating Zn^2+^ solvation structures and interfacial stability in wide-temperature HPEs. Unlike polymer systems, which mainly regulate transport environments and structural adaptability, salt systems directly influence Zn^2+^ solvation behavior, ion-association states and water activity through anion–cation interactions [52, 53]. By tuning salt identity, concentration and multi-ion interactions, salt systems can simultaneously regulate Zn^2+^ coordination environments, ion-migration behavior and interfacial reaction processes [173]. Within the C-E framework, coordination regulation in salt systems mainly originates from anion–Zn^2+^ interactions that reconstruct Zn^2+^ solvation structures. Certain anions can partially replace coordinated water molecules within the primary Zn^2+^ solvation sheath, thereby weakening Zn^2+^–H_2_O interactions, lowering desolvation barriers and stabilizing interfacial reaction processes [167, 174]. Meanwhile, multiple ion-association structures in multi-anion systems, including solvent-separated ion pairs (SSIPs), contact ion pairs (CIPs) and aggregates (AGGs), together with multicomponent ionic environments, increase the distribution diversity of Zn^2+^ solvation configurations, local ionic states and transport pathways [175]. Among these effects, multi-anion coexistence mainly contributes to mixing entropy regulation, diversified solvation configurations correspond to configurational entropy regulation, while distributed local ionic environments and ion-migration pathways further reflect ionic entropy and topological entropy regulation. These effects collectively help alleviate localized ion aggregation, homogenize Zn^2+^ flux and maintain continuous ion transport and stable interfacial processes under temperature variation.

Based on their dominant regulatory characteristics, salt system regulation can be further classified into single-salt systems and multi-salt systems. Single-salt systems mainly regulate Zn^2+^ solvation structures through anion–Zn^2+^ coordination interactions and are therefore more closely associated with coordination regulation. In contrast, multi-salt systems introduce higher compositional complexity and competitive ionic interactions, which promote the formation of diversified solvation states and ionic environments, making entropy regulation increasingly important.

#### Single-Salt Regulation

Within the C-E framework, single-salt hydrogel electrolytes can be regarded as representative model systems for investigating Zn^2+^ solvation structures and interfacial reaction behaviors in Zn-based batteries. In such systems, Zn^2+^ mainly exists in the form of highly hydrated [Zn(H_2_O)_6_]^2+^ complexes, where strong Zn^2+^–H_2_O interactions lead to high desolvation barriers and sluggish interfacial charge-transfer kinetics [24]. Meanwhile, excessive free water can further induce hydrogen evolution, by-product formation and Zn corrosion. Therefore, the primary regulatory objective in single-salt systems mainly focuses on reconstructing Zn^2+^ solvation structures, suppressing water activity and stabilizing interfacial reactions, making their regulatory mechanism more strongly associated with coordination regulation. In single-salt systems, Zn^2+^ solvation structures are mainly dominated by a single-anion environment. Anion–Zn^2+^ interactions can partially reconstruct Zn^2+^ hydration structures, thereby weakening Zn^2+^–H_2_O interactions and lowering desolvation barriers, which helps suppress hydrogen evolution, by-product formation and Zn corrosion. When further coupled with polymer networks, the confinement effect of polymers on water molecules can reduce free-water content and stabilize interfacial processes to a certain extent. However, because these systems mainly contain only a single ionic environment, the distributions of Zn^2+^ solvation configurations and local ionic states remain relatively limited. As a result, the regulatory mechanism in such systems is still dominated by coordination regulation, while entropy-regulation effects remain comparatively weak.

For example, PAM-based and modified PVA-based single-salt hydrogel systems have been widely employed to regulate Zn^2+^-polymer coordination interactions [83, 104]. In these systems, polymer chains can partially participate in constructing Zn^2+^ solvation structures, thereby suppressing hydrogen evolution and improving Zn deposition uniformity. Meanwhile, polymer networks can also help maintain continuous ion-transport pathways to a certain extent. However, because these systems mainly contain only a single-anion environment, the distributions of Zn^2+^ solvation configurations and local ionic states remain relatively limited, and entropy-regulation effects are therefore less pronounced. Such limited state distributions restrict the flexibility of Zn^2+^ redistribution and transport-pathway regulation through ionic-environment reconstruction, thereby limiting transport adaptability under extreme temperature conditions. Recent studies further suggest that when coupled with appropriate polymer networks, single-salt systems can still achieve improved interfacial stability at elevated temperatures. For example, Yao et al. reported a cellulose-based hydrogel electrolyte containing highly concentrated ZnCl_2_, which maintained structural stability at temperatures up to 120 °C [176]. In this system, the highly concentrated ZnCl_2_ effectively suppresses water activity and stabilizes Zn^2+^ solvation structures, while the polymer network preserves structural integrity at elevated temperatures through dynamic interactions. Consequently, the corresponding zinc-ion hybrid supercapacitor retained 91.1% of its capacity after 10,000 cycles at room temperature and still maintained 85.4% capacity retention after 10,000 cycles at 60 °C. These results indicate that although single-salt systems mainly rely on coordination regulation to stabilize Zn^2+^ solvation and interfacial processes, polymer networks remain essential for maintaining structural stability and interfacial integrity under high-temperature conditions.

As a result, single-salt systems mainly regulate Zn^2+^ solvation structures and interfacial reaction processes through Zn^2+^–anion and Zn^2+^–polymer interactions, and their regulatory mechanism is therefore dominated by coordination regulation. Such systems are particularly beneficial for reducing water activity, suppressing parasitic reactions and stabilizing interfacial electrochemical behavior at elevated temperatures. However, because their ionic environments and solvation-state distributions remain relatively limited, entropy-regulation effects are comparatively weak, resulting in limited transport adaptability and structural dynamic regulation under low-temperature conditions.

#### Multi-Salt Regulation

To overcome the intrinsic limitations of single-salt electrolytes, multi-salt systems introduce higher compositional complexity and competing ionic interactions into Zn^2+^ solvation environments. Compared with single-salt systems dominated by a single ionic environment, multi-salt systems can generate more diversified ion-association structures and local ionic states, thereby simultaneously regulating Zn^2+^ solvation chemistry, ion-transport behavior and interfacial processes [167]. Consequently, although coordination regulation remains essential in multi-salt systems, entropy regulation becomes increasingly important due to the emergence of diversified solvation configurations and distributed ionic environments. In multi-salt systems, different anions can competitively participate in Zn^2+^ solvation structures and partially replace coordinated water molecules within the primary solvation sheath, thereby weakening Zn^2+^–H_2_O interactions. Such anion–Zn^2+^ interactions reduce desolvation barriers and stabilize interfacial reactions, representing the coordination-regulation effect in salt chemistry. Meanwhile, coexistence of multiple ion-association structures, including SSIPs, CIPs and AGGs, further increases the distribution diversity of Zn^2+^ solvation configurations and local ionic environments [177]. In this process, coexistence of multiple anions mainly contributes to mixing entropy regulation through increased compositional complexity in the bulk electrolyte, while diversified Zn^2+^ solvation configurations correspond primarily to configurational entropy regulation. These configurational variations can be reflected through experimentally and computationally accessible structural descriptors, including coordination-number distributions, radial distribution functions (RDF) analysis and ion-association statistics. In addition, distributed ionic environments and transport pathways further reflect ionic entropy and topological entropy regulation, which are associated with redistribution of ion-association behavior and transport accessibility.

**Fig. 11:**
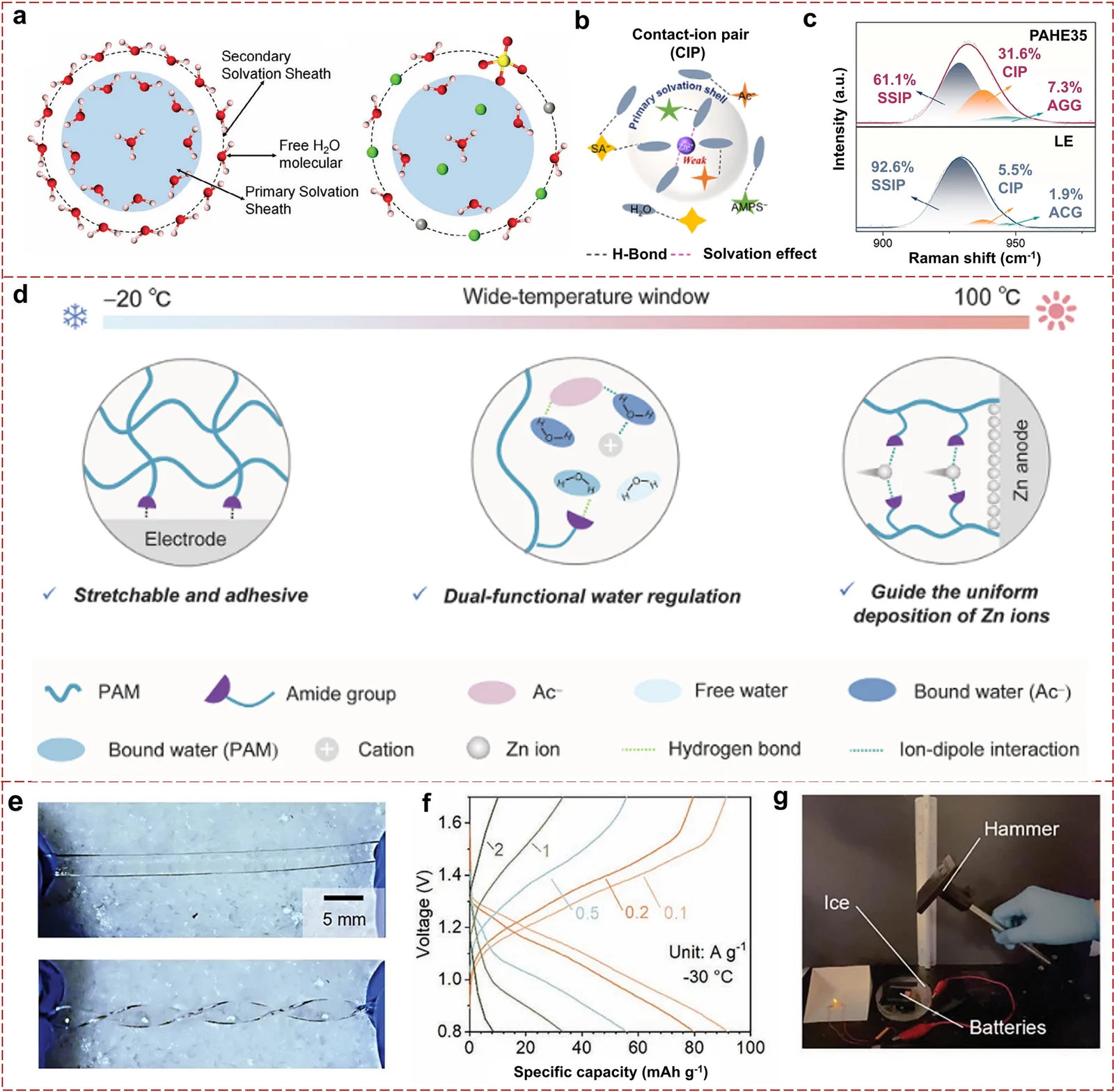
Multi-salt regulation strategies for wide-temperature hydrogel electrolytes.** a** Schematic illustration of low-temperature solvation-structure evolution in acid–salt hybrid electrolytes [173]. Copyright 2025, Wiley. **b** Schematic illustration of contact ion-pair (CIP) formation in multi-anion electrolytes. **c** Raman spectra of acetate-associated C–C vibrations, revealing ion-association evolution [126]. Copyright 2025, Wiley. **d** Structural design and multifunctional properties of the PAM-ZnK_4_Ac HPEs [53]. Copyright 2025, Wiley. **e** Photographs of the PVA-Zn(Ac)_2_/KAc hydrogel twisted at -30 °C, demonstrating excellent antifreezing capability. **f** Galvanostatic charge–discharge curves of Zn||PVA-416||PANI batteries at -30 °C. **g** Soft-pack battery demonstrating impact resistance enabled by the mechanically robust and thermally stable PVA-416 HPEs [52]. Copyright 2023, Wiley

Based on this C-E regulatory principle, Yu et al. reported an acid–salt hybrid electrolyte composed of H_2_SO_4_ and ZnCl_2_, where Zn^2+^, in which Zn^2+^ ions, anions and water molecules formed a cooperative solvation network (Fig. 11a) [173]. In this system, competitive coordination interactions between different anions and Zn^2+^ reconstruct the Zn^2+^ solvation structure and facilitate desolvation processes. Meanwhile, coexistence of multiple local ionic environments broadens the distribution of solvation states, thereby improving low-temperature proton transport and ionic mobility. This system represents a typical example in which coordination regulation and entropy regulation coexist in multi-anion electrolytes. Building on this strategy, Yang et al. further proposed a dual-triggered hydrogen evolution suppression mechanism by distinguishing solvated water from free water (Fig. 11b) [126]. Raman spectroscopy further confirmed progressive evolution of solvation structures (Fig. 11c), where multiple anions (Ac^−^, SA^−^ and AMPS^−^) effectively reduced free-water content and promoted formation of CIPs and AGGs. Coexistence and dynamic interconversion of these ion-association structures increased the distribution diversity of local ionic states and Zn^2+^ solvation configurations, corresponding mainly to configurational entropy and mixing entropy regulation. Meanwhile, distributed ion-association environments further reduced local transport anisotropy and stabilized Zn deposition behavior. Consequently, Zn/PAHE₃₅/LFP full cells exhibited excellent cycling durability (> 10,000 cycles) and maintained high-rate capability even at − 20 °C, demonstrating stable interfacial processes under low-temperature conditions.

Further extending this regulatory principle, Chen et al. designed a quasi-solid PAM-ZnK_4_Ac hydrogel electrolyte, in which hydrogen-bond interactions between acetate anions and amide groups help stabilize Zn^2+^ solvation structures and reduce desolvation barriers (Fig. 11d) [53]. Meanwhile, coexistence of multiple ionic species and distributed ion-transport environments promotes redistribution of local ionic states and transport pathways, thereby enhancing entropy-related regulation. The electrolyte delivered high ionic conductivity (34.7 mS cm^−1^) and remarkable thermal adaptability, maintaining stable operation from − 20 to 100 °C. In Zn||PANI full cells, the battery retained 81.4% of its capacity after 1100 cycles, demonstrating stable ion transport and interfacial processes under extreme thermal conditions. He et al. further developed a PVA-Zn(Ac)_2_/KAc hydrogel electrolyte (PVA-416) by utilizing the salting-out effect of KAc to further reduce water activity and stabilize electrolyte structures (Fig. 11e) [52]. In this system, competitive ion interactions regulate Zn^2+^ solvation structures and desolvation kinetics, while coexistence of multiple ionic environments further broadens accessible ion-transport pathways and local ionic-state distributions. These effects collectively contribute to configurational entropy and topological entropy regulation, thereby reducing local transport anisotropy and promoting homogeneous Zn^2+^ transport. As a result, the hydrogel exhibited excellent antifreezing capability and mechanical resilience, remaining ductile even at -30 °C while maintaining high discharge capacities across a wide current–density range (0.1–5 A g^−1^). Even at − 30 °C, the cells maintained stable cycling with small polarization and stable charge–discharge profiles (Fig. 11f). In addition, pouch-cell impact tests revealed an approximately eightfold enhancement in shock tolerance (Fig. 11g), demonstrating synergistic stabilization of mechanical robustness and electrochemical stability under wide-temperature conditions.

Accordingly, multi-salt systems simultaneously regulate Zn^2+^ solvation chemistry, ionic-state distributions and transport pathways through coupled coordination regulation and entropy regulation. Coordination regulation mainly stabilizes Zn^2+^ solvation structures and suppresses water-induced parasitic reactions, while entropy regulation broadens distributions of solvation configurations, local ionic environments and transport pathways, thereby enabling uniform Zn^2+^ transport, continuous ion conduction and stable interfacial processes across wide-temperature ranges. Therefore, multi-salt regulation represents an important strategy for designing wide-temperature hydrogel electrolytes with both thermodynamic stability and transport adaptability.

### Cosolvent Regulation

Cosolvent regulation represents an important strategy for regulating water structures, hydrogen-bond networks and Zn^2+^ solvation environments in wide-temperature HPEs. Unlike polymer systems that mainly regulate structural organization and ion-transport environments, cosolvent systems primarily influence Zn^2+^ solvation behavior, water activity and interfacial reaction processes through reconstruction of local solvent environments and hydrogen-bond interactions [163, 178]. By introducing small-molecule components with strong hydrogen-bonding capability or eutectic characteristics, these systems can simultaneously regulate Zn^2+^ coordination environments, liquid-state stability and ion-migration behavior, thereby improving ion-transport continuity and interfacial stability across wide-temperature ranges. Within the C-E framework, coordination regulation in cosolvent systems mainly originates from interactions between small-molecule components and Zn^2+^ ions or water molecules. Certain organic molecules can participate in Zn^2+^ solvation structures and partially replace coordinated water molecules, thereby weakening Zn^2+^–H_2_O interactions, lowering desolvation barriers and suppressing water-induced parasitic reactions. Meanwhile, multicomponent hydrogen-bond networks and dynamic solvent environments further reconstruct water organization and broaden distributions of local liquid states and ion-transport states [59]. Among these effects, hydrogen-bond-network reorganization mainly contributes to configurational entropy regulation, while multicomponent eutectic environments and diversified liquid-state structures further reflect mixing entropy and topological entropy regulation. These entropy-related effects help suppress structural ordering and water crystallization at low temperatures while maintaining continuous ion transport and stable interfacial processes under temperature variation.

Based on their dominant regulatory characteristics, cosolvent regulation can be further classified into organic/bio-inspired cosolvent systems and eutectic systems. Organic and bio-inspired molecules mainly regulate local solvent environments through dynamic hydrogen-bond-network reorganization and are therefore more closely associated with configurational entropy regulation. In contrast, eutectic systems stabilize multicomponent liquid structures and exhibit more pronounced mixing entropy regulation. The following sections discuss how these two representative strategies regulate Zn^2+^ solvation, ion transport and interfacial stability in wide-temperature HPEs.

#### Organic and Bio-Inspired Cosolvents

Organic and bio-inspired cosolvent systems represent an important strategy for regulating local solvent environments and hydrogen-bond networks in wide-temperature HPEs. Unlike inorganic fillers that mainly rely on rigid structural confinement, organic small molecules mainly influence Zn^2+^ solvation behavior and interfacial reaction processes through dynamic hydrogen-bond interactions, water-structure reconstruction and local solvent-environment regulation [164, 179]. Within the C-E framework, coordination regulation in these systems mainly originates from interactions between organic molecules and Zn^2+^ ions or water molecules. Certain organic molecules can participate in Zn^2+^ solvation structures and partially replace coordinated water molecules, thereby weakening Zn^2+^–H_2_O interactions, lowering desolvation barriers and suppressing water-induced parasitic reactions. Meanwhile, dynamic hydrogen-bond networks and local solvent-environment reconstruction further increase the distribution diversity of water-organization states and local liquid-state structures, mainly corresponding to configurational entropy regulation. These effects help maintain continuous ion transport and stable interfacial processes under low-temperature conditions. Therefore, such systems generally exhibit more pronounced entropy-regulation characteristics.

**Fig. 12:**
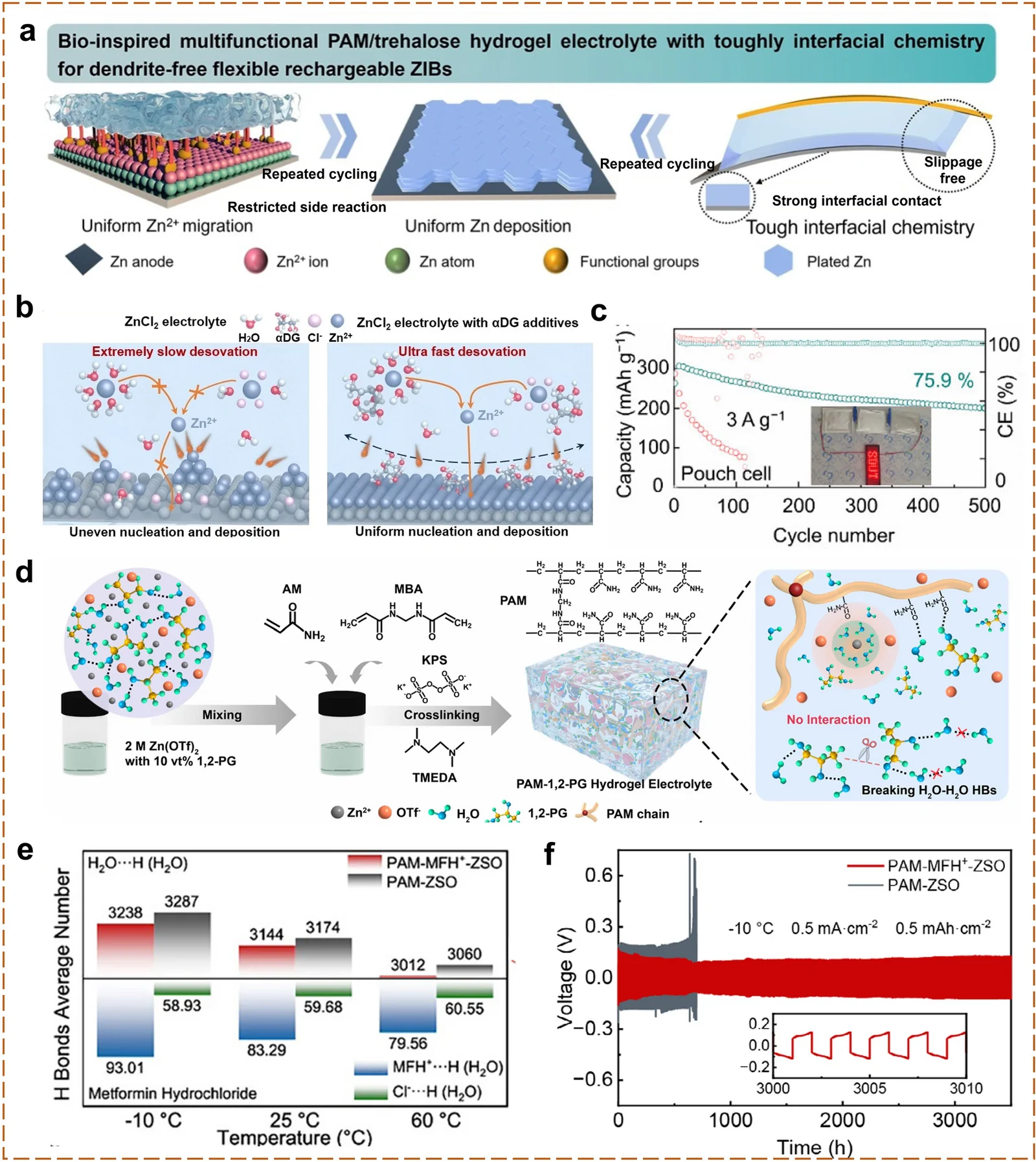
Organic and bio-inspired modification strategies for wide-temperature hydrogel electrolytes.** a** Zn deposition behavior and interfacial chemistry of trehalose-modified PAM hydrogels in flexible ZIBs [59]. Copyright 2024, Wiley. **b** Interfacial configuration of Zn anodes in ZnCl_2_-αDG electrolytes at low temperatures, showing hydrogen-bond and solvation reconstruction [157]. Copyright 2024, Wiley. **c** Cycling performance of pouch-type Zn batteries using trehalose-modified hydrogels, exhibiting long-term stability [106]. Copyright 2024, American Chemical Society. **d** Preparation and structural schematic of PAM-1,2-PG hydrogels [180]. Copyright 2024, Royal Society of Chemistry. **e** MD-simulated temperature-adaptive hydrogen-bond network in PAM-MFH^+^-ZnSO_4_ electrolytes. **f** Cycling performance of Zn||Zn symmetric cells with PAM-MFH^+^-ZnSO_4_ electrolytes at -10 °C, showing dendrite-free Zn plating and stable interfacial behavior [164]. Copyright 2025, Wiley

Based on this regulatory principle, Chen et al. introduced trehalose into PAM hydrogel systems, where hydroxyl-rich disaccharide molecules formed multiple hydrogen bonds with amide chains while partially replacing water molecules within the Zn^2+^ solvation sheath (Fig. 12a) [59]. This local solvent-environment reconstruction effectively reduced free-water activity and stabilized interfacial processes, enabling Zn||Zn symmetric cells to cycle stably for over 2400 h with a Coulombic efficiency of 98.8%. Building on this strategy, Guan et al. further introduced α-D-glucose (αDG), which not only participated in Zn^2+^ solvation structures but also regulated water-organization states through dynamic hydrogen-bond-network reconstruction (Fig. 12b) [157]. Partial substitution of water molecules within the primary Zn^2+^ solvation sheath and hydrogen-bond-network reorganization lowered Zn^2+^ desolvation barriers and suppressed proton activity, reducing the electrolyte freezing point to -55.3 °C while improving Zn^2+^ desolvation kinetics. Consequently, Zn||Zn cells maintained stable cycling for 2000 h at − 25 °C and 800 h even at − 40 °C, demonstrating stable ion transport and interfacial processes under ultralow-temperature conditions.

Further extending this mechanism, Sun et al. demonstrated that trehalose-modified hydrogels could dynamically regulate bound-water structures, thereby effectively suppressing hydrogen evolution and corrosion reactions [106]. The system maintained stable operation for 1200 h at − 15 °C and 700 h at 50 °C, while corresponding Zn||KVOH full cells retained 75.9% capacity after 500 cycles (Fig. 12c). Similarly, Cao et al. constructed a PAM-1,2-propanediol (1,2-PG) hydrogel system, where 1,2-PG formed strong hydrogen bonds with water molecules and disrupted original water-cluster structures, thereby lowering Zn^2+^ desolvation barriers and stabilizing interfacial processes (Fig. 12d) [180]. Meanwhile, redistribution of local liquid-state structures and hydrogen-bond states further enhanced configurational entropy regulation, helping maintain continuous ion transport under low-temperature conditions. This system eventually enabled in situ formation of an organic–inorganic bilayer SEI at the Zn interface and achieved stable cycling for over 3700 h at − 30 °C. In addition, Wang et al. employed metformin hydrochloride (MFHCl) to construct a synergistic system combining cationic coordination environments and dynamic hydrogen-bond networks [164]. In this system, MFH^+^ cations simultaneously interacted with PAM chains and SO_4_^2−^ anions, thereby reconstructing Zn^2+^ solvation structures and hydrogen-bond networks. Molecular dynamics simulations showed that the PAM-MFH^+^-ZnSO_4_ maintained temperature-adaptive hydrogen-bond networks and stable coordination structures from − 10 to 60 °C (Fig. 12e), indicating that local liquid-state structures and water-organization states could dynamically adapt to temperature variation. This dynamic local-state reconstruction mainly corresponds to configurational entropy regulation and helps maintain continuous ion transport and stable interfacial processes across wide-temperature ranges. Consequently, the corresponding Zn||Zn symmetric cells cycled stably for over 3600 h at − 10 °C with low polarization (Fig. 12f), demonstrating effective dendrite-free Zn deposition and stable low-temperature interfacial behavior.

Consequently, organic and bio-inspired cosolvent systems mainly regulate Zn^2+^ solvation behavior and interfacial processes through dynamic hydrogen-bond-network reconstruction and local solvent-environment regulation. Coordination regulation mainly weakens Zn^2+^–H_2_O interactions and lowers desolvation barriers, while entropy regulation mainly originates from dynamic reconstruction of hydrogen-bond networks and local liquid-state distributions, particularly corresponding to configurational entropy regulation. Therefore, such systems are especially beneficial for suppressing water-structure ordering at low temperatures and maintaining continuous ion transport, thereby enabling stable ion transport and interfacial electrochemical behavior across wide-temperature ranges.

#### Eutectic-Based Regulation

Within the C-E framework, eutectic systems represent a typical multicomponent solvent-environment regulation strategy for wide-temperature HPEs. Unlike conventional aqueous electrolytes dominated by water-rich solvation environments, eutectic systems introduce strongly coupled multicomponent hydrogen-bond networks and diversified liquid-state structures into the electrolyte matrix [12]. These eutectic environments can simultaneously regulate Zn^2+^ solvation structures, water activity and local transport states, thereby improving ion-transport continuity and interfacial stability across wide-temperature ranges [54]. Compared with conventional cosolvent systems, eutectic systems generally exhibit more pronounced entropy-regulation characteristics due to their intrinsically multicomponent liquid-state nature.

**Fig. 13:**
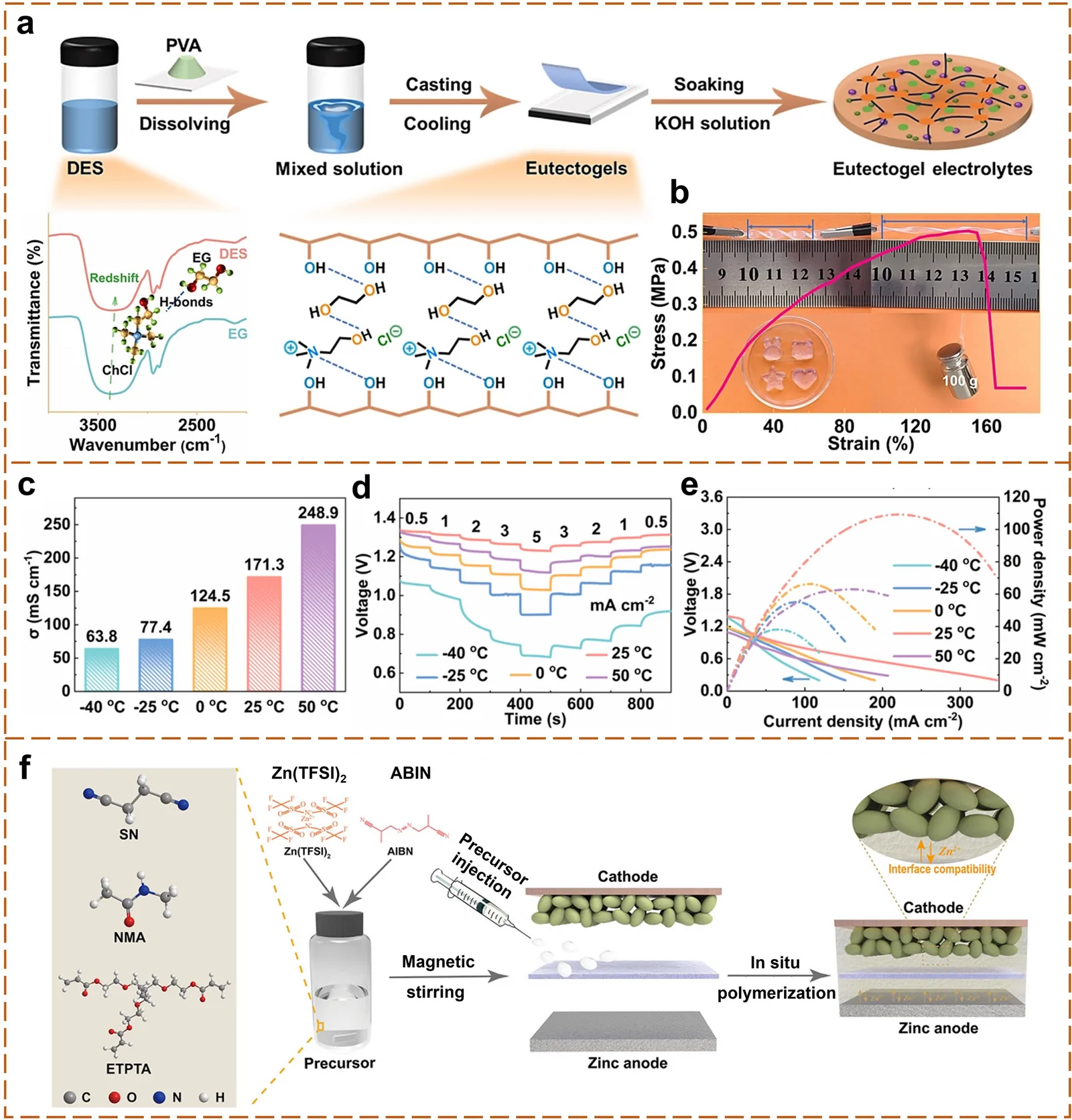
Eutectic-based hydrogel electrolyte strategies for wide-temperature operation.** a** Schematic illustration of the preparation process of the CEP electrolyte. **b** Stress–strain curves and compressive elasticity of the CEP electrolyte. **c** Temperature-dependent ionic conductivity of the CEP electrolyte. **d** Discharge curves under various current densities (0.5–5 mA cm^−2^). **e** Discharge polarization and power-density curves of the CEP electrolyte at different temperatures [54]. Copyright 2025, Wiley. **f** Schematic illustration of the preparation process and thermal stability of the PSNE electrolyte [181]. Copyright 2024, Springer Nature

Based on this regulatory principle, Jia et al. developed a ChCl/EG-PVA eutectic hydrogel electrolyte (CEP), in which choline chloride (ChCl) and ethylene glycol (EG) formed a compact and thermally stable hydrogen-bond network within the PVA framework (Fig. 13a) [54]. The eutectic–polymer integration reinforced network stability while maintaining continuous ion-transport pathways. As shown in Fig. 13b, the CEP electrolyte maintained excellent elasticity and structural integrity even under compressive stress exceeding 0.5 MPa, indicating dynamic balance between structural adaptability and network stability. Meanwhile, the eutectic hydrogen-bond network continuously reorganized local liquid-state structures and ion pathways under temperature variation, thereby maintaining stable ion transport and interfacial contact. The wide-temperature electrochemical behavior of the CEP electrolyte further demonstrated this C-E coupling effect. Its ionic conductivity increased significantly from 63.8 to 254.5 mS cm^−1^ as temperature increased from − 40 to 50 °C, following Arrhenius-type thermally activated Zn^2+^ migration behavior. In contrast, the reference HP hydrogel exhibited much lower ionic conductivity over the same temperature range (16.3 mS cm^−1^ at − 40 °C and 38.1 mS cm^−1^ at 50 °C), confirming the superior transport adaptability of eutectic systems (Fig. 13c). When applied in flexible Zn-air batteries, the CEP electrolyte enabled stable and reversible operation across the entire temperature range from − 40 to 50 °C (Fig. 13d, e), delivering power densities of 38.2 and 63.2 mW cm^−2^ at − 40 and 50 °C, respectively. These values exceeded those of most previously reported low-temperature flexible Zn-air batteries, indicating that eutectic systems can simultaneously stabilize low-temperature ion transport and high-temperature interfacial processes. Building on this strategy, Huang et al. further developed a ternary eutectic-based polymerized supramolecular network electrolyte (PSNE) through in situ polymerization of a deep-eutectic solvent (DES) system using ethoxylated trimethylpropane triacrylate (ETPTA) as the cross-linker (Fig. 13f) [181]. In this system, eutectic components provided multiligand coordination environments that reconstructed Zn^2+^ solvation structures and reduced desolvation barriers. Meanwhile, coexistence of multicomponent liquid states and coupled hydrogen-bond networks redistributed local ion configurations and transport pathways, corresponding mainly to mixing entropy and configurational entropy regulation. These effects further reduced concentration gradients and transport anisotropy, thereby improving ion-transport uniformity and stabilizing interfacial processes during long-term cycling. As a result, the PSNE system achieved stable electrochemical operation over wide-temperature ranges with improved interfacial stability and transport kinetics.

Overall, eutectic systems mainly regulate Zn^2+^ transport and interfacial processes through coupled solvation reconstruction and multicomponent liquid-state regulation. Coordination regulation weakens Zn^2+^–H_2_O interactions and stabilizes Zn^2+^ solvation structures, while entropy regulation mainly originates from multicomponent eutectic environments and diversified liquid-state distributions, particularly corresponding to mixing entropy regulation. Therefore, eutectic systems are especially effective for suppressing low-temperature crystallization, maintaining continuous ion transport and stabilizing interfacial electrochemical processes under wide-temperature conditions.

### Filler Regulation

Within the C-E framework, filler regulation represents an important strategy that extends molecular-scale solvation regulation toward mesoscale transport regulation, with its core role being to bridge bulk ion-transport behavior and interfacial electrochemical processes [102, 182]. Unlike polymer systems, salt systems and cosolvent systems that mainly regulate local Zn^2+^ solvation environments, filler regulation primarily focuses on regulating Zn^2+^ migration behavior and local transport states through spatial confinement, interfacial interactions and transport-pathway reconstruction. Consequently, the regulatory mechanism of filler systems is more strongly associated with entropy regulation, particularly topological entropy regulation. Meanwhile, active surface sites, defect structures and functional groups on fillers can also participate in local Zn^2+^ solvation regulation, thereby contributing certain coordination-regulation effects. Specifically, Lewis-acidic sites, oxygen vacancies, functional groups and open-framework structures within fillers can interact with Zn^2+^ ions or anions, thereby regulating local Zn^2+^ coordination environments, weakening Zn^2+^–H_2_O interactions and lowering desolvation barriers. Meanwhile, porous structures, nanofluidic channels and spatial confinement environments further redistribute local ionic states and ion-transport pathways, thereby reducing local transport anisotropy and alleviating ion-concentration gradients. These effects help maintain continuous Zn^2+^ transport and homogeneous interfacial flux. Through such combined regulation, filler regulation can simultaneously improve ion-transport continuity, interfacial stability and Zn deposition uniformity across wide-temperature ranges.

**Fig. 14:**
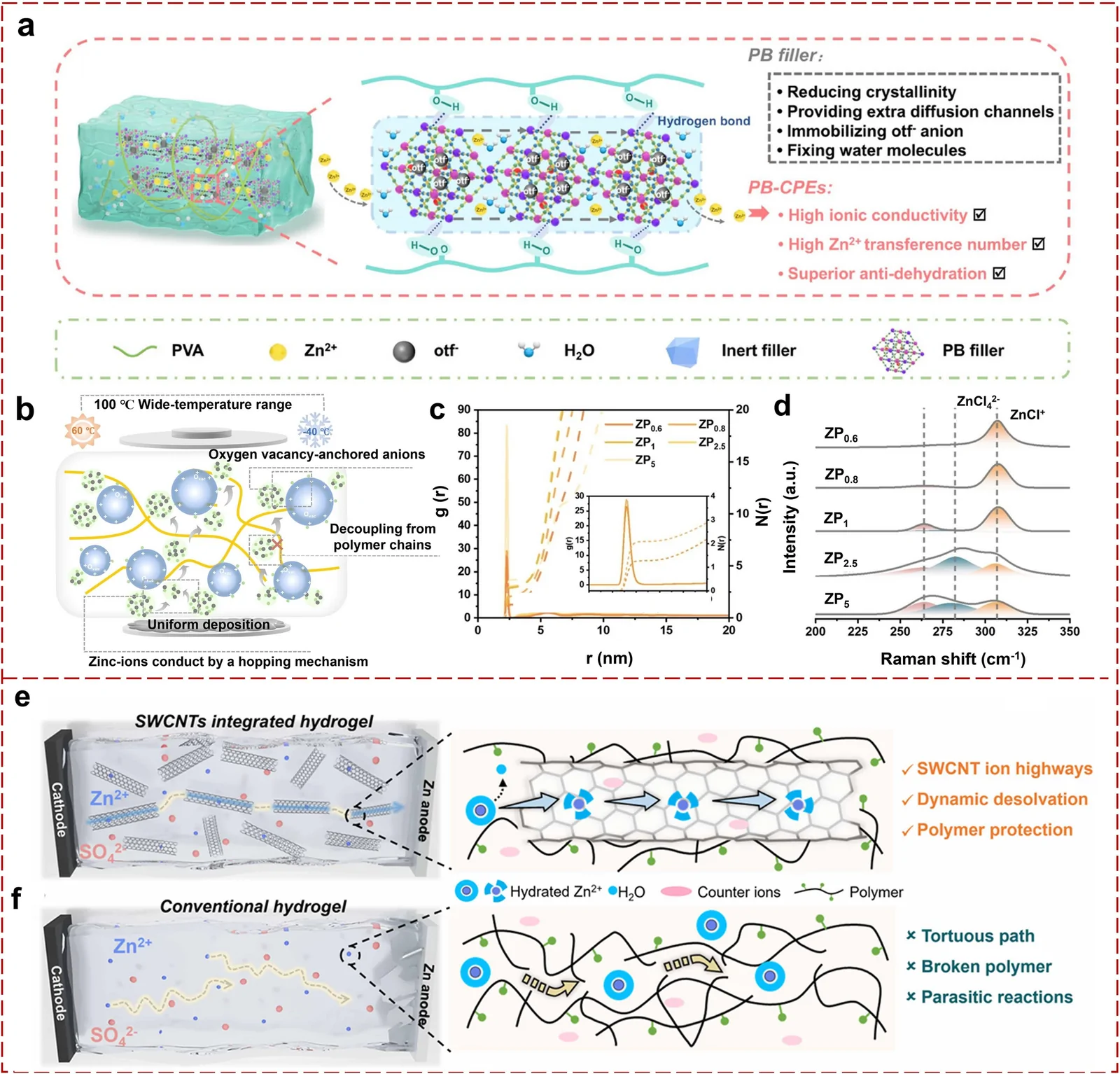
Filler regulation strategies for wide-temperature hydrogel electrolytes**. a** Schematic illustration of the Zn^2+^ conduction mechanism in PB-CPE HPEs [119]. Copyright 2025, Wiley. **b** Interaction mechanism in ZP_0.8_@Nb_2_O_5-x_ hydrogel electrolytes, where oxygen vacancies regulate ionic environments and anchor anions. **c** Radial distribution functions and coordination numbers of ZP_x_ electrolytes, confirming Zn^2+^ solvation reconstruction. **d** Raman spectra of PAN and ZPₓ electrolytes, revealing C-E modulation after Nb_2_O_5-x_ incorporation [183]. Copyright 2024, Wiley. **e** Schematic illustration of fast ion transport through SWCNT channels in CPAM. **f** Schematic illustration of tortuous ion transport in conventional PAM [184]. Copyright 2025, American Association for the Advancement of Science

Based on this regulatory principle, Li et al. incorporated Prussian blue analogue (PBA) frameworks into a PVA matrix to construct coordination-enhanced hydrogel electrolytes (Fig. 14a) [119]. The Fe-centered Lewis-acidic sites promote zinc-salt dissociation and participate in Zn^2+^ coordination, thereby partially replacing water molecules within the primary solvation sheath and lowering desolvation barriers. Meanwhile, the open-framework structure redistributes local ionic states and ion-transport pathways through spatial confinement, thereby alleviating local ion aggregation and maintaining continuous ion transport. Consequently, the system exhibits high ionic conductivity and a Zn^2+^ transference number of 0.63. Corresponding Zn||Zn symmetric cells maintain stable cycling for over 1600 h at 50 °C, demonstrating effective stabilization of ion transport and interfacial processes.

Building on this strategy, Liang et al. further introduced oxygen-defective Nb_2_O_5-x_ nanofillers into polyacrylonitrile-ZnCl_2_ hydrogel systems to further couple coordination regulation with mesoscale transport regulation (Fig. 14b) [183]. Oxygen vacancies anchor Cl^−^ anions and reconstruct Zn^2+^ solvation structures, thereby promoting formation of ZnCl^+^ intermediates with reduced desolvation barriers. Meanwhile, defect-induced spatially heterogeneous ionic environments further redistribute local ionic states and transport pathways. Molecular dynamics simulations and Raman analyses (Fig. 14c, d) further confirmed reconstruction of Zn–O and Zn–Cl coordination structures, thereby enabling faster Zn^2+^ migration and lower polarization. As a result, the system achieves both homogeneous Zn deposition and stable interfacial processes under low- and high-temperature conditions.

Beyond crystalline framework fillers, carbon-based nanofluidic fillers can further construct continuous ion-transport pathways within hydrogel systems. Lin et al. developed a single-walled carbon nanotubes (SWCNTs)-integrated polyacrylamide hydrogel (CPAM), in which uniformly distributed SWCNTs serve as continuous ion-transport channels (Fig. 14e) [184]. Compared with conventional hydrogels, where Zn^2+^ transport proceeds through tortuous polymer pathways (Fig. 14f), SWCNT nanofluidic channels redistribute ion-transport pathways and reduce local transport anisotropy, thereby maintaining more continuous and efficient Zn^2+^ migration. Meanwhile, confined environments can induce partial Zn^2+^ desolvation, thereby weakening Zn^2+^–H_2_O interactions and lowering desolvation barriers. Consequently, the system exhibits high ionic conductivity (~ 30 mS cm^−1^) and enables stable Zn||Zn cycling for over 7000 h. Notably, the system still maintains stable operation at − 15 °C and retains approximately 80% capacity after 2000 cycles at a high current density of 40 A g^−1^, demonstrating excellent ion transport and interfacial stability under both low-temperature and high-rate conditions. In addition, Chen et al. further incorporated phytic-acid-functionalized Fe-based metal–organic frameworks (MOFs, MIL-88A) into a PAM matrix [163]. Phosphate groups participate in Zn^2+^ coordination, thereby regulating Zn^2+^ solvation structures and lowering desolvation barriers, while the MOF framework further reconstructs local ionic states and ion-transport pathways through spatial confinement. Meanwhile, hydrogen-bond interactions between phosphate groups and polymer chains further stabilize the network structure. This multiscale synergistic regulation ultimately enables continuous ion transport and stable interfacial processes, while exhibiting stable electrochemical performance and good mechanical flexibility under various operating conditions.

As a result, filler regulation mainly regulates Zn^2+^ transport and interfacial processes through spatial confinement and mesoscale transport-pathway reconstruction, and its regulatory mechanism is therefore more strongly associated with entropy regulation, particularly topological entropy regulation. Porous structures, nanofluidic channels and spatially heterogeneous local transport environments constructed by fillers redistribute local ionic states and ion-migration pathways, thereby alleviating local transport anisotropy and maintaining continuous ion transport. Meanwhile, active surface sites and defect structures on fillers can also participate in local Zn^2+^ solvation regulation to some extent, thereby weakening Zn^2+^–H_2_O interactions and lowering desolvation barriers. Therefore, filler regulation is particularly beneficial for stabilizing ion-transport continuity and interfacial electrochemical behavior across wide-temperature ranges, providing an important strategy for designing high-performance wide-temperature hydrogel electrolytes.

### Multifunctional Integrated Design

In practical wide-temperature HPE systems, Zn^2+^ solvation structures, ion-transport behavior, interfacial stability and macroscopic structural adaptability are typically not governed by a single regulatory mechanism, but instead arise from the synergistic coupling of coordination regulation and entropy regulation across different structural scales [185, 186]. Therefore, compared with single-component or single-scale regulation strategies, integrated C-E regulation focuses on the simultaneous regulation of local solvation environments, mesoscale transport states, interfacial reaction processes and macroscopic mechanical behaviors within a unified system, thereby enabling stable ion transport and interfacial electrochemical processes under wide-temperature conditions. Within the C-E framework, such integrated regulation is not a simple superposition of coordination regulation and entropy regulation, but rather an intrinsic coupling of the two across different structural levels. Specifically, coordination regulation reconstructs local Zn^2+^ solvation environments, weakens Zn^2+^–H_2_O interactions and lowers desolvation barriers, thereby providing the thermodynamic basis for ion migration and interfacial reactions. Meanwhile, redistribution of local ionic states, transport-pathway evolution and structural heterogeneity induced by solvation reconstruction further reflect entropy-regulation effects, thereby maintaining continuous ion transport and stable interfacial processes under temperature variation. Therefore, in practical wide-temperature systems, coordination regulation and entropy regulation usually exist as cross-scale synergistic regulation rather than independent parallel mechanisms.

**Fig. 15:**
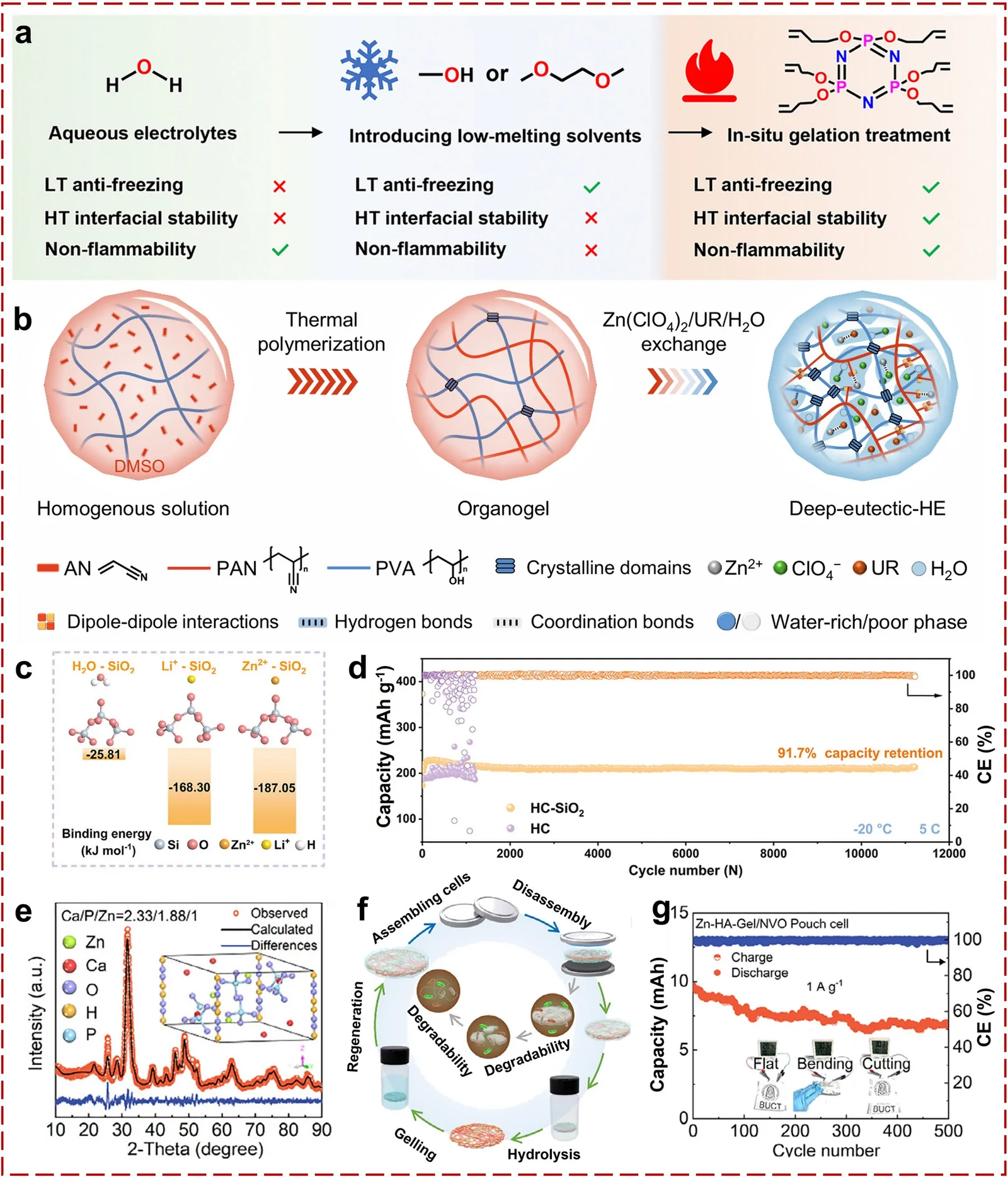
Multifunctional integrated regulation strategies for wide-temperature hydrogel electrolytes.** a** Design concept of polyphosphonitrile-based gel electrolyte for ultrawide-temperature Zn batteries [187]. Copyright 2025, Royal Society of Chemistry. **b** Preparation of nanophase-separated deep-eutectic hydrogel via solvent-exchange process [188]. Copyright 2026, Springer Nature. **c** DFT-calculated adsorption energies of Zn^2+^ and H_2_O on SiO_2_ surfaces in the Zn(OAc)_2_-LiCl-SiO_2_ composite electrolyte. **d** Electrochemical performance of the Zn(OAc)_2_-LiCl-SiO_2_ system at -20 °C [189]. Copyright 2025, Wiley. **e** Lattice structure and preferred (002) orientation of Zn deposition on Zn-HA frameworks. **f** Schematic illustration of hot-water recycling of the Zn-HA-gelatin composite electrolyte. **g** Stable operation of flexible pouch cells under bending, puncture and cutting conditions, demonstrating excellent mechanical adaptability [117]. Copyright 2025, American Chemical Society

Based on this regulatory concept, Kang et al. constructed a polyphosphonitrile-derivative gel electrolyte through in situ gelation of low-melting-point cosolvents and butenoxycyclotriphosphazene (BCPN) monomers (Fig. 15a) [187]. In this system, low-melting-point cosolvents partially replace water molecules within the Zn^2+^ solvation sheath and disrupt hydrogen-bond networks, thereby reducing water activity and improving low-temperature ion transport. Meanwhile, the in situ polymerized BCPN network further suppresses electrolyte leakage and flammability, while residual BCPN participates in formation of a thermally stable SEI at elevated temperatures. Consequently, both low-temperature transport stability and high-temperature interfacial stability originate from a unified solvation-interfacial regulation process. The system ultimately achieved a Coulombic efficiency of 99.75%, stable Zn||Zn cycling over 10,500 h and ultrawide-temperature operation from − 70 to 80 °C. Furthermore, Jin et al. developed a nanophase-separated deep-eutectic hydrogel electrolyte through a solvent-exchange-induced structural evolution process (Fig. 15b) [188]. The resulting system consisted of interwoven hydrophilic PVA domains, hydrophobic PAN phases, crystalline regions and hydrated eutectic components. Cyano groups within PAN participated in Zn^2+^ coordination and formed continuous ion-transport channels, thereby lowering desolvation barriers. Meanwhile, hydrogen-bond-network reconstruction further redistributed water molecules among heterogeneous domains, thereby generating diversified local ion-transport states and structural environments. Such cross-scale structural heterogeneity enabled continuous Zn^2+^ transport within dynamically evolving structural environments, thereby preventing transport collapse under temperature variation. Consequently, the system achieved an ionic conductivity of 28.2 mS cm^−1^, a Zn^2+^ transference number of 0.65 and stable cycling of Zn||I_2_ batteries over 36,000 cycles from − 40 to 80 °C.

At an intermediate scale that links molecular coordination with mesoscale network regulation, Yang et al. designed a filler-salt-network integrated hydrogel electrolyte based on a Zn(OAc)_2_-LiCl-SiO_2_ system [189]. Strong Li^+^–Cl^−^ association redistributed anion-coordination states and weakened Zn^2+^–Cl^−^ interactions, thereby lowering Zn^2+^ desolvation barriers. Meanwhile, hydroxyl-rich SiO_2_ nanoparticles further introduced spatially distributed multisite interaction environments. DFT calculations showed negative adsorption energies for both Zn^2+^ ions and H_2_O molecules on SiO_2_ surfaces (Fig. 15c), indicating simultaneous perturbation of Zn^2+^ solvation structures and hydrogen-bond networks. In this process, Zn^2+^ ions migrated not through a single dominant configuration, but continuously among multiple dynamically accessible local states, thereby avoiding localized transport concentration and maintaining homogeneous Zn^2+^ flux. As a result, the system still delivered a capacity of 210 mAh g^−1^ at − 20 °C and maintained 91.7% capacity retention after 11,000 cycles (Fig. 15d).

At the macroscopic structural level, Liu et al. achieved cross-scale C-E synergistic regulation by incorporating Zn-doped hydroxyapatite (Zn-HA) into a gelatin matrix [117]. The Zn-HA lattice provided continuous Zn^2+^ migration channels and preferential coordination along the (002) crystal facet (Fig. 15e), while the gelatin network contributed structural flexibility and stress-dissipation capability, thereby enabling rigid-flexible coupling and suppressing dendrite penetration. In addition, the composite system could be recycled into Zn-HA films and gelatin solution through hot-water hydrolysis (Fig. 15f), demonstrating synergistic integration of electrochemical performance and sustainability. Flexible pouch cells assembled with Zn-HA-Gel electrolytes could still stably power digital thermometers under bending, puncture and cutting conditions (Fig. 15g), demonstrating excellent structural adaptability and safety across wide-temperature ranges.

Overall, integrated C-E regulation demonstrates that Zn^2+^ solvation reconstruction, structural heterogeneity, ion-transport regulation and interfacial stabilization can be synergistically coupled across multiple structural scales within a unified system. In such systems, coordination regulation provides the thermodynamic basis for Zn^2+^ migration and interfacial reactions, while entropy regulation maintains continuous ion transport and structural adaptability through redistribution of local states and evolution of transport pathways. Therefore, these strategies simultaneously enable homogeneous Zn^2+^ flux, stable interfacial chemistry, excellent mechanical stability and wide-temperature operation, providing a unified multiscale design framework for advanced wide-temperature hydrogel electrolytes.

Following the above discussion, Table 3 summarizes representative wide-temperature HPEs and reveals how different design strategies lead to distinct performance advantages, particularly in temperature adaptability. These comparisons further reflect the practical implementation of C-E regulation through different material-design approaches.

**Table 3:** Summary and mechanistic comparison of C-E design strategies for wide-temperature HPEs (continued)

Primary Design strategy	Hydrogel substrate	Electrolyte formulation	Temperature window (°C)	Mechanical property (MPa)	Ionic conductivity (mS cm^−1^)	Zn||Zn performance	Full-cell performance	Ref.
Polymer System Regulation	PUA	3 M Zn(OTf)_2_ + Tf-im	RT-60	RT, tensile 0.34, compressive 3.18	RT, 31.6	60 ℃, 0.5 mA cm^−2^, 0.5 mAh cm^−2^, 710 h	Zn||VO_2_, 60 ℃,1A g^−1^,400 cycles,60.2%	[17]
	PAPTMA	2 M ZnSO_4_	− 75	RT, tensile 0.096; adhesion 0.050	RT, 28.7	RT, 1 mA cm^−2^, 1 mAh cm^−2^,6000 h	Zn||ZVO, 60 ℃, 1A g^−1^,263 mAh g^−1^,100 cycles,77.5%	[79]
	PAM	2 M Zn(OTf)_2_ + T3	− 100	RT, tensile 0.065; compressive 0.842	− 20 °C, 2.14; 80 °C, 19.36	− 20 °C, 1 mA cm^−2^, 1 mAh cm^−2^, 7600 h; 40 °C, 1 mA cm^−2^, 1 mAh cm-2, 1200 h	Zn||NVO, -20 °C, 5400 cycles; 80 °C, 153.0 mAh g^−1^, 300 cycles	[169]
	PDOL-based gel	2 M Zn(OTF)_2_	− 70-RT	/	− 70 °C,0.36	− 70 ℃, 0.1 mA cm^−2^,0.1 mAh cm^−2^, 1000 h	Zn||ZVO, − 40 ℃,0.1 A g^−1^, 100 cycles	[165]
	PMEM/HEAA	2 M Zn(OTf)_2_, 35 wt% H_2_O	RT-90	RT, tensile 0.087	RT,3.9	90 °C, 1 mA cm^−2^, 1 mAh cm^−2^, 1000 h	Zn||ZVO, 90 °C, 1 A g^−1^, @750 cycles, 90%	[149]
	PAM/PEG/PAA Na/SA/SF	2 M Zn(ClO_4_)_2_	− 80–20	/	20 °C, 33.0, − 80 °C, 1.2	− 20 °C, 1 mA cm^−2^, 1 mAh cm^−2^, 2500 h; − 80 °C, reversible	− 50 °C, 0.5 A g^−1^, 400 cycles; − 80 °C, reversible operation	[137]
	PVA/PA	2 M ZnSO_4_	− 80–60	RT, tensile 1.31	30 °C, 6.05	RT, 0.5 mA cm^−2^, 0.5 mAh cm^−2^, 2880 h	Zn||VO_2_, RT, 1.0 A g^−1^, @400 cycles, 86.5% retention	[190]
	CZHE	3 M Zn(ClO_4_)_2_ + 40 wt% maltose	− 60–25	/	25 °C, 33.1	RT, 3 mA cm^−2^, 3 mAh cm^−2^, 1000 h	Zn||PANI, − 60 °C, 3 A g^−1^, @300 cycles, 99.9%	[172]
Salt System Regulation	PAM	3 M Zn(BF_4_)_2_	− 70–25	/	− 70 °C, 2.38	/	Zn||PANI, -70 °C, 0.05 A g^−1^, 48.5 mAh g^−1^, 100 cycles, 99.9%	[174]
Salt system regulation	PAM	3 M ZnCl_2_ + 6 M LiCl	− 50–25	/	− 50 °C, 1.14	/	Zn||LFP, − 50 °C, 0.5 A g^−1^, 96.5 mAh g^−1^,2000 cycles	[191]
	PAM	Zn(Ac)_2_/KAc	− 2–100	RT, tensile 0.030; adhesion 0.015	RT, 34.7; − 40 °C, 1.5	− 20 °C, 0.5 mA cm^−2^, 0.25 mAh cm^−2^, 1300 h	Zn||PANI, − 20 °C, 0.5 A g^−1^, 1100 cycles, 81.4%;	[53]
	PVA	4.6 M KAc + 0.51 M Zn(Ac)_2_	− 77–25	RT, tensile 15.6	RT, 49.8	− 20 °C, 0.5 mA cm^−2^, 0.1 mAh cm^−2^, 1200 h	Zn||PANI, − 30 °C, 0.5 A g^−1^, 1000 cycles, 95%	[52]
Cosolvent regulation	PAM	2 M Zn(OTf)_2_, + 1,2-PG	-30-RT	/	RT, 29.6	− 30 ℃, 1 mA cm^−2^, 1 mAh cm^−2^, > 3780 h	Zn||VOH, − 30 ℃, 0.5 A g^−1^, 97 mAh g^−1^,8000 cycles	[180]
	PAM	2 M ZnSO_4_ + trehalose	− 15–50	RT, tensile 0.055	RT, 19.3	− 15 °C, 1 mA cm^−2^, 1 mAh cm^−2^, > 1200 h; 50 °C, 1 mA cm^−2^, 1 mAh cm^-2^, 700 h	Zn||KVOH, -15 °C, 3 A g^−1^, 2000 cycles, 92.6%; 50 °C, A g^−1^, 1000 cycles, 58.9%	[59]
Filler regulation	PAM	2 M ZnSO_4_ + SWCNT	− 15–25	RT, tensile 0.736	RT, 30.3	RT, 1 mA cm^−2^, 0.5 mAh cm^−2^, 7030 h;	Zn||ZVO, -15 °C, 0.5 A g^−1^, 80 cycles, 82%	[184]
	PAN	ZnCl_2_ + Nb_2_O_5-x_	− 40–60	RT, tensile 2	RT, 2.02	− 40 °C, 0.1 mA cm^−2^, 0.1 mAh cm^−2^, 2400 h	Zn||PANI, -20 °C, 0.5 A g^−1^, 650 cycles; 60 °C, 233 mAh g^−1^	[183]
Multifunctional integrated design	PAM	2 M ZnSO_4_ + MFHCl	− 10–60	/	RT, 25.4	− 10 °C, 1 mA cm^−2^, 1 mAh cm^−2^, 3600 h; 60 °C, 1 mA cm^−2^, 1 mAh cm^-2^, 900 h	Zn||NHVO, − 10 °C, 0.1 A g^−1^, 200 cycles; 60 °C, 2 A g^−1^, 160 cycles	[164]
	PVA	1 M Zn(OTf)_2_, + ZnHCF	RT-50	RT, tensile 2.67	RT, 16.3	50 °C, 0.5 mA cm^−2^, 0.5 mAh cm^−2^,1600 h	Zn||NVO, 50 °C, 1 A g^−1^, 1600 cycles, 65%	[119]
Multifunctional integrated design	BCPN	1 M Zn(OTf)_2_ + MeOH	− 70–80	/	− 70 °C, 1.05	− 60 °C, 0.1 mA cm^−2^, 0.1 mAh cm^−2^, 6300 h	Zn||PANI, -70 °C, 0.1 g^−1^, 95.6 mAh g^−1^,100 cycles; 80 °C, 1.5 A g^−1^, 237.1 mAh g^−1^, @@100 cycles, 98.7%	[187]
	PAM/DMAPS	2 M Zn(ClO_4_)_2_ + 4 M LiClO_4_	− 40–25	RT, tensile 0.026	− 40, 7.75	− 20 °C, 1 mA cm^−2^, 2 mAh cm^−2^, 2100 h	Zn||VO_2_-V_2_O_5_, − 40 °C, 1 A g^−1^, 5000 cycles, 83.7%	[122]
	PVA/PAN	Zn(ClO_4_)_2_ + UR	− 40–80	RT, tensile 4.09	RT, 28.2	− 40 °C, 0.5 mA cm^−2^, 0.5 mAh cm^−2^,1000 h; 80 °C, 0.5 mA cm^−2^, 0.5 mAh cm^−2^, 800 h	Zn||I_2_, − 40 °C, 5 A g^−1^, 95.6 mAh g^−1^, 36,000 cycles,92.3%; 80 °C, 5 A g^−1^, 119.57 mAh g^−1^, 36,000 cycles, 81.0%;	[188]
	HC-SiO_2_	1 M Zn(OAc)_2_ + 21 M LiCl	− 60–80	/	− 60, 0.354	− 40 °C, 0.2 mA cm^−2^, 0.2 mAh cm^−2^, 600 h;	Zn||I_2_, − 20 °C, 5 °C, 210 mAh g^−1^, 11,000 cycles, 91.7%	[189]

RT denotes room temperature

Collectively, although these design strategies are implemented through different material approaches, they collectively regulate Zn^2+^ solvation structures, local transport states and interfacial electrochemical processes within the unified C-E framework. In practical wide-temperature HPE systems, coordination regulation and entropy regulation are intrinsically coupled rather than independently operating mechanisms. Specifically, reconstruction of Zn^2+^ coordination environments not only weakens Zn^2+^–H_2_O interactions and lowers desolvation barriers, but also further induces redistribution of local ionic states, transport pathways and structural environments across multiple structural scales. This cross-scale coupling enables continuous ion transport, homogeneous Zn^2+^ flux, stable interfacial chemistry and adaptive structural stability under temperature variation. Therefore, the electrochemical performance of wide-temperature HPEs fundamentally depends on how effectively solvation regulation, transport-state redistribution and interfacial stabilization can be synergistically integrated within different material-design strategies.

## Advanced Characterization and Computational Tools for C-E Regulated HPEs

Although the C-E framework provides a unified description of hydrogel electrolytes by linking coordination environments with the distribution of accessible states, its experimental validation remains challenging due to the coupled evolution of interaction structures and transport behavior across multiple scales [94, 192]. Conventional ex situ characterization techniques provide only static information and are insufficient to capture the dynamic evolution of coordination environments and state distributions at the electrolyte–electrode interface. Therefore, it has become necessary to combine multiscale computational modeling with in situ and operando characterizations. Such an integrated approach helps relate coordination environments to these state distributions and track their evolution under working conditions, providing a basis for understanding the relationship between microscopic regulation and macroscopic electrochemical behavior [114].

### Computational Screening and Mechanistic Modeling

**Fig. 16:**
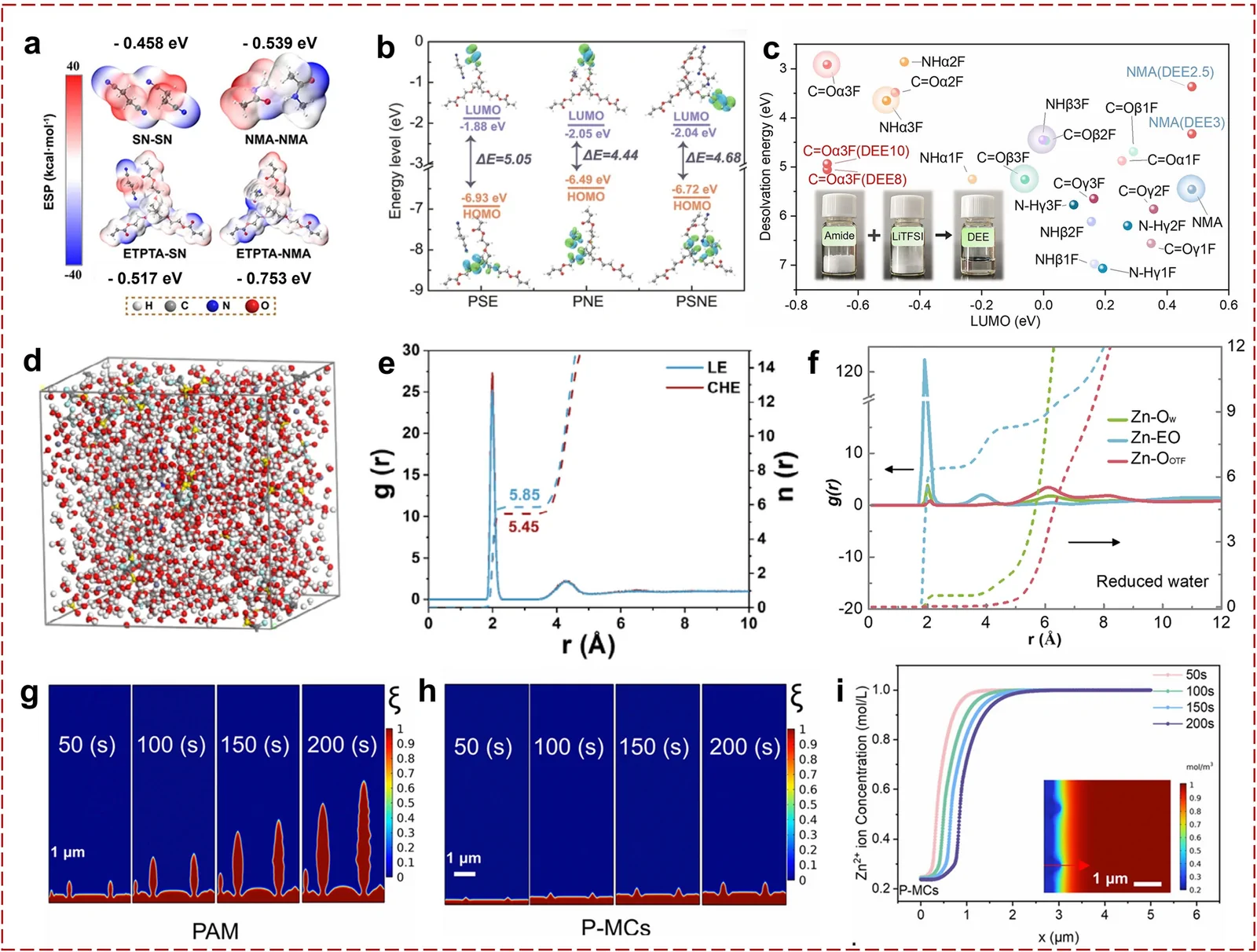
Computational modeling strategies for C-E regulated hydrogel electrolytes.** a** Electrostatic potential maps and binding energies of eutectic components, revealing anchoring interactions among Zn^2+^, SN and NMA molecules. **b** Molecular orbital energy levels of PSN, PNE and PSNE electrolytes, showing reduced HOMO levels and widened band gaps for improved oxidative stability [181]. Copyright 2025, Springer Nature. **c** DFT-screened LUMO energies and desolvation barriers of amide-based ligands, correlating electronic structure with Zn^2+^ coordination stability [193]. Copyright 2025, American Chemical Society. **d** MD snapshot of the CHE hydrogel showing spatial distribution of coordinated Zn^2+^ and water molecules. **e** Radial distribution functions confirming reduced Zn–O(H_2_O) coordination in hydrogels [171]. Copyright 2024, Wiley. **f** RDFs and coordination numbers of Zn^2+^ with H_2_O, OTF^−^and polymer oxygen under various hydration levels, illustrating entropy-driven solvation transitions [149]. Copyright 2025, Elsevier. **g** COMSOL simulated Zn deposition in PAM hydrogel showing Zn^2+^ accumulation at dendrite tips. **h** Electric-field and Zn^2+^ concentration profiles in P-MCs hydrogels, demonstrating field redistribution and planar Zn deposition. **i** Time-resolved Zn^2+^ concentration evolution at dendrite tips, confirming that the P-MC network homogenizes electric fields and suppresses dendrite growth [81]. Copyright 2024, Royal Society of Chemistry

At the molecular level, DFT provides quantitative descriptors of coordination environments by evaluating Zn^2+^-ligand binding energies, desolvation barriers and electronic structures [192]. These calculations provide insight into coordination regulation by revealing how different ligands modify Zn^2+^ solvation structures and influence interfacial reaction energetics. For example, Huang et al. developed a ternary eutectic system of N-methylacetamide (NMA), succinonitrile (SN) and Zn(TFSI)_2_, followed by in situ polymerization to form a eutectic gel [181]. DFT calculations revealed strengthened solvent–solvent and polymer–solvent interactions, which stabilized coordination environments, suppressed side reactions and facilitated Zn^2+^ migration (Fig. 16a). In addition, molecular orbital analysis shows reduced HOMO levels and widened band gaps, indicating enhanced electrochemical stability (Fig. 16b). Descriptor-based screening further correlates ligand electronic structures with Zn^2+^ desolvation barriers, enabling rational selection of coordination environments (Fig. 16c) [193].

At the mesoscopic scale, molecular dynamics (MD) simulations provide quantitative insight into entropy regulation by resolving the distribution of coordination configurations and interaction structures. Specifically, coordination number (CN), radial distribution functions (RDF) and solvation-cluster statistics describe the diversity and population of Zn^2+^ coordination environments, which are closely related to configurational entropy. In multicomponent systems, the coexistence of different ionic species and solvent molecules contributes to mixing entropy, while variations in ion–ion correlations and aggregation behavior can be used to describe ion entropy. Meanwhile, the evolution of polymer-network connectivity and ion-transport pathways is associated with topological entropy. For instance, MD snapshots and RDF analysis reveal the spatial distribution of Zn^2+^ coordination structures and reduced water coordination in hydrogels (Fig. 16d, e) [171]. Under low-water or multicomponent conditions, diversified Zn^2+^ coordination with solvent molecules, anions and polymer functional groups further reflects the redistribution of solvation structures (Fig. 16f) [149]. These results collectively indicate increased mixing entropy and ion entropy, which facilitate Zn^2+^ transport, particularly under low-temperature conditions.

At the device scale, finite-element simulations (e.g., COMSOL) visualize ion transport and interfacial flux distribution under operating conditions. These simulations establish a direct link between microscopic coordination/entropy regulation and macroscopic electrochemical behavior by showing how local conductivity, ion-concentration gradients and electric-field distribution influence Zn deposition morphology. For example, simulations show that Zn^2+^ tends to accumulate at dendrite tips in conventional hydrogels (Fig. 16g), whereas composite systems redistribute local electric fields and Zn^2+^ concentration, leading to more uniform deposition (Fig. 16h, i) [81].

Collectively, multiscale computational approaches provide a coherent validation pathway for the C-E framework. DFT provides molecular-level insights into coordination regulation by evaluating Zn^2+^-ligand binding energies, desolvation barriers and electronic structures. Meanwhile, MD provides quantitative descriptors for entropy regulation, particularly for configurational entropy, and offers structural indicators for mixing, ion and topological entropy. Continuum simulations further translate these features into macroscopic transport behavior. This integrated strategy enables a mechanistic understanding of how coordination regulation and entropy regulation are simultaneously realized in HPEs and provides guidance for rational electrolyte design across wide-temperature conditions.

### In Situ and Operando Spectroscopy

At the molecular and interfacial levels, in situ and operando spectroscopic techniques provide time-resolved insights into the evolution of coordination environments, hydrogen-bond configurations and interfacial reactions in HPEs [80]. These techniques enable direct observation of how coordination regulation and the distribution of accessible structural states evolve under working conditions, thereby offering experimental support for the C-E framework. In this context, direct experimental probing of liquid structures provides critical insight into interfacial processes in hydrogel electrolytes. For instance, combined spectroscopic and scattering techniques have been employed to resolve the local environments of water molecules and Zn^2+^ solvation structures in water-in-salt and ionic liquid systems, offering high-resolution information on hydrogen-bond networks and solvation configurations that are closely related to interfacial regulation [194].

**Fig. 17:**
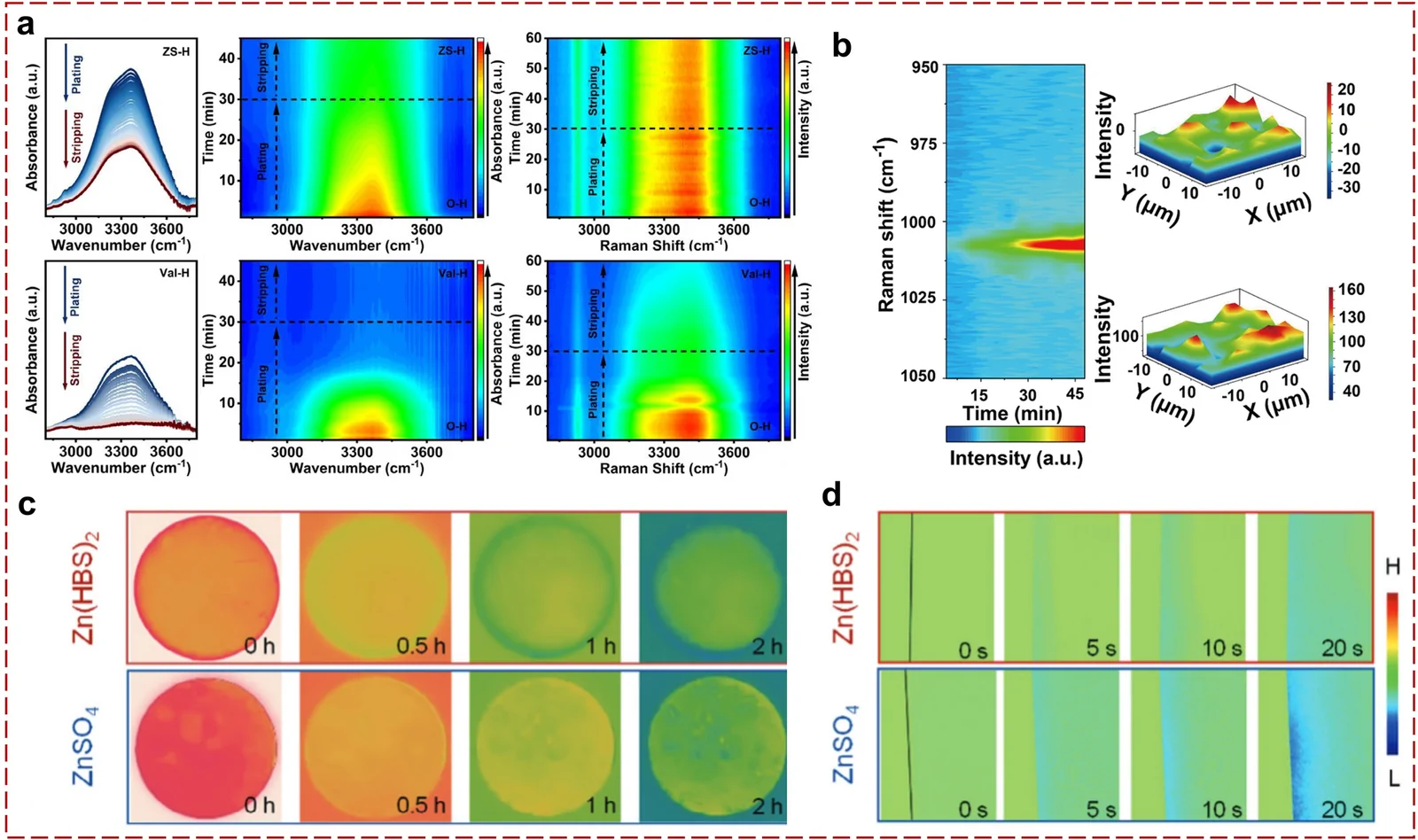
In situ and operando characterization strategies.** a** In situ FTIR spectra and contour plots at the Zn/electrolyte interface in ZS-H and Val-H electrolytes, together with Raman spectra revealing interfacial H_2_O evolution [80]. Copyright 2025, Wiley. **b** In situ Raman spectra and mapping of ZnSO_4_ in the SA/EIDC gel electrolyte before and after deposition, showing coordination-regulated Zn^2+^ migration [112]. Copyright 2025, Wiley. **c** In situ infrared thermography of Zn anodes in Zn(HBS)_2_, indicating a uniform surface temperature and a stable SEI. **d** In situ EDH of Zn^2+^ concentration in the same system, revealing homogeneous ion distribution and dendrite suppression [127]. Copyright 2025, Wiley

Building upon these advances, in situ spectroscopic techniques have also been applied to directly probe interfacial processes in hydrogel systems. For example, Novoselov et al. employed in situ FTIR and Raman spectroscopy to investigate the interfacial water structure in D-valine-modified hydrogels. In the unmodified system (ZS-H), the broad ν_O-H_ stretching band (2750–3750 cm^−1^) persisted during Zn plating/stripping, indicating the continuous presence of interfacial free water (Fig. 17a) [80]. In contrast, in the Val-modified hydrogel (Val-H), this band gradually disappeared after the initial plating stage, suggesting progressive dehydration and interfacial reconstruction driven by the accumulation of Val^−^ species under an applied electric field. These anions preferentially adsorb at the inner Helmholtz plane, forming an anion-enriched interfacial layer that alters local coordination environments and redistributes hydrogen-bond configurations. Further analysis using Gaussian fitting and Raman spectroscopy revealed a transition of hydrogen-bond motifs from stronger donor–acceptor configurations (DAA) to weaker ones (DA). This transition reflects a redistribution of hydrogen-bond configurations and a reduction in water activity, suggesting increased structural diversity at the interface. Such changes contribute to suppressing parasitic reactions and stabilizing Zn plating/stripping behavior.

In situ Raman spectroscopy can further extend interfacial characterization from water-structure evolution to ion distribution dynamics. Zhu et al. monitored Zn^2+^ migration within an electron–ion dual transmission channel (EIDC) polymer layer (Fig. 17b) [112]. The progressive increase in the SO_4_^2−^ vibration peak intensity (≈1007 cm^−1^) during Zn deposition, together with Raman mapping, indicates an enrichment of Zn^2+^ near the interface. This observation suggests that coordination sites within the polymer matrix guide Zn^2+^ transport and promote a more uniform ion distribution across the interface.

Beyond spectroscopic techniques, operando visualization methods such as infrared thermography and electron digital holography (EDH) provide complementary information on thermal and concentration fields. In Zn(HBS)_2_ electrolytes, a uniform surface temperature distribution was observed, indicating stable interfacial processes (Fig. 17c) [127]. In contrast, ZnSO_4_ electrolytes exhibited localized overheating and surface irregularities, consistent with inhomogeneous reactions. EDH measurements further revealed that Zn^2+^ concentration evolves uniformly in Zn(HBS)_2_ systems, whereas pronounced depletion zones appear in ZnSO_4_ systems, leading to nonuniform deposition (Fig. 17d).

Collectively, these operando spectroscopic and visualization results demonstrate that changes in coordination environments regulate interfacial chemical processes and ion-migration pathways. Meanwhile, entropy regulation is reflected in the redistribution of hydrogen-bond configurations and interfacial structural states, which contributes to more homogeneous ion transport and stabilized interfacial behavior. These observations establish a direct link between microscopic structural evolution and macroscopic electrochemical performance, providing a reliable experimental basis for understanding the transport and interfacial mechanisms in HPEs.

### In Situ Microscopy and Visualization

Advanced microscopic techniques provide intuitive and spatially resolved visualization of Zn deposition processes, offering direct experimental evidence for understanding the relationship between interfacial structure and electrochemical behavior in HPEs [178]. In this context, coordination environments together with solvation structures, hydrogen-bond configurations and polymer-network features constitute the structural basis of the C-E regulation framework and their evolution can be directly reflected through in situ imaging across multiple spatial and temporal scales.

**Fig. 18:**
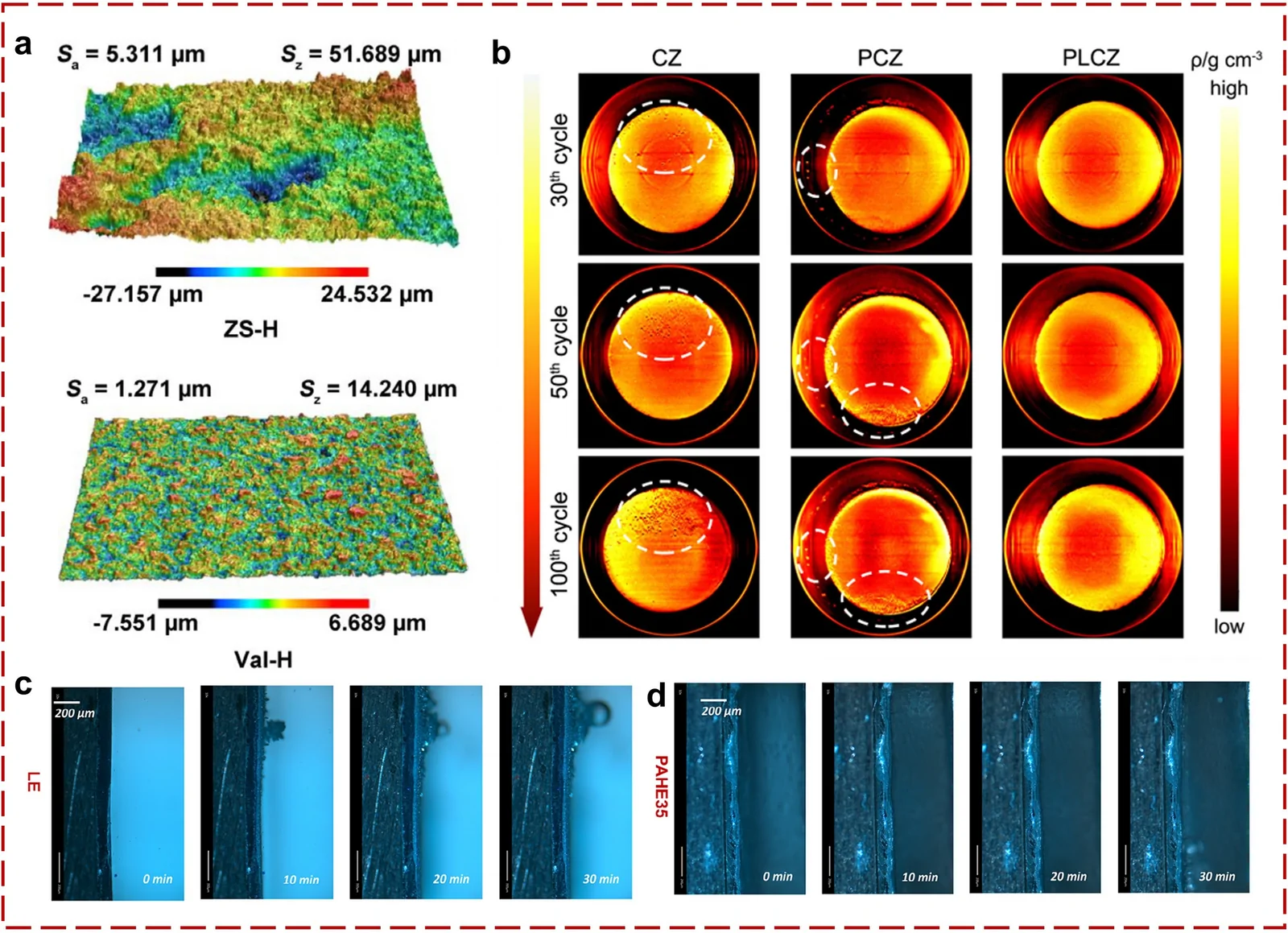
Visualization strategies.** a** 3D LCSM images of Zn anodes in ZS-H and Val-H electrolytes after 1000 cycles, showing effective dendrite suppression in Val-H [80]. Copyright 2025, Wiley. **b** Micro-CT reconstructions of Zn‖Zn cells cycled in CZ, PCZ and PLCZ electrolytes, visualizing dendrites (yellow/orange) and corrosion pits (dark red) [195]. Copyright 2025, Springer Nature. In situ optical microscopy of Zn plating in **c** liquid electrolyte (LE) and **d** PAHE35 hydrogel, where LE shows rapid dendrite growth, while the hydrogel achieves dense, uniform Zn deposition without bubbles [126]. Copyright 2025, Wiley

At the surface level, confocal laser scanning microscopy (CLSM) visualizes the morphological evolution of Zn anodes cycled in unmodified and D-valine-modified hydrogel electrolytes. After 1000 cycles at 0.2 A g^−1^, the pristine hydrogel (ZS-H) exhibits pronounced protrusions and rough surfaces with *S*_*a*_ = 5.311 and *S*_*z*_ = 51.689, whereas the Val-modified hydrogel (Val-H) maintains a smooth and compact morphology with *S*_*a*_ = 1.271 and *S*_*z*_ = 14.240, as shown in Fig. 18a [80]. This contrast indicates that the specific adsorption of Val^−^ anions at the Zn/electrolyte interface regulates local coordination environments and promotes a more uniform ion distribution, thereby suppressing dendritic growth and improving cycling stability in Zn‖I_2_ full cells.

At the three-dimensional electrode scale, microcomputed tomography (Micro-CT) reconstructs the three-dimensional morphology of Zn electrodes in coordination-structured electrolytes. Huang et al. compared CZ, PCZ and PLCZ systems and found that CZ and PCZ exhibit dispersed dendrites and corrosion pits after repeated cycling, whereas PLCZ maintains a dense and flat structure, as presented in Fig. 18b [195]. The reconstructed morphology confirms that uniform Zn deposition is achieved in PLCZ, which can be attributed to the combined effects of coordinated interaction sites and polymer network flexibility, enabling homogeneous ion transport and mechanical confinement within the hydrogel matrix. At the dynamic scale, in situ optical microscopy captures the real-time evolution of Zn plating behavior. In liquid electrolytes, rapid gas evolution leads to the formation of sharp dendrites within a short time, whereas the PAHE35 hydrogel enables dense and layered Zn deposition without observable bubble generation, as shown in Fig. 18c, d [126]. The polymer framework adheres closely to the Zn surface, regulating Zn^2+^ desolvation and modifying interfacial reaction pathways, thereby suppressing hydrogen evolution and corrosion side reactions.

These observations consistently demonstrate that Zn deposition behavior is governed by the coupling between coordination environments and the spatial distribution of structural configurations. As ion distribution becomes more uniform and interfacial reactions are effectively regulated, Zn plating evolves from localized and unstable growth into uniform and compact deposition.

Collectively, in situ imaging further establishes a direct correlation between microscopic structural evolution and macroscopic electrochemical performance by providing real-time visualization of Zn deposition behavior [80, 178]. Coordination regulation determines local interaction environments and interfacial reaction pathways, while entropy regulation is reflected in the redistribution of structural configurations, which promotes homogeneous ion transport and stabilizes interfacial processes.

To provide a balanced perspective, it should be noted that the characterization of hydrogel electrolytes also faces several challenges. The presence of polymer networks and confined water may influence spectroscopic signals, for example through overlapping vibrational features and modified hydrogen-bond signatures, which can complicate spectral interpretation. In addition, the soft and heterogeneous nature of hydrogels can limit spatial resolution and contrast in imaging techniques. These challenges can be addressed by using appropriate control samples, improving spectral resolution and peak deconvolution for overlapping signals, optimizing in situ cell configuration to reduce background interference and dehydration and employing high-resolution or contrast-enhanced imaging methods to better resolve heterogeneous hydrogel structures.

Despite these limitations, the integration of multiscale computational modeling with in situ and operando characterization provides a coherent pathway for linking microscopic structural evolution to macroscopic electrochemical performance in HPEs. These approaches enable the identification of coordination environments, the characterization of solvation and hydrogen-bond structures and the visualization of ion transport and interfacial processes under realistic working conditions. As a result, they offer direct experimental and theoretical insights into how structural features evolve and interact across different length scales. Within this context, the C-E framework is not introduced as an abstract concept but is reflected through experimentally observable structural evolution and transport behavior. Coordination regulation is manifested in the reconstruction of local interaction environments and interfacial reaction pathways, while entropy regulation is reflected in the redistribution and diversification of structural configurations that govern ion transport and interfacial stability. The combination of these effects establishes a consistent structure–property relationship across molecular, mesoscale and macroscopic levels. Therefore, advanced characterization and modeling tools serve not only as analytical techniques but also as essential links that connect material design with electrochemical performance. By enabling a deeper understanding of how coordination regulation and entropy regulation are jointly realized through structural evolution, these approaches provide a practical foundation for guiding the rational design of hydrogel electrolytes toward wide-temperature and long-term stable operation.

## From Laboratory to Commercialization: Opportunities and Challenges of HPEs in AZIBs

Within the C-E regulation framework, HPEs represent an emerging class of quasi-solid-state aqueous electrolytes that integrate high ionic conductivity, structural adaptability and intrinsic safety. In this framework, coordination regulation weakens Zn–H_2_O interactions and lowers ΔH_desolv_, thereby facilitating interfacial charge transfer and suppressing water-induced parasitic reactions. Meanwhile, entropy regulation broadens the distribution of solvation configurations, ion arrangements, transport pathways and polymer-network states, which helps maintain ion-transport continuity and structural adaptability under temperature variation. Through this coupling, the free-energy landscape governing Zn^2+^ transport and interfacial reactions is optimized across temperature variations, enabling stable ion transport at low temperatures while maintaining interfacial stability at elevated temperatures.

Despite these advantages, translating HPE-based AZIBs from laboratory demonstrations to practical technologies requires maintaining effective C-E coupling under realistic constraints, including material cost, scalable processing, long-term reliability and environmental sustainability [196–198]. From a materials-design perspective, this requires balancing coordination effectiveness with structural simplicity. Multicomponent systems can broaden accessible solvation configurations and structural states, thereby contributing to entropy regulation; however, excessive compositional complexity may reduce reproducibility, increase material cost and hinder large-scale fabrication. Therefore, practical electrolyte design should preserve sufficient coordination diversity and structural-state distribution to sustain effective C-E coupling, while avoiding unnecessary formulation complexity [199].

From an engineering perspective, device-level constraints further influence the effectiveness of C-E regulation. HPEs must be compatible with scalable manufacturing processes such as solution casting, coating, lamination and encapsulation, while maintaining controllable thickness, mechanical robustness and stable electrode adhesion. Under practical conditions, including high areal loading, lean-electrolyte operation and prolonged cycling, local Zn^2+^ depletion, water redistribution and interfacial stress accumulation may disrupt coordination environments and entropy-regulated transport pathways [33]. Consequently, maintaining uniform ion flux, stable solvation structures and robust electrode–electrolyte interfaces under non-ideal conditions is essential for preserving C-E coupling and ensuring long-term electrochemical stability.

Sustainability should be regarded as an intrinsic design constraint within the C-E framework rather than a secondary consideration. The environmental impact of hydrogel electrolytes depends not only on material composition but also on synthesis pathways, solvent usage, energy consumption and life-cycle characteristics [200]. Recent studies on biopolymer-based electrolytes highlight that renewable materials do not inherently guarantee sustainability, as multistep processing, high energy input and solvent-intensive synthesis can significantly increase environmental burden. This also indicates that improvements in electrochemical performance do not necessarily correspond to reduced environmental impact, highlighting the need to balance performance optimization with sustainability considerations. Therefore, future C-E guided design should integrate electrochemical performance with sustainability metrics, including carbon footprint, water consumption, material criticality and circularity indicators such as the material circularity indicator and circularity index. In this context, the selection of abundant, renewable or recyclable components, along with low-energy and solvent-efficient processing strategies, will be essential for developing sustainable and scalable electrolyte systems [201].

**Fig. 19:**
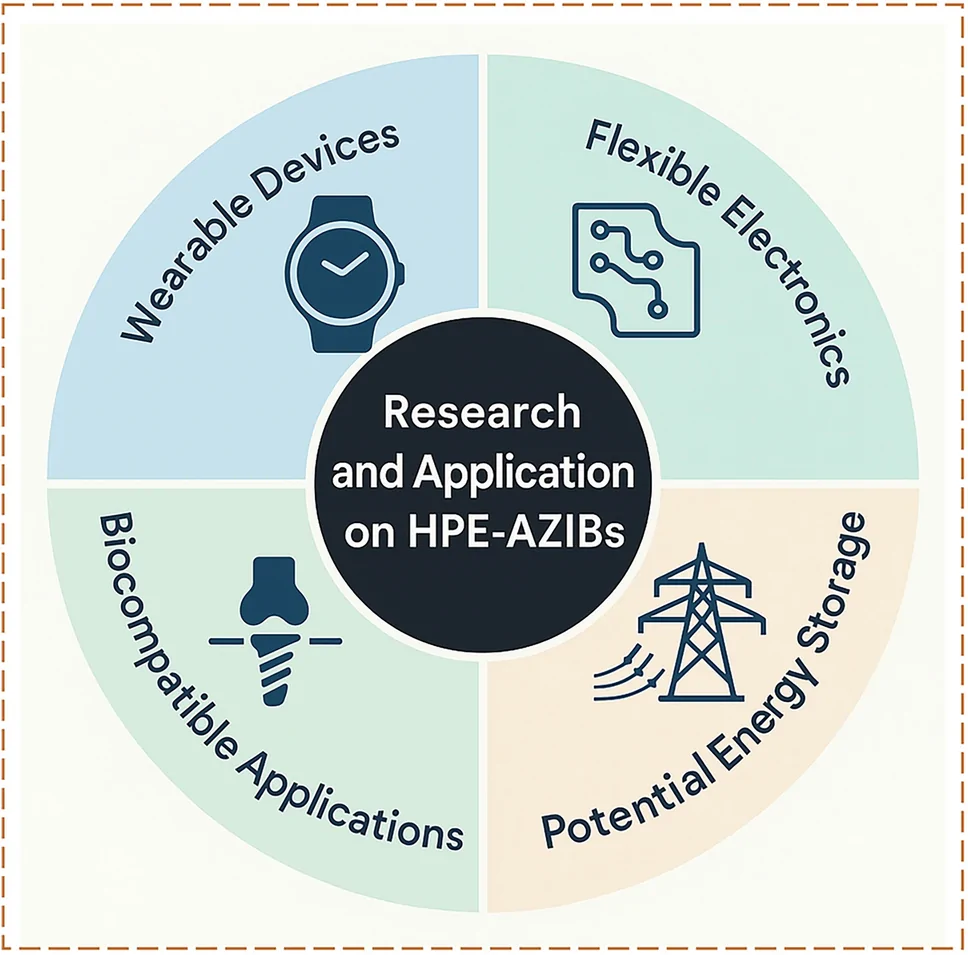
Schematic illustration of the potential application prospects of HPE-AZIBs

The C-E framework also provides guidance for identifying suitable application scenarios for HPE-based AZIBs. In wearable and flexible electronics, entropy-regulated polymer networks enable structural adaptability while maintaining continuous ion-transport pathways under mechanical deformation. In cold-region and distributed energy storage systems, coordination-regulated solvation structures and reduced water activity support stable electrochemical performance under harsh environmental conditions. These application requirements inherently demand stable ion transport and interfacial behavior under dynamically evolving conditions, further emphasizing the importance of maintaining effective C-E coupling across multiple structural scales. Figure 19 illustrates representative application prospects of HPE-AZIBs, including wearable devices, flexible electronics, biocompatible systems and distributed energy storage infrastructures.

Overall, the commercialization of HPE-based AZIBs depends on the ability to maintain effective coordination–entropy coupling under realistic material, engineering and sustainability constraints. Bridging coordination chemistry with scalable material design, environmentally responsible processing and device-level optimization will be essential for translating the C-E framework from a conceptual model into practical electrolyte engineering strategies, thereby enabling its implementation in real-world energy storage systems.

## Conclusions and Outlook

Wide-temperature HPEs have emerged as a promising platform for enabling safe, resilient and environmentally adaptable AZIBs. Throughout this review, recent advances in HPE design have been systematically discussed within the C-E regulation framework, which provides a unified perspective for understanding the coupled roles of Zn^2+^ solvation chemistry, polymer-network structure and electrode–electrolyte interfacial stability. Unlike conventional electrolyte design strategies that focus primarily on optimizing isolated material parameters, the C-E framework emphasizes the cooperative interplay between coordination interactions and entropy-driven structural heterogeneity across multiple length scales.

Within this framework, coordination interactions regulate the thermodynamics and kinetics of Zn^2+^ solvation, desolvation and interfacial charge-transfer processes, thereby shaping the enthalpic landscape governing ion transport and interfacial reactions. Meanwhile, entropy-related structural heterogeneity introduces configurational diversity, adaptive ion-transport pathways and mechanical resilience within polymer networks. The synergistic coupling of these enthalpic and entropic contributions enables uniform Zn^2+^ transport, stabilizes electrode–electrolyte interphases and suppresses parasitic reactions under wide-temperature conditions. Consequently, wide-temperature electrochemical stability can be interpreted as the result of coordinated free-energy redistribution across molecular, mesoscale and interfacial domains.

**Fig. 20:**
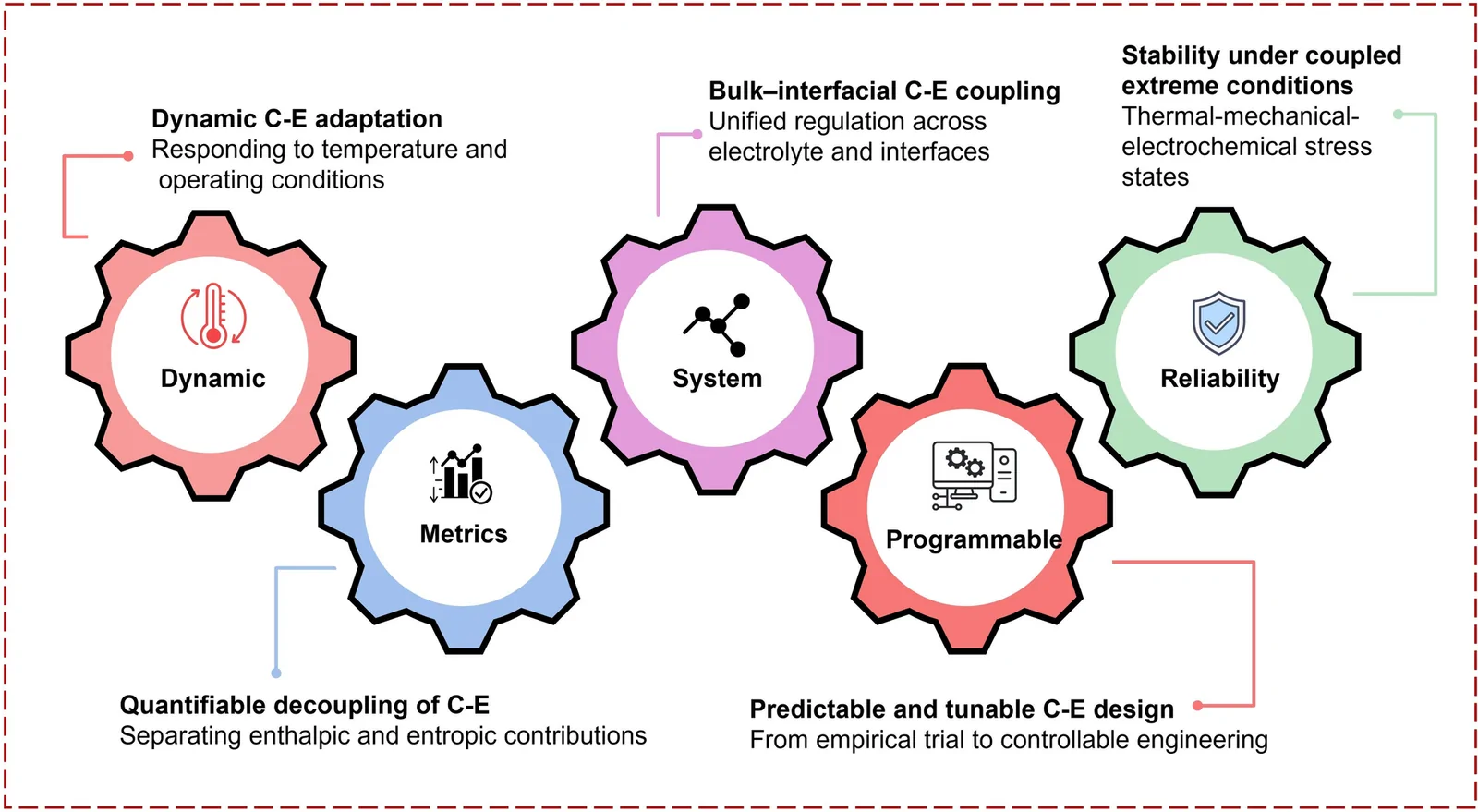
Schematic illustration of strategy-oriented future research directions for wide-temperature HPEs within the C-E regulation framework

More importantly, the C-E framework establishes a rational electrolyte design philosophy for hydrogel systems. In this paradigm, coordination chemistry primarily regulates the enthalpic contributions associated with ion solvation and interfacial reactions, whereas entropy-driven structural heterogeneity governs configurational freedom, ion transport pathways and stress tolerance within polymer networks. The balance between these thermodynamic contributions ultimately determines electrolyte stability, ion transport efficiency and environmental adaptability. This thermodynamic viewpoint also provides a coherent explanation for why many electrolyte systems that perform well under ideal laboratory conditions often fail under extreme or fluctuating operating environments. Despite significant progress in recent years, several key challenges remain for translating the C-E framework into predictive electrolyte design principles and practical battery technologies. As summarized in Fig. 20, future research directions can be broadly categorized into five interconnected aspects.

1. Dynamic C-E adaptation under realistic operating conditions. Most current studies rely on static characterization performed under ideal laboratory conditions. However, practical batteries operate under dynamically evolving thermal, electrochemical and mechanical environments. Understanding how coordination environments and entropy-regulated polymer networks respond to temperature fluctuations, ion-flux variations and mechanical stresses remains a critical challenge. Future progress will require temperature-resolved in situ or operando characterization techniques combined with time-dependent multiscale simulations to reveal the non-equilibrium evolution of coordination states, hydrogen-bond networks and entropy landscapes.
2. Quantifiable C-E descriptors and decoupling of thermodynamic contributions. Although coordination interactions and entropy-driven structural disorder are widely recognized as key determinants of electrolyte performance, their respective contributions remain difficult to quantify experimentally. Establishing measurable descriptors, such as coordination-number distributions, solvation free-energy variations, hydrogen-bond entropy and polymer-network configurational entropy, will be crucial for correlating microscopic structural evolution with macroscopic electrochemical behavior. Integrating advanced characterization methods with free-energy analysis, molecular simulations and data-driven modeling may help establish predictive C-E design rules.
3. System-level C-E coupling across bulk electrolytes and interfaces. Many existing electrolyte design strategies focus on local optimization, for example, modifying Zn^2+^ solvation structures, tuning polymer cross-linking density or introducing interfacial protective layers. However, wide-temperature stability ultimately arises from cooperative coupling among bulk electrolyte structure, interfacial ion transport and mechanical stress relaxation. Future studies should therefore move beyond component-level optimization toward constructing continuous C-E coupling across the electrolyte bulk, electrode interface and device architecture.
4. Programmable electrolyte design guided by C-E principles. The C-E framework provides a rational basis for selecting electrolyte components, including polymer matrices, functional ligands, inorganic fillers and electrolyte additives, according to their roles in shaping coordination environments and entropy landscapes. Through controllable coordination interactions, dynamic cross-linking and entropy-engineered network structures, future electrolyte systems may achieve programmable regulation of ion solvation structures, ion transport pathways and interfacial stability.
5. Reliability of C-E-regulated networks under coupled extreme environments. Beyond temperature variations, practical batteries are exposed to mechanical deformation, pressure fluctuations and long-term cycling. Understanding the reversibility, adaptability and degradation mechanisms of C-E-regulated polymer networks under coupled thermal, mechanical and electrochemical conditions remains essential for practical deployment. Developing accelerated testing protocols that simulate these coupled stress conditions will be critical for evaluating the long-term durability and reliability of wide-temperature HPE-based AZIB systems.

In summary, the C-E regulation framework provides a coherent and transferable paradigm for understanding and designing wide-temperature HPEs for aqueous zinc-ion batteries. By coupling enthalpic coordination control with entropy-driven structural adaptability, the C-E framework transforms wide-temperature electrochemical stability from empirical tolerance into an intrinsic property of electrolyte systems. Continued advances in C-E-guided electrolyte design, multiscale characterization and predictive modeling are expected to accelerate the development of safe, durable and temperature-resilient aqueous energy storage technologies while also providing a conceptual foundation for the design of next-generation soft and hybrid electrochemical systems.

## References

[1] M. Armand, J.M. Tarascon, Building better batteries. Nature **451**(7179), 652–657 (2008). 10.1038/451652a 18256660 10.1038/451652a

[2] A. Manthiram, X. Yu, S. Wang, Lithium battery chemistries enabled by solid-state electrolytes. Nat. Rev. Mater. **2**(4), 16103 (2017). 10.1038/natrevmats.2016.103

[3] D. Zhou, D. Shanmukaraj, A. Tkacheva, M. Armand, G. Wang, Polymer electrolytes for lithium-based batteries: Advances and prospects. Chem **5**(9), 2326–2352 (2019). 10.1016/j.chempr.2019.05.009

[4] J. Liang, Y. Zhu, X. Li, J. Luo, S. Deng et al., A gradient oxy-thiophosphate-coated Ni-rich layered oxide cathode for stable all-solid-state Li-ion batteries. Nat. Commun. **14**(1), 146 (2023). 10.1038/s41467-022-35667-7 36627277 10.1038/s41467-022-35667-7 PMC9832028

[5] Z. Xue, D. He, X. Xie, Poly(ethylene oxide)-based electrolytes for lithium-ion batteries. J. Mater. Chem. A **3**(38), 19218–19253 (2015). 10.1039/c5ta03471j

[6] J. Liang, Y. Sun, Y. Zhao, Q. Sun, J. Luo et al., Engineering the conductive Carbon/PEO interface to stabilize solid polymer electrolytes for all-solid-state high voltage LiCoO_2_ batteries. J. Mater. Chem. A **8**(5), 2769–2776 (2020). 10.1039/c9ta08607b

[7] R. Chen, Q. Li, X. Yu, L. Chen, H. Li, Approaching practically accessible solid-state batteries: Stability issues related to solid electrolytes and interfaces. Chem. Rev. **120**(14), 6820–6877 (2020). 10.1021/acs.chemrev.9b00268 31763824 10.1021/acs.chemrev.9b00268

[8] S. Liu, X. Ji, J. Yue, S. Hou, P. Wang et al., High interfacial-energy interphase promoting safe lithium metal batteries. J. Am. Chem. Soc. **142**(5), 2438–2447 (2020). 10.1021/jacs.9b11750 31927894 10.1021/jacs.9b11750

[9] C. Hu, Y. Shen, M. Shen, X. Liu, H. Chen et al., Superionic conductors via bulk interfacial conduction. J. Am. Chem. Soc. **142**(42), 18035–18041 (2020). 10.1021/jacs.0c07060 32986953 10.1021/jacs.0c07060

[10] Q. Liu, L. Yang, Z. Mei, Q. An, K. Zeng et al., Constructing host–guest recognition electrolytes promotes the Li^+^ kinetics in solid-state batteries. Energy Environ. Sci. **17**(2), 780–790 (2024). 10.1039/d3ee03283c

[11] W. Li, M. Li, S. Wang, P.H. Chien, J. Luo et al., Superionic conducting vacancy-rich β-Li_3_N electrolyte for stable cycling of all-solid-state lithium metal batteries. Nat. Nanotechnol. **20**(2), 265–275 (2025). 10.1038/s41565-024-01813-z 39587350 10.1038/s41565-024-01813-z PMC11835732

[12] M. Wang, Z. Xu, C. He, L. Cai, H. Zheng et al., Fundamentals, advances and perspectives in designing eutectic electrolytes for zinc-ion secondary batteries. ACS Nano **19**(10), 9709–9739 (2025). 10.1021/acsnano.4c18422 40051121 10.1021/acsnano.4c18422

[13] Y. Han, Y. Liu, Y. Zhang, X. He, X. Fu et al., Functionalized quasi-solid-state electrolytes in aqueous Zn-ion batteries for flexible devices: challenges and strategies. Adv. Mater. **37**(1), e2412447 (2025). 10.1002/adma.202412447 39466981 10.1002/adma.202412447

[14] Y. Lv, Y. Xiao, L. Ma, C. Zhi, S. Chen, Recent advances in electrolytes for “beyond aqueous” zinc-ion batteries. Adv. Mater. **34**(4), e2106409 (2022). 10.1002/adma.202106409 34806240 10.1002/adma.202106409

[15] W. Wang, C. Li, S. Liu, J. Zhang, D. Zhang et al., Flexible quasi‐solid‐state aqueous zinc‐ion batteries: design principles, functionalization strategies, and applications. Adv. Energy Mater. **13**(18), 2300250 (2023). 10.1002/aenm.202300250

[16] W. Du, E.H. Ang, Y. Yang, Y. Zhang, M. Ye et al., Challenges in the material and structural design of zinc anode towards high-performance aqueous zinc-ion batteries. Energy Environ. Sci. **13**(10), 3330–3360 (2020). 10.1039/d0ee02079f

[17] Y. Zhang, H. Zhuo, P. Lei, D. Tang, Q. Hu et al., Fukui function-engineered gel electrolytes: thermodynamic/kinetic-synergistic regulation for long-cycling zinc metal batteries. Adv. Mater. **37**(38), e2508722 (2025). 10.1002/adma.202508722 40557535 10.1002/adma.202508722

[18] X. Yu, Z. Li, X. Wu, H. Zhang, Q. Zhao et al., Ten concerns of Zn metal anode for rechargeable aqueous zinc batteries. Joule **7**(6), 1145–1175 (2023). 10.1016/j.joule.2023.05.004

[19] R. Zhao, Z. Feng, R. Kuang, Z. Li, K. Lu et al., UV‐Polymerized zincophilic ion‐enhanced interfacial layer with high ion transference number for ultrastable Zn metal anodes. Carbon Neutralization **4**(2), e194 (2025). 10.1002/cnl2.194

[20] T. Xiong, Y. Guo, X. Wang, Design and structure of electrolytes for all‐weather aqueous zinc batteries. Adv. Funct. Mater. **35**(16), 2421240 (2024). 10.1002/adfm.202421240

[21] Y. Li, Z. Yu, J. Huang, Y. Wang, Y. Xia, Constructing solid electrolyte interphase for aqueous zinc batteries. Angew. Chem. Int. Ed. **62**(47), e202309957 (2023). 10.1002/anie.202309957 10.1002/anie.202309957 37596841

[22] P. Ruan, S. Liang, B. Lu, H.J. Fan, J. Zhou, Design strategies for high-energy-density aqueous zinc batteries. Angew. Chem. Int. Ed. **61**(17), e202200598 (2022). 10.1002/anie.202200598 10.1002/anie.202200598 35104009

[23] X. Xu, Y. Xu, J. Zhang, Y. Zhong, Z. Li et al., Quasi-solid electrolyte interphase boosting charge and mass transfer for dendrite-free zinc battery. Nano-Micro Lett. **15**(1), 56 (2023). 10.1007/s40820-023-01031-7 10.1007/s40820-023-01031-7 PMC9975136 36853520

[24] L. Tang, H. Peng, J. Kang, H. Chen, M. Zhang et al., Zn-based batteries for sustainable energy storage: strategies and mechanisms. Chem. Soc. Rev. **53**(10), 4877–4925 (2024). 10.1039/d3cs00295k 38595056 10.1039/d3cs00295k

[25] Y. Hu, Z. Wang, Y. Li, P. Liu, X. Liu et al., Sulfonated hydrogel electrolyte enables dendrite-free zinc-ion batteries. Chem. Eng. J. **479**, 147762 (2024). 10.1016/j.cej.2023.147762

[26] L. Jiang, S. Han, Y.-C. Hu, Y. Yang, Y. Lu et al., Rational design of anti-freezing electrolytes for extremely low-temperature aqueous batteries. Nat. Energy **9**(7), 839–848 (2024). 10.1038/s41560-024-01527-5

[27] Q. Meng, T. Yan, Y. Wang, X. Lu, H. Zhou et al., Critical design strategy of electrolyte engineering toward aqueous zinc-ion battery. Chem. Eng. J. **497**, 154541 (2024). 10.1016/j.cej.2024.154541

[28] T. Xue, J. Guan, Y. Mu, M. Han, C. Yang et al., Quasi-solid-state electrolytes: bridging the gap between solid and liquid electrolytes for zinc-ion batteries. Chem. Eng. J. **514**, 162994 (2025). 10.1016/j.cej.2025.162994

[29] H. Ge, X. Xie, X. Xie, B. Zhang, S. Li et al., Critical challenges and solutions: quasi-solid-state electrolytes for zinc-based batteries. Energy Environ. Sci. **17**(10), 3270–3306 (2024). 10.1039/d4ee00357h

[30] H. Peng, D. Wang, F. Zhang, L. Yang, X. Jiang et al., Improvements and challenges of hydrogel polymer electrolytes for advanced zinc anodes in aqueous zinc-ion batteries. ACS Nano **18**(33), 21779–21803 (2024). 10.1021/acsnano.4c06502 39132720 10.1021/acsnano.4c06502

[31] X. Zhou, Y. Zhou, L. Yu, L. Qi, K.S. Oh et al., Gel polymer electrolytes for rechargeable batteries toward wide-temperature applications. Chem. Soc. Rev. **53**(10), 5291–5337 (2024). 10.1039/d3cs00551h 38634467 10.1039/d3cs00551h

[32] Y. Liu, Y. Sun, K. Zhang, L. Xia, L. Yuan et al., Synergistic ionic and hydrogen-bond locking in hydrogel electrolytes for all-climate high-performance flexible supercapacitors. Energy Storage Mater. **89**, 105201 (2026). 10.1016/j.ensm.2026.105201

[33] J. Li, A. Azizi, S. Zhou, S. Liu, C. Han et al., Hydrogel polymer electrolytes toward better zinc-ion batteries: a comprehensive review. eScience **5**(2), 100294 (2025). 10.1016/j.esci.2024.100294

[34] W. Lv, J. Liu, Z. Shen, X. Li, C. Xu, Novel approaches to aqueous zinc-ion batteries: challenges, strategies, and prospects. eScience **5**(6), 100410 (2025). 10.1016/j.esci.2025.100410

[35] X. Jia, C. Liu, Z.G. Neale, J. Yang, G. Cao, Active materials for aqueous zinc ion batteries: synthesis, crystal structure, morphology, and electrochemistry. Chem. Rev. **120**(15), 7795–7866 (2020). 10.1021/acs.chemrev.9b00628 32786670 10.1021/acs.chemrev.9b00628

[36] J. Luan, H. Yuan, J. Liu, C. Zhong, Recent advances on charge storage mechanisms and optimization strategies of Mn-based cathode in zinc–manganese oxides batteries. Energy Storage Mater. **66**, 103206 (2024). 10.1016/j.ensm.2024.103206

[37] Y. Liu, S.-G. Han, X. Li, Y. Luo, Y. Wu et al., Manganese dioxide cathode materials for aqueous zinc ion battery: Development, challenges and strategies. Energy Chem **7**(3), 100152 (2025). 10.1016/j.enchem.2025.100152

[38] X. Bai, M. Sun, J. Yang, B. Deng, K. Yang et al., Eutectic-electrolyte-enabled zinc metal batteries towards wide temperature and voltage windows. Energy Environ. Sci. **17**(19), 7330–7341 (2024). 10.1039/d4ee02816c

[39] X. Wu, Q. Yan, H. Wang, D. Wu, H. Zhou et al., Heterostructured catalytic materials as advanced electrocatalysts: classification, synthesis, characterization, and application. Adv. Funct. Mater. **34**(42), 2404535 (2024). 10.1002/adfm.202404535

[40] X. Wu, Y. Chen, B. Tang, Q. Yan, D. Wu et al., CeO_x_-integrated dual site enhanced urea electrosynthesis from nitrate and carbon dioxide. Nat. Commun. **16**(1), 8785 (2025). 10.1038/s41467-025-63839-8 41038827 10.1038/s41467-025-63839-8 PMC12491563

[41] D. Wu, H. Wang, Y. Nie, H. Wan, S. Liu et al., When covalent organic frameworks meet metals: from opportunities toward applications. Prog. Mater. Sci. **155**, 101538 (2026). 10.1016/j.pmatsci.2025.101538

[42] J. Tang, Z. Dai, C. Yang, R. Chanajaree, M. Okhawilai et al., Breaking the water activity barrier: hydrated eutectic electrolytes for long-cycling and wide-temperature zinc-ion batteries. Adv. Funct. Mater. **36**(7), e15911 (2025). 10.1002/adfm.202515911

[43] Z. Zhao, P. Li, Y. Wu, Z. Chai, H. Zhang et al., Fluorinated interphase enabled by lithium salt‐driven electrical double‐layer modulation for advanced zinc metal batteries. Adv. Funct. Mater. **35**(46), 2506270 (2025). 10.1002/adfm.202506270

[44] M. Su, H. Dou, J. Yan, S. Liu, M. Xu et al., Multisite cooperative regulation of solvation and interface via dynamic additive engineering for highly reversible zinc batteries. Angew. Chem. Int. Ed. **64**(38), e202511685 (2025). 10.1002/anie.202511685 10.1002/anie.202511685 40745980

[45] M. Chen, W. Fu, C. Hou, Y. Zhu, F. Meng, Tailored electronegative-driven MOF channels with bimetallic coordination for synergistic Zn^2+^ transport enabling dendrite-free Zn anodes. Energy Storage Mater. **80**, 104380 (2025). 10.1016/j.ensm.2025.104380

[46] Y. Mu, Z. Li, B.K. Wu, H. Huang, F. Wu et al., 3D hierarchical graphene matrices enable stable Zn anodes for aqueous Zn batteries. Nat. Commun. **14**(1), 4205 (2023). 10.1038/s41467-023-39947-8 37452017 10.1038/s41467-023-39947-8 PMC10349079

[47] X. Zhao, Z. Gong, G. Wang, F. Zhang, N. Chen et al., Preferential texture of surface coating on Zn anodes for advanced aqueous batteries: small change but big gain. Angew. Chem. Int. Ed. **64**(44), e202509952 (2025). 10.1002/anie.202509952 10.1002/anie.202509952 40898738

[48] Z. Peng, L. Tang, S. Li, L. Tan, Y. Chen, Strong replaces weak: hydrogen bond-anchored electrolyte enabling ultra-stable and wide-temperature aqueous zinc-ion capacitors. Angew. Chem. Int. Ed. **64**(6), e202418242 (2025). 10.1002/anie.202418242 10.1002/anie.202418242 39528859

[49] G. Ji, M. Sun, M. Li, J. Zheng, Sulfonate‐modified covalent organic framework integrated hydrogel electrolyte: enhancing AZIBs performances by tailoring microstructures and functional groups. Adv. Funct. Mater. **35**(34), 2500110 (2025). 10.1002/adfm.202500110

[50] R. Qi, W. Tang, Y. Shi, K. Teng, Y. Deng et al., Gel polymer electrolyte toward large‐scale application of aqueous zinc batteries. Adv. Funct. Mater. **33**(47), 2306052 (2023). 10.1002/adfm.202306052

[51] Z.J. Chen, T.Y. Shen, X. Xiao, X.C. He, Y.L. Luo et al., An ultrahigh-modulus hydrogel electrolyte for dendrite-free zinc ion batteries. Adv. Mater. **36**(52), e2413268 (2024). 10.1002/adma.202413268 39543445 10.1002/adma.202413268

[52] Y. Yan, S. Duan, B. Liu, S. Wu, Y. Alsaid et al., Tough hydrogel electrolytes for anti-freezing zinc-ion batteries. Adv. Mater. **35**(18), e2211673 (2023). 10.1002/adma.202211673 36932878 10.1002/adma.202211673

[53] Y. Lin, J. Huang, S. Wang, L. Qi, W. Chen et al., Stretchable, adhesive, anti-freezing hydrogel electrolytes with dual-functional water regulation enabled by amide group-salt-water interactions for all-climate zinc-ion batteries. Adv. Mater. **37**(41), e09975 (2025). 10.1002/adma.202509975 40785419 10.1002/adma.202509975

[54] Y. Zheng, D. Wu, T. Wang, Q. Liu, D. Jia, Advanced eutectogel electrolyte for high-performance and wide-temperature flexible zinc-air batteries. Angew. Chem. Int. Ed. **64**(6), e202418223 (2025). 10.1002/anie.202418223 10.1002/anie.202418223 39400426

[55] H. Wang, Z. Hou, Y. Wang, H. Long, D. Zhang et al., 3D hierarchical porous hydrogel polymer electrolytes for flexible quasi-solid-state supercapacitors. Chem. Eng. J. **510**, 161766 (2025). 10.1016/j.cej.2025.161766

[56] J. Wang, X. Chen, S. Jin, Y. Zhang, Z. Yang et al., 60–50 ℃ ultrawide-temperature flexible zinc–air batteries enabled by a ternary polar hydrogel electrolyte. Energy Environ. Sci. **18**(21), 9502–9511 (2025). 10.1039/d5ee03421c

[57] J. Zhai, W. Zhao, L. Wang, J. Shuai, R. Chen et al., Ultrathin cellulosic gel electrolytes with a gradient hydropenic interface for stable, high-energy and flexible zinc batteries. Energy Environ. Sci. **18**(9), 4241–4250 (2025). 10.1039/d5ee00158g

[58] H. Zhang, L. Wan, Z. You, J. Liang, D. Lei et al., Chain-chain synergistic hydrogel electrolytes regulate zinc ions desolvation for stabilized anodes and high operating voltage in flexible zinc ions hybrid capacitors. Angew. Chem. Int. Ed. **64**(32), e202507403 (2025). 10.1002/anie.202507403 10.1002/anie.202507403 40437769

[59] S. Yang, Q. Wu, Y. Li, F. Luo, J. Zhang et al., A bio-inspired multifunctional hydrogel network with toughly interfacial chemistry for dendrite-free flexible zinc ion battery. Angew. Chem. Int. Ed. **63**(44), e202409160 (2024). 10.1002/anie.202409160 10.1002/anie.202409160 39113640

[60] J. Chen, F. Bu, Q. Cao, W. Zhao, Y. Gao et al., Electrostatic potential-dominated weak solvation chemistry for synergistic optimization of V_2_O_5_ cathode and Zn anode. Angew. Chem. Int. Ed. **64**(38), e202510638 (2025). 10.1002/anie.202510638 10.1002/anie.202510638 40761022

[61] Y. Liu, M. Qiu, Y. Liang, J. Zhang, J. Chen et al., Balancing tetrahedral and cation entropies for long-lifespan low-temperature Zn-ion batteries. Angew. Chem. Int. Ed. **64**(36), e202506010 (2025). 10.1002/anie.202506010 10.1002/anie.202506010 40641133

[62] M. Sun, G. Ji, M. Li, J. Zheng, A robust hydrogel electrolyte with ultrahigh ion transference number derived from zincophilic “chain-gear” network structure for dendrite-free aqueous zinc ion battery. Adv. Funct. Mater. **34**(37), 2402004 (2024). 10.1002/adfm.202402004

[63] T. Liu, X. Dong, J. Zhang, H. Chen, R. Cao et al., Concentration-function coupled electrolytes harmonize thermodynamics and kinetics for stable zinc metal batteries. Chem. Sci. **16**(37), 17426–17435 (2025). 10.1039/d5sc05421d 40904485 10.1039/d5sc05421d PMC12403028

[64] Y. Chen, S. Zhou, J. Li, X. Zhang, C. Zhou et al., Tuning Zn^2+^ deposition kinetics towards deep-reversible zinc metal batteries with all-climate adaptability. Angew. Chem. Int. Ed. **64**(18), e202423252 (2025). 10.1002/anie.202423252 10.1002/anie.202423252 39979219

[65] Y. Zhao, Y. Wang, J. Li, J. Xiong, Q. Li et al., Thermodynamic and kinetic insights for manipulating aqueous Zn battery chemistry: towards future grid-scale renewable energy storage systems. eScience **5**(4), 100331 (2025). 10.1016/j.esci.2024.100331

[66] Z. Chen, Y. Xu, R. Jiang, H. Zhu, Q. Zhang et al., Chelation and interfacial engineering for long‐term cycling and self‐healing aqueous zinc batteries. Adv. Funct. Mater. **36**(11), e12356 (2025). 10.1002/adfm.202512356

[67] Y. Yang, Q. He, C. Hu, X. Xie, S. Liang et al., Electron-initiated self-growth in situ hydrogel electrolyte with gradient protection interface enables stable zinc metal batteries. ACS Nano **19**(23), 21717–21728 (2025). 10.1021/acsnano.5c04942 40465427 10.1021/acsnano.5c04942

[68] T. Zhang, Y. Tang, S. Guo, X. Cao, A. Pan et al., Fundamentals and perspectives in developing zinc-ion battery electrolytes: a comprehensive review. Energy Environ. Sci. **13**(12), 4625–4665 (2020). 10.1039/d0ee02620d

[69] H. Yan, S. Li, J. Zhong, B. Li, An electrochemical perspective of aqueous zinc metal anode. Nano-Micro Lett. **16**(1), 15 (2023). 10.1007/s40820-023-01227-x 10.1007/s40820-023-01227-x PMC10656387 37975948

[70] Y. Yuan, J. Chen, T. Qian, B. Zhang, K. Ye et al., Hydrogen atom capture toward dense solid electrolyte interface for long-cycling aqueous zinc-ion batteries. Angew. Chem. Int. Ed. **64**(48), e202513722 (2025). 10.1002/anie.202513722 10.1002/anie.202513722 41013952

[71] J. Lin, Y. Wang, M. Chen, J. Lu, H. Mi et al., Regulating the Gibbs free energy to design aqueous battery‐compatible robust host. Adv. Energy Mater. **14**(31), 2401275 (2024). 10.1002/aenm.202401275

[72] J.B. Goodenough, K.S. Park, The Li-ion rechargeable battery: a perspective. J. Am. Chem. Soc. **135**(4), 1167–1176 (2013). 10.1021/ja3091438 23294028 10.1021/ja3091438

[73] W. Zhang, T. Xiong, X. Qiu, Electrolyte engineering for future aqueous Zn-ion batteries. Cell Rep. Phys. Sci. **6**(10), 102853 (2025). 10.1016/j.xcrp.2025.102853

[74] Y. Zhou, Y. Pan, Y. Yang, T.F. Altamimi, Y. Zhong et al., Electrically insulating rigid multi-channel electrolyte container for customizable electron transfer in Zn-halogen batteries. Nano-Micro Lett. **18**(1), 168 (2026). 10.1007/s40820-025-02007-5 10.1007/s40820-025-02007-5 PMC12765777 41486399

[75] J. Wei, P. Zhang, J. Sun, Y. Liu, F. Li et al., Advanced electrolytes for high-performance aqueous zinc-ion batteries. Chem. Soc. Rev. **53**(20), 10335–10369 (2024). 10.1039/d4cs00584h 39253782 10.1039/d4cs00584h

[76] R. Jia, C. Wei, B. Ma, L. Li, C. Yang et al., Biopolymer-based gel electrolytes for advanced zinc ion batteries: progress and perspectives. Adv. Funct. Mater. **35**(12), 2417498 (2024). 10.1002/adfm.202417498

[77] Z. Liu, F. Feng, W. Feng, G. Wang, B. Qi et al., Eutectic electrolytes: a new platform for high-safety batteries. Energy Environ. Sci. **18**(8), 3568–3613 (2025). 10.1039/d4ee05298f

[78] F. Ai, Y.-C. Lu, Coordination chemistry in advanced redox-active electrolyte designs. Nat. Rev. Mater. **10**(12), 929–946 (2025). 10.1038/s41578-025-00833-y

[79] D. Lin, Y. Lin, R. Pan, J. Li, A. Zhu et al., Water-restrained hydrogel electrolytes with repulsion-driven cationic express pathways for durable zinc-ion batteries. Nano-Micro Lett. **17**(1), 193 (2025). 10.1007/s40820-025-01704-5 10.1007/s40820-025-01704-5 PMC11920515 40102362

[80] J. Lin, C. Ji, G. Guo, Y. Luo, P. Huang et al., Interfacial h-bond network/concentration fields/electric fields regulation achieved by d-valine anions realizes the highly efficient aqueous zinc ion batteries. Angew. Chem. Int. Ed. **64**(24), e202501721 (2025). 10.1002/anie.202501721 10.1002/anie.202501721 40202425

[81] F. Luo, S. Yang, Q. Wu, Y. Li, J. Zhang et al., Hydrogel electrolytes with an electron/ion dual regulation mechanism for highly reversible flexible zinc batteries. Energy Environ. Sci. **17**(22), 8570–8581 (2024). 10.1039/d4ee03067b

[82] W. Wang, C. Li, W. Zhuang, B. Wu, P. Wang et al., Rational constructions of high-performance flexible aqueous gel-state zinc ionic batteries. Adv. Funct. Mater. **35**(45), 2423311 (2025). 10.1002/adfm.202423311

[83] Z. Shen, Y. Liu, Z. Li, Z. Tang, J. Pu et al., Highly-entangled hydrogel electrolyte for fast charging/discharging properties in aqueous zinc ion batteries. Adv. Funct. Mater. **35**(21), 2406620 (2025). 10.1002/adfm.202570124

[84] Y. Zhu, H. Zhang, M. Tao, Y. Yan, X. Gan et al., A universal janus biopolymer separator enabled dual-interfacial regulation toward dendrite-free and shuttle-free zinc-iodine batteries. Adv. Mater. **38**(24), e72959 (2026). 10.1002/adma.72959 41906522 10.1002/adma.72959

[85] H. Liu, Q. Zhou, Q. Xia, Y. Lei, X.L. Huang et al., Interface challenges and optimization strategies for aqueous zinc-ion batteries. J. Energy Chem. **77**, 642–659 (2023). 10.1016/j.jechem.2022.11.028

[86] Z. Shen, Z. Zhai, Y. Liu, X. Bao, Y. Zhu et al., Hydrogel electrolytes-based rechargeable zinc-ion batteries under harsh conditions. Nano-Micro Lett. **17**(1), 227 (2025). 10.1007/s40820-025-01727-y 10.1007/s40820-025-01727-y PMC12015001 40261597

[87] H. Zhang, X. Gan, Y. Gao, H. Wu, Z. Song et al., Carboxylic acid-functionalized cellulose hydrogel electrolyte for dual-interface stabilization in aqueous zinc-organic batteries. Adv. Mater. **37**(1), e2411997 (2025). 10.1002/adma.202411997 39520341 10.1002/adma.202411997

[88] S. Zhang, H. Ao, J. Dong, D. Wang, C. Wang et al., Dipole moment dictates the preferential immobilization in gel electrolytes for Ah-level aqueous zinc-metal batteries. Angew. Chem. Int. Ed. **64**(2), e202414702 (2025). 10.1002/anie.202414702 10.1002/anie.202414702 39320088

[89] B. Sambandam, V. Mathew, S. Kim, S. Lee, S. Kim et al., An analysis of the electrochemical mechanism of manganese oxides in aqueous zinc batteries. Chem **8**(4), 924–946 (2022). 10.1016/j.chempr.2022.03.019

[90] L.-L. Zhao, Y.-H. Zhao, Y.-M. Wu, P.-F. Wang, Z.-L. Liu et al., Everything in aqueous zinc-ion batteries may be Prussian blue analogues: from cathode materials to electrolyte additives applications. Energy Storage Mater. **78**, 104299 (2025). 10.1016/j.ensm.2025.104299

[91] W. Chen, T. Chen, J. Fu, Pivotal role of organic materials in aqueous zinc‐based batteries: regulating cathode, anode, electrolyte, and separator. Adv. Funct. Mater. **34**(3), 2308015 (2023). 10.1002/adfm.202308015

[92] D. Li, Y. Guo, C. Zhang, X. Chen, W. Zhang et al., Unveiling organic electrode materials in aqueous zinc-ion batteries: from structural design to electrochemical performance. Nano-Micro Lett. **16**(1), 194 (2024). 10.1007/s40820-024-01404-6 10.1007/s40820-024-01404-6 PMC11093963 38743294

[93] Z. Li, J. Tan, Y. Wang, C. Gao, Y. Wang et al., Building better aqueous Zn-organic batteries. Energy Environ. Sci. **16**(6), 2398–2431 (2023). 10.1039/d3ee00211j

[94] C. Yan, L. Zhu, P. Li, J. Tang, H. He et al., Multi-level Zn^2+^-buffering interphase enabled by hierarchical nanostructure engineering of gel polymers for highly reversible zinc metal anode. Adv. Mater. **38**(4), e15316 (2026). 10.1002/adma.202515316 41078027 10.1002/adma.202515316

[95] Z. Song, S. Yuan, X. Zhang, X. Zhao, Q. Zou, High‐performance MnO_2_ hydrogel composite electrodes constructed via in situ Hofmeister effect for zinc–ion batteries. Adv. Energy Mater. **15**(37), e02994 (2025). 10.1002/aenm.202502994

[96] L. Yao, L. Jiang, Y. Wang, X. Chi, Y. Liu, A 1000 wh kg^-1^ cathode facilitated by in situ mineralized electrolyte with electron potential well for high-energy aqueous zinc batteries. Adv. Mater. **37**(41), e05342 (2025). 10.1002/adma.202505342 40678953 10.1002/adma.202505342

[97] L. Zhang, Y. Zhang, X. Wang, X. Wang, Q. Wang et al., A six‐electron‐transfer organic cathode for aqueous zinc batteries. Adv. Funct. Mater. **36**(13), e13189 (2025). 10.1002/adfm.202513189

[98] C. Ding, Y. Wang, C. Li, J. Wang, Q. Zhang et al., Constructing ultra-stable, high-energy, and flexible aqueous zinc-ion batteries using environment-friendly organic cathodes. Chem. Sci. **15**(13), 4952–4959 (2024). 10.1039/d4sc00491d 38550696 10.1039/d4sc00491d PMC10967254

[99] D. Luo, L. Yang, Z. Geng, H. Liu, X. Zhong et al., Underlying principles and practical design strategies of hydrogel electrolytes for long‐term stable zinc batteries. Adv. Energy Mater. **16**(1), e04151 (2025). 10.1002/aenm.202504151

[100] X. Cai, X. Li, J. Liang, J. Qiu, W. Lin et al., Stabilizing the anode and cathode interface synchronously via electrolyte-triggered hydrogel interphase for zinc metal batteries. Nano-Micro Lett. **18**(1), 206 (2026). 10.1007/s40820-025-02051-1 10.1007/s40820-025-02051-1 PMC12799898 41528632

[101] C. Zhang, X. Xu, Z. Chen, Q. Li, Y. Zhong et al., Localized water confinement via micellar electrolyte for aqueous zinc-vanadium batteries. Adv. Mater. **38**(4), e10792 (2026). 10.1002/adma.202510792 41099156 10.1002/adma.202510792

[102] H. Zhang, X. Gan, Z. Song, J. Zhou, Amphoteric cellulose-based double-network hydrogel electrolyte toward ultra-stable Zn anode. Angew. Chem. Int. Ed. **62**(13), e202217833 (2023). 10.1002/anie.202217833 10.1002/anie.202217833 36720709

[103] J. Yang, C. Weng, P. Sun, Y. Yin, M. Xu et al., Comprehensive regulation strategies for gel electrolytes in aqueous zinc-ion batteries. Coord. Chem. Rev. **530**, 216475 (2025). 10.1016/j.ccr.2025.216475

[104] Q. He, Y. Zhong, J. Li, S. Chai, Y. Yang et al., Constructing kosmotropic salt‐compatible PVA hydrogels for stable zinc anodes via strong hydrogen bonds preshielding effect. Adv. Energy Mater. **14**(23), 2400170 (2024). 10.1002/aenm.202400170

[105] H. Tian, M. Yao, Y. Guo, Z. Wang, D. Xu et al., Hydrogel electrolyte with regulated water activity and hydrogen bond network for ultra‐stable zinc electrode. Adv. Energy Mater. **15**(9), 2403683 (2024). 10.1002/aenm.202403683

[106] L. Wang, H. Wang, J. Cao, J. Yan, C. Dai et al., Restructuring hydrogen bond networks in polyacrylamide hydrogels via trehalose additives for wide-temperature-range Zn-ion batteries. ACS Nano **19**(31), 28397–28409 (2025). 10.1021/acsnano.5c06805 40791027 10.1021/acsnano.5c06805

[107] S. Cui, W. Miao, X. Wang, K. Sun, H. Peng et al., Multifunctional zincophilic hydrogel electrolyte with abundant hydrogen bonds for zinc-ion capacitors and supercapacitors. ACS Nano **18**(19), 12355–12366 (2024). 10.1021/acsnano.4c01304 38683957 10.1021/acsnano.4c01304

[108] D. Lu, Y. Hao, Z. Wang, J. He, X. Huang et al., Zn-PAA-C hydrogel for integrated energy storage and self-diagnostic health monitoring in wearable biomedical devices. Energy Storage Mater. **80**, 104407 (2025). 10.1016/j.ensm.2025.104407

[109] H. Yuan, J. Luan, Q. Zhang, J. Liu, N. Zhao et al., High hydrophilic/zincophilic interpenetrating double-network hydrogel electrolyte constructing stable organic-inorganic anode interface toward nickel–zinc batteries. Nano Energy **134**, 110595 (2025). 10.1016/j.nanoen.2024.110595

[110] M. Sun, G. Ji, J. Zheng, A hydrogel electrolyte with ultrahigh ionic conductivity and transference number benefit from Zn^2+^ “highways” for dendrite-free Zn-MnO_2_ battery. Chem. Eng. J. **463**, 142535 (2023). 10.1016/j.cej.2023.142535

[111] P. Lin, J. Cong, J. Li, M. Zhang, P. Lai et al., Achieving ultra-long lifespan Zn metal anodes by manipulating desolvation effect and Zn deposition orientation in a multiple cross-linked hydrogel electrolyte. Energy Storage Mater. **49**, 172–180 (2022). 10.1016/j.ensm.2022.04.010

[112] D. Wang, D. Zhao, L. Chang, Y. Zhang, W. Wang et al., Interface engineering of electron-ion dual transmission channels for ultra-long lifespan quasi-solid zinc-ion batteries. Energy Storage Mater. **74**, 103903 (2025). 10.1016/j.ensm.2024.103903

[113] M. Xu, F. Liu, L. Chen, Y. Lei, Z. Liu et al., Zwitterionic poly(ionic liquid) hydrogel electrolytes with high-speed ion conduction channels for dendrite-free, long-enduring zinc-ion batteries and flexible electronics. Energy Storage Mater. **80**, 104373 (2025). 10.1016/j.ensm.2025.104373

[114] J. Huang, S. Wang, L. Yu, E. Lizundia, X. Zeng et al., Practical, sustainable, wide-temperature-adaptable zinc-metal batteries enabled by electrogelated recyclable biomacromolecular hydrogel electrolytes. Natl. Sci. Rev. **12**(9), nwaf308 (2025). 10.1093/nsr/nwaf308 40933453 10.1093/nsr/nwaf308 PMC12418938

[115] Z. Zhang, X. Wang, J. Ke, W. Du, Z. Lv et al., Approaching 100% comprehensive utilization rate of ultra‐stable Zn metal anodes by constructing chitosan‐based homologous gel/solid synergistic interface. Adv. Funct. Mater. **34**(17), 2313150 (2024). 10.1002/adfm.202313150

[116] J. Kumankuma-Sarpong, C. Chang, J. Hao, T. Li, X. Deng et al., Entanglement added to cross-linked chains enables tough gelatin-based hydrogel for Zn metal batteries. Adv. Mater. **36**(30), e2403214 (2024). 10.1002/adma.202403214 38748854 10.1002/adma.202403214

[117] J. Peng, W. Song, W. Zhang, X. Li, Q. Ma et al., Bone-inspired sustainable hydrogel electrolytes for Zn metal batteries. Angew. Chem. Int. Ed. **64**(34), e202506449 (2025). 10.1002/anie.202506449 10.1002/anie.202506449 40438902

[118] C. Tian, J. Wang, R. Sun, T. Ali, H. Wang et al., Improved interfacial ion migration and deposition through the chain-liquid synergistic effect by a carboxylated hydrogel electrolyte for stable zinc metal anodes. Angew. Chem. Int. Ed. **62**(42), e202310970 (2023). 10.1002/anie.202310970 10.1002/anie.202310970 37644643

[119] Q. Zheng, L. Liu, Z. Hu, Z. Tang, H. Lu et al., Altering the Zn^2+^ migration mechanism enables the composite hydrogel electrolytes with high Zn^2+^ conduction and superior anti‐dehydration. Adv. Funct. Mater. **35**(35), 2504782 (2025). 10.1002/adfm.202504782

[120] Y. Liu, J. Chen, M. Qiu, P. Sun, W. Mai, Designing better aqueous electrolyte for wide‐temperature‐adaptive Zn‐based batteries: from the perspective of water structure order. Adv. Funct. Mater. **35**(49), e04127 (2025). 10.1002/adfm.202504127

[121] Y. Dong, X. Chai, J. Li, L. Xue, F. Kong et al., High-entropy electrolytes downsizing solvated clusters and enabling parallel ion transport for low-temperature aqueous zinc batteries. J. Am. Chem. Soc. **147**(39), 35544–35554 (2025). 10.1021/jacs.5c10307 40974324 10.1021/jacs.5c10307

[122] G. Li, B. Guo, M. Shi, L. Wen, J. Luo et al., Sulfonate-mediated cation-switching in hydrogel electrolytes to unlock fast ion transport for low-temperature zinc batteries. Adv. Mater. **38**(14), e22750 (2026). 10.1002/adma.202522750 41603500 10.1002/adma.202522750

[123] J. Li, Y. Lou, S. Zhou, Y. Chen, X. Zhao et al., Intrinsically decoupled coordination chemistries enable quasi-eutectic electrolytes with fast kinetics toward enhanced zinc-ion capacitors. Angew. Chem. Int. Ed. **63**(34), e202406906 (2024). 10.1002/anie.202406906 10.1002/anie.202406906 38819764

[124] S. Chen, D. Ji, Q. Chen, J. Ma, S. Hou et al., Coordination modulation of hydrated zinc ions to enhance redox reversibility of zinc batteries. Nat. Commun. **14**(1), 3526 (2023). 10.1038/s41467-023-39237-3 37316539 10.1038/s41467-023-39237-3 PMC10267194

[125] K. Wang, T. Qiu, L. Lin, H. Zhan, X.-X. Liu et al., Solvation sheath regulation to induce sulfide solid–electrolyte interphase on Zn metal anode. ACS Energy Lett. **9**(3), 1000–1007 (2024). 10.1021/acsenergylett.4c00318

[126] S. Qin, R. Qi, Y. Wang, Y. Hu, M. Ma et al., A jointly triggered H_2_ evolution model modulated polyanionic hydrogel electrolyte for reversible Zn chemistry. Adv. Funct. Mater. **35**(35), 2505946 (2025). 10.1002/adfm.202505946

[127] X. Wang, W. Zhou, L. Wang, Y. Zhang, S. Li et al., Benchmarking corrosion with anionic polarity index for stable and fast aqueous batteries even in low-concentration electrolyte. Adv. Mater. **37**(14), e2501049 (2025). 10.1002/adma.202501049 40025972 10.1002/adma.202501049

[128] B. Ye, F. Wu, R. Zhao, H. Zhu, M. Lv et al., Electrolyte regulation toward cathodes with enhanced-performance in aqueous zinc ion batteries. Adv. Mater. **37**(15), e2501538 (2025). 10.1002/adma.202501538 40033963 10.1002/adma.202501538

[129] Y. An, C. Shu, Y. Liu, Y. Xu, L. Kang et al., Modulating the hydrogen bond for a stable zinc anode with a wide temperature range via the sucrose and polyacrylamide synergistic effect. ACS Nano **19**(11), 11146–11163 (2025). 10.1021/acsnano.4c18178 40074528 10.1021/acsnano.4c18178

[130] R. Chen, C. Zhang, J. Li, Z. Du, F. Guo et al., A hydrated deep eutectic electrolyte with finely-tuned solvation chemistry for high-performance zinc-ion batteries. Energy Environ. Sci. **16**(6), 2540–2549 (2023). 10.1039/d3ee00462g

[131] X. Ji, A perspective of ZnCl_2_ electrolytes: the physical and electrochemical properties. eScience **1**(2), 99–107 (2021). 10.1016/j.esci.2021.10.004

[132] Y. Dong, H. Hu, P. Liang, L. Xue, X. Chai et al., Dissolution, solvation and diffusion in low-temperature zinc electrolyte design. Nat. Rev. Chem. **9**(2), 102–117 (2025). 10.1038/s41570-024-00670-7 39775526 10.1038/s41570-024-00670-7

[133] J. Ma, C. Li, Q. Ji, C. Liu, B. Tang et al., V-induced low-spin state Mn^3+^ suppresses Jahn-Teller distortion for high-performance aqueous zinc ion batteries. Angew. Chem. Int. Ed. **64**(44), e202513148 (2025). 10.1002/anie.202513148 10.1002/anie.202513148 40905448

[134] Z.J. Chen, T.Y. Shen, M.H. Zhang, X. Xiao, H.Q. Wang et al., Tough, anti-fatigue, self-adhesive, and anti-freezing hydrogel electrolytes for dendrite-free flexible zinc ion batteries and strain sensors. Adv. Funct. Mater. **34**(26), 2314864 (2024). 10.1002/adfm.202314864

[135] Q. Zhou, F. Zhang, Z. Tan, T. Wang, D.C. Qi et al., Gradient hydrogel electrolyte enables high ionic conductivity and robust mechanical properties for dendrite-free aqueous zinc-ion battery. Adv. Mater. **38**(6), e12775 (2026). 10.1002/adma.202512775 41195892 10.1002/adma.202512775

[136] W. Yin, Y. Pan, J. Luo, Z. Li, S. Liang et al., Understanding the entropy decoupling for ion transport in ordered biomimetic materials toward durable aqueous zinc metal batteries. Angew. Chem. Int. Ed. **65**(7), e22157 (2026). 10.1002/anie.202522157 10.1002/anie.202522157 41403325

[137] L. Ye, J. Wei, S. Yuan, J. Wang, Y. Wang et al., High-entropy polymeric electrolytes facilitating ion conduction and interfacial desolvation in low-temperature zinc batteries. J. Am. Chem. Soc. **148**(7), 7605–7613 (2026). 10.1021/jacs.5c21446 41697718 10.1021/jacs.5c21446

[138] M. Qiu, Y. Liang, J. Hong, J. Li, P. Sun et al., Entropy-driven hydrated eutectic electrolytes with diverse solvation configurations for all-temperature Zn-ion batteries. Angew. Chem. Int. Ed. **63**(38), e202407012 (2024). 10.1002/anie.202407012 10.1002/anie.202407012 38943544

[139] H. Su, E. Hinks, Y. Chen, S.C. Kim, Y. Li et al., High-entropy second solvation shell by anion diversity for aqueous zinc-ion electrolytes. ACS Energy Lett. **11**(4), 3265–3273 (2026). 10.1021/acsenergylett.5c04154

[140] Z. Gong, Q. Meng, Y. Zhao, C. Wang, W. Wang et al., A high-entropy hydrogel electrolyte generated by intrinsically disordered polymer segments for efficient zinc metal batteries. Angew. Chem. Int. Ed. **65**(7), e23881 (2026). 10.1002/anie.202523881 10.1002/anie.202523881 41472407

[141] J. Zhang, T. Liu, X. Dong, Z. Chen, B. Tang et al., Electrolyte coordination environments in wide-temperature aqueous metal batteries: mechanisms and design strategies. Chem. Sci. **17**(3), 1569–1582 (2026). 10.1039/d5sc08605a 41522866 10.1039/d5sc08605a PMC12784501

[142] W. Luo, H. Fu, P. Peng, C. Gao, M. Gu et al., Solvation-configurational entropy governs interfacial kinetics in low-temperature batteries. Joule **10**(3), 102271 (2026). 10.1016/j.joule.2025.102271

[143] B. Zhang, X. Cai, S. Tian, J. Liang, M.H. Helal et al., Zwitterionic hydrogels endow zinc‐ion micro‐batteries with superior durability for electrophysiological monitoring. Adv. Energy Mater. **15**(40), e03986 (2025). 10.1002/aenm.202503986

[144] X. Zhang, J. Li, F. Xie, X. Xu, X. Sun et al., Polyzwitterionic gel electrolyte: dual optimization of polyiodide shuttle suppression and anode stabilization in aqueous Zn‐I2 batteries. Adv. Funct. Mater. **35**(39), 2505132 (2025). 10.1002/adfm.202505132

[145] M. Zhang, W. Xu, X. Han, H. Fan, T. Chen et al., Unveiling the mechanism of the dendrite nucleation and growth in aqueous zinc ion batteries. Adv. Energy Mater. **14**(9), 2303737 (2023). 10.1002/aenm.202303737

[146] J. Xu, Y. Zhu, Q. Gui, S. Sun, P. Zhao et al., High-performance fatigue-resistant dual-polyrotaxane hydrogel electrolytes for flexible aqueous zinc-ion batteries. Small **21**(14), e2500124 (2025). 10.1002/smll.202500124 40033872 10.1002/smll.202500124

[147] L. Huang, J. Xia, Z. Jin, X. Hu, X. Chen et al., Entropy-driven toughening and closed-loop recycling of polymers via divergent metal-pyrazole interactions. Nat. Commun. **16**(1), 10673 (2025). 10.1038/s41467-025-65700-4 41309603 10.1038/s41467-025-65700-4 PMC12661056

[148] Z. Shao, L. Lin, W. Zhuang, S. Liu, P. Yang et al., In situ self-reconfiguration induced multifunctional triple-gradient artificial interfacial layer toward long-life Zn-metal anodes. Adv. Mater. **36**(32), e2406093 (2024). 10.1002/adma.202406093 38865651 10.1002/adma.202406093

[149] Y. Wang, B. Liang, D. Li, Y. Wang, C. Li et al., Joule **9**(6), 101944 (2025). 10.1016/j.joule.2025.101944

[150] J. Wu, Y. Tang, H. Xu, G. Ma, J. Jiang et al., ZnO additive boosts charging speed and cycling stability of electrolytic Zn-Mn batteries. Nano-Micro Lett. **16**(1), 74 (2024). 10.1007/s40820-023-01296-y 10.1007/s40820-023-01296-y PMC10767122 38175408

[151] Y. Cheng, Y. Jiao, P. Wu, Manipulating Zn 002 deposition plane with zirconium ion crosslinked hydrogel electrolyte toward dendrite free Zn metal anodes. Energy Environ. Sci. **16**(10), 4561–4571 (2023). 10.1039/d3ee02114a

[152] H. Hong, Q. Nian, X. Guo, Q. Li, C. Zhi, Electrolyte design for aqueous batteries. Sci. Adv. **12**(6), eaeb4498 (2026). 10.1126/sciadv.aeb4498 10.1126/sciadv.aeb4498 PMC12871453 41637504

[153] W. Zhu, Z. Lei, P. Wu, Multi-stage collaborative design of hierarchical twisted hydrogel electrolytes for aqueous zinc-ion batteries with high capacity, ultralong stability, and mechanical robustness. Energy Environ. Sci. **18**(8), 3647–3658 (2025). 10.1039/d5ee00001g

[154] Z. Xing, P. Ye, X. Shi, L. Shan, S. Guo et al., Design of cryogenic electrolyte with organic-free solvation structure for wide-temperature zinc metal batteries. Angew. Chem. Int. Ed. **64**(49), e202516974 (2025). 10.1002/anie.202516974 10.1002/anie.202516974 41090446

[155] Q. Meng, Q. Bai, R. Zhao, P. Cao, G. Zhang et al., Attenuating water activity through impeded proton transfer resulting from hydrogen bond enhancement effect for fast and ultra-stable Zn metal anode. Adv. Energy Mater. **13**(44), 2302828 (2023). 10.1002/aenm.202302828

[156] J. Hu, Y. Qu, F. Shi, J. Wang, X. He et al., Enhancing the kinetics of zinc ion deposition by catalytic ion in polymer electrolytes for advanced Zn–MnO_2_ batteries. Adv. Funct. Mater. **32**(52), 2209463 (2022). 10.1002/adfm.202209463

[157] F. Bu, Y. Gao, W. Zhao, Q. Cao, Y. Deng et al., Bio-inspired trace hydroxyl-rich electrolyte additives for high-rate and stable Zn-ion batteries at low temperatures. Angew. Chem. Int. Ed. **63**(9), e202318496 (2024). 10.1002/anie.202318496 10.1002/anie.202318496 38180310

[158] Y. Wang, H. Zhang, Y. Chen, X. Tian, D. Xu et al., Hydrogel electrolytes for temperature robust aqueous zinc‐ion batteries. Adv. Energy Mater. **15**(47), e03226 (2025). 10.1002/aenm.202503226

[159] X. Li, D. Wang, F. Ran, Key approaches and challenges in fabricating advanced flexible zinc-ion batteries with functional hydrogel electrolytes. Energy Storage Mater. **56**, 351–393 (2023). 10.1016/j.ensm.2023.01.034

[160] Z. Zhang, Y. Mu, L. Xiao, H. Hu, T. Xue et al., Hydrogel electrolytes for zinc-ion batteries: materials design, functional strategies, and future perspectives. Nano-Micro Lett. **18**(1), 139 (2026). 10.1007/s40820-025-01993-w 10.1007/s40820-025-01993-w PMC12765786 41486346

[161] J. Yin, Z. Wu, S. Zhang, X. Liu, Y. Liu et al., A review of the water dilemma and hydrogen-bond chemistry for aqueous zinc-ion batteries. Energy Environ. Sci. **18**(24), 10263–10285 (2025). 10.1039/d5ee05775b

[162] J. Li, H. Zhang, Z. Liu, H. Du, H. Wan et al., Boosting dendrite‐free zinc anode with strongly polar functional group terminated hydrogel electrolyte for high‐safe aqueous zinc‐ion batteries. Adv. Funct. Mater. **35**(2), 2412865 (2024). 10.1002/adfm.202412865

[163] Z. Yan, F. Luo, S. Yang, Q. Wu, J. Zhang et al., Polydentate ligand‐induced surface reconstruction of MIL‐88A reinforced gel electrolytes for highly reversible zinc batteries via ion rectification and charge redistribution. Adv. Funct. Mater. **36**(3), e14679 (2025). 10.1002/adfm.202514679

[164] W. Song, L. Ye, J. Peng, Q. Ma, B. Wu et al., Enabling high‐performance Zn anodes through nitrogen‐participated p–π conjugation in hydrogel electrolytes. Adv. Energy Mater. **15**(36), e03484 (2025). 10.1002/aenm.202503484

[165] Z. Liu, Y. Zhang, M. Li, H. Li, J. Zhang et al., Lean‐water gel electrolyte enables zinc ion battery at −70 °C. Angew. Chem. Int. Ed. **64**(47), e202511520 (2025). 10.1002/anie.202511520 10.1002/anie.202511520 40985751

[166] G. Li, Q. Cai, S. Zhang, J.A. Yuwono, L. Mao et al., Decoupled dual-salt electrolyte for practical aqueous zinc batteries. Nat. Sustain. **8**(11), 1349–1359 (2025). 10.1038/s41893-025-01646-1

[167] C. Jiang, Y. Zhang, M. Jia, L. Li, Q. Hou et al., Anti-freezing hydrogel electrolytes: from molecular engineering to applications in aqueous zinc-ion batteries. Mater. Today **90**, 706–723 (2025). 10.1016/j.mattod.2025.09.007

[168] Y. Lei, F. Liu, L. Chen, M. Xu, Y. Hu et al., Polyanionic hydrogel electrolytes to regulate ion transport behavior in long cycle life zinc-ion batteries. Nano Energy **143**, 111284 (2025). 10.1016/j.nanoen.2025.111284

[169] X. Wang, X. Zhang, X. Kuang, D. Wang, C. Chang et al., Molecular engineering of hydrogel polymer electrolytes for climate‐resilient rechargeable batteries. Adv. Energy Mater. **0**, e06515 (2026). 10.1002/aenm.202506515

[170] M. Shi, J. Zhang, G. Tang, B. Wang, S. Wang et al., Polyzwitterionic cross-linked double network hydrogel electrolyte enabling high-stable Zn anode. Nano Res. **17**(6), 5278–5287 (2024). 10.1007/s12274-024-6525-5

[171] Z. Du, S. Shen, X. Su, Y. Zhuang, M. Chen et al., A robust and tough composite hydrogel electrolyte with anion-locked supramolecular network for zinc ion batteries. Adv. Mater. **37**(24), e2502328 (2025). 10.1002/adma.202502328 40244083 10.1002/adma.202502328

[172] C. Wang, Z. Gong, J.A. Yuwono, Q. Meng, Y. Lyu et al., Ligand-channel-induced ion liberation in crowded zwitterionic hydrogel electrolyte for efficient zinc metal batteries. Nat. Commun. **16**(1), 11069 (2025). 10.1038/s41467-025-66041-y 41381468 10.1038/s41467-025-66041-y PMC12698778

[173] Z. Cui, T. Xu, T. Yao, G. Mao, X. He et al., Tailoring acid-salt hybrid electrolyte structure for stable proton storage at ultralow temperature. Adv. Mater. **37**(7), e2412104 (2025). 10.1002/adma.202412104 39737663 10.1002/adma.202412104

[174] Y. Shi, R. Wang, S. Bi, M. Yang, L. Liu et al., An anti‐freezing hydrogel electrolyte for flexible zinc‐ion batteries operating at −70 °C. Adv. Funct. Mater. **33**(24), 2214546 (2023). 10.1002/adfm.202214546

[175] L. Yu, J. Huang, S. Wang, L. Qi, S. Wang et al., Ionic liquid “water pocket” for stable and environment-adaptable aqueous zinc metal batteries. Adv. Mater. **35**(21), e2210789 (2023). 10.1002/adma.202210789 36848503 10.1002/adma.202210789

[176] L. Yang, H. Ren, P. Lei, Y. Long, Z. Chen et al., Hydrophobic association enabled salt-concentrated all-cellulose hydrogel electrolytes for high-temperature zinc-ion hybrid supercapacitors. Angew. Chem. Int. Ed. **65**(6), e21038 (2026). 10.1002/anie.202521038 10.1002/anie.202521038 41410290

[177] C. Wang, X. Zeng, J. Qu, J.M. Cairney, Q. Meng et al., Salt-tolerance training enabled flexible molten hydrate gel electrolytes for energy-dense and stable zinc storage. Matter **6**(11), 3993–4012 (2023). 10.1016/j.matt.2023.08.019

[178] J. Nan, Y. Sun, F. Yang, Y. Zhang, Y. Li et al., Coupling of adhesion and anti-freezing properties in hydrogel electrolytes for low-temperature aqueous-based hybrid capacitors. Nano-Micro Lett. **16**(1), 22 (2023). 10.1007/s40820-023-01229-9 10.1007/s40820-023-01229-9 PMC10661583 37982913

[179] Y. Sang, J. Wang, M. Xu, B. Zhang, Q. Huang et al., Engineering robust hydrophilic–hydrophobic interface via π‐electron delocalization for ultralong‐lived zinc–ion batteries. Angew. Chem. Int. Ed. **64**(31), e202506984 (2025). 10.1002/anie.202506984 10.1002/anie.202506984 40421874

[180] S.-J. Guo, M.-Y. Yan, D.-M. Xu, P. He, K.-J. Yan et al., Anti-freezing hydrogel electrolyte with a regulated hydrogen bond network enables high-rate and long cycling zinc batteries. Energy Environ. Sci. **18**(1), 418–429 (2025). 10.1039/d4ee02772h

[181] W. Ling, F. Mo, X. Wu, X. Zeng, J. Xiong et al., Solid-state eutectic electrolyte via solvation regulation for voltage-elevated and deep-reversible Zn batteries. Nat. Commun. **16**(1), 4868 (2025). 10.1038/s41467-025-60125-5 40419480 10.1038/s41467-025-60125-5 PMC12106649

[182] X. Yang, L. Zhang, J. Zhu, L. Wang, Y. Zhang et al., Lewis acid-base effect and protonation in electrolyte engineering enable shuttle-free, dendrite-free, and HER-free aqueous Zn-I_2_ batteries. Energy Storage Mater. **78**, 104268 (2025). 10.1016/j.ensm.2025.104268

[183] X. Shi, Y. Zhong, Y. Yang, J. Zhou, X. Cao et al., Anion-anchored polymer-in-salt solid electrolyte for high-performance zinc batteries. Angew. Chem. Int. Ed. **64**(2), e202414777 (2025). 10.1002/anie.202414777 10.1002/anie.202414777 39325543

[184] D. Lin, J. Li, M. Wang, M. Jian, R. Pan et al., Nanofluidic-engineered carbon nanotube ion highways in hydrogels enable high-power aqueous zinc-ion batteries. Sci. Adv. **11**(47), eadx9812 (2025). 10.1126/sciadv.adx9812 41259502 10.1126/sciadv.adx9812 PMC12629143

[185] Q. Wang, J. Huang, L. Qi, M. Li, S. Wang et al., A bioinspired gradient hydrogel electrolyte network with optimized interfacial chemistry toward robust aqueous zinc-ion batteries. ACS Nano **19**(29), 26770–26781 (2025). 10.1021/acsnano.5c06914 40679095 10.1021/acsnano.5c06914

[186] Y. Lin, S. Wang, J. Huang, L. Chen, T. Bi et al., Coupling of mechanical, self‐healing, adhesion, and high‐ion conducting properties in anti‐freezing hydrogel electrolytes of zinc ion batteries via Fe^3+^‐carboxylate coordination. Adv. Funct. Mater. **35**(37), 2504726 (2025). 10.1002/adfm.202504726

[187] R. Han, Y. Meng, X. Zhao, Y. Wang, M. Tang et al., Energy Environ. Sci. **18**(11), 5482–5491 (2025). 10.1039/d5ee01478f

[188] T. Shen, Z.J. Chen, Y. Yang, Q. Yu, J. Wang et al., Thinner-than-paper and broad-temperature-adaptive zinc-iodine batteries enabled by nanophase separated deep-eutectic hydrogel electrolytes. Nat. Commun. **17**, 71312 (2026). 10.1038/s41467-026-71312-3 10.1038/s41467-026-71312-3 PMC13223333 41932920

[189] M. Chen, G. Chen, C. Sun, X. Li, M. Zhang et al., SiO_2_ nanoparticles-induced antifreezing hydrogel electrolyte enables Zn-I_2_ batteries with complete and reversible four-electron-transfer mechanisms at low temperatures. Angew. Chem. Int. Ed. **64**(21), e202502005 (2025). 10.1002/anie.202502005 10.1002/anie.202502005 40090996

[190] Y. Wang, L.N. Song, S. Liang, J.Y. Wu, L. Feng et al., Design considerations of ionic conductive elastomeric electrolyte for solid-state zinc metal batteries with high safety and long life. Angew. Chem. Int. Ed. **64**(30), e202507137 (2025). 10.1002/anie.202507137 10.1002/anie.202507137 40401814

[191] C. Yan, Y. Wang, X. Deng, Y. Xu, Cooperative chloride hydrogel electrolytes enabling ultralow-temperature aqueous zinc ion batteries by the Hofmeister effect. Nano-Micro Lett. **14**(1), 98 (2022). 10.1007/s40820-022-00836-2 10.1007/s40820-022-00836-2 PMC8993986 35394219

[192] Z. Gong, D. Kong, N. Chen, F. Zhang, D. Wang et al., Theoretical insights into aqueous zinc metal batteries: applications of density functional theory and molecular dynamics simulations in electrolyte design. Adv. Funct. Mater. **36**(10), e13415 (2025). 10.1002/adfm.202513415

[193] T. Zhang, J. Yu, T. Lin, Y. Song, M. Li et al., Fluorinated deep eutectic gel electrolytes for sustainable lithium metal batteries. J. Am. Chem. Soc. **147**(36), 32861–32872 (2025). 10.1021/jacs.5c08642 40839969 10.1021/jacs.5c08642

[194] L. Yu, S. Wang, J. Huang, X. Zeng, X. Zhou et al., Experimentally tracing water molecules and mapping high-resolution liquid structure pictures for promising water-in-salt/ionic liquid electrolytes. Natl. Sci. Rev. **13**(8), nwag125 (2026). 10.1093/nsr/nwag125 42078950 10.1093/nsr/nwag125 PMC13131236

[195] C. Yang, P. Woottapanit, S. Geng, R. Chanajaree, Y. Shen et al., A multifunctional quasi-solid-state polymer electrolyte with highly selective ion highways for practical zinc ion batteries. Nat. Commun. **16**(1), 183 (2025). 10.1038/s41467-024-55656-2 39747185 10.1038/s41467-024-55656-2 PMC11697030

[196] J.-H. Kim, N.-Y. Kim, Z. Ju, Y.-K. Hong, K.-D. Kang et al., Upscaling high-areal-capacity battery electrodes. Nat. Energy **10**(3), 295–307 (2025). 10.1038/s41560-025-01720-0

[197] A. Innocenti, S. Beringer, S. Passerini, Cost and performance analysis as a valuable tool for battery material research. Nat. Rev. Mater. **9**(5), 347–357 (2024). 10.1038/s41578-024-00657-2

[198] Z. Zhao, X. Liu, M. Zhang, L. Zhang, C. Zhang et al., Development of flow battery technologies using the principles of sustainable chemistry. Chem. Soc. Rev. **52**(17), 6031–6074 (2023). 10.1039/d2cs00765g 37539656 10.1039/d2cs00765g

[199] H. Dong, J. Li, J. Guo, F. Lai, F. Zhao et al., Insights on flexible zinc-ion batteries from lab research to commercialization. Adv. Mater. **33**(20), e2007548 (2021). 10.1002/adma.202007548 33797810 10.1002/adma.202007548

[200] P. Wang, J. Wang, L. Bai, N. Li, C. Zhou et al., Bioinspired injection therapy for spent LiFePO_4_ batteries: a non-invasive strategy for capacity regeneration and longevity enhancement. Nano-Micro Lett. **18**(1), 245 (2026). 10.1007/s40820-026-02091-1 10.1007/s40820-026-02091-1 PMC12886631 41661419

[201] J. Huang, S. Wang, J. Chen, C. Chen, E. Lizundia, Environmental sustainability of natural biopolymer-based electrolytes for lithium ion battery applications. Adv. Mater. **37**(22), e2416733 (2025). 10.1002/adma.202416733 39757715 10.1002/adma.202416733 PMC12138847

